# Development of novel Copper(II)–hydrazone complexes for targeting the inhibition of human osteosarcoma growth

**DOI:** 10.3389/fchem.2026.1897476

**Published:** 2026-10-01

**Authors:** Lucía Santa Maria de la Parra, Olivia Espindola-Moreno, Matías H. Assandri, Gustavo A. Echeverría, Oscar E. Piro, Gonzalo Scalese, Ignacio E. León

**Affiliations:** 1 Departamento de Química, Facultad de Ciencias Exactas, CEQUINOR (UNLP, CCT-CONICET La Plata, asociado a CIC), Universidad Nacional de La Plata, La Plata, Argentina; 2 Departamento de Ciencias Biológicas, Cátedra de Microbiología, Facultad de Ciencias Exactas, Universidad Nacional de La Plata (UNLP), La Plata, Argentina; 3 Departamento de Física, Facultad de Ciencias Exactas, Universidad Nacional de La Plata, La Plata, Argentina; 4 Instituto IFLP (CONICET, CCT-La Plata), Universidad Nacional de La Plata, La Plata, Argentina; 5 Departamento Estrella Campos, Área Química Inorgánica, Facultad de Química, Universidad de la República, Montevideo, Uruguay; 6 Laboratory Redox Biology of Trypanosomes, Institut Pasteur de Montevideo, Montevideo, Uruguay; 7 Departamento de Ciencias Biológicas, Cátedra de Fisiopatología, Facultad de Ciencias Exactas, Universidad Nacional de La Plata, La Plata, Argentina

**Keywords:** cancer, copper, hydrazones, osteosarcoma, reactive oxygen species

## Abstract

**Introduction:**

Osteosarcoma (OS) is the most common primary malignant bone tumor in children and young adults and remains associated with poor clinical outcomes due to recurrence, metastasis, and resistance to conventional chemotherapy. In recent years, copper-based compounds have emerged as promising anticancer agents because of their unique redox properties and ability to target multiple cellular pathways.

**Methods:**

In this work, we synthesized and characterized two hydrazone ligands, (*E*)-*N*′-(2-hydroxybenzylidene)furan-2-carbohydrazide (L1) and (*E*)-*N*′-(2-hydroxybenzylidene)-4-methoxybenzohydrazide (L2), together with their corresponding Cu(II) complexes, CuL1 and Cu_2_L2_2_. The compounds were characterized by elemental analysis, spectroscopic techniques, high-resolution mass spectrometry, and single-crystal X-ray diffraction. The biological activity of the complexes was evaluated in human MG-63 osteosarcoma cells using the MTT assay. Reactive oxygen species (ROS) generation was assessed using dihydrorhodamine 123 (DHR123) after 24 h of treatment to gain insight into their mechanism of action. Their activity was further evaluated in 3D MG-63 osteosarcoma spheroids using a resazurin-based viability assay, while anti-migratory effects were assessed using the spheroid spreading assay.

**Results:**

Biological evaluation demonstrated that coordination to Cu(II) converted the inactive hydrazone ligands into highly potent anticancer agents. Both Cu(II) complexes exhibited potent cytotoxic activity in 2D cell cultures, with IC_50_ values in the low micromolar range and significantly higher activity than cisplatin, whereas the free ligands showed no significant antiproliferative effect. Furthermore, treatment with CuL1 and Cu_2_L2_2_ induced a rapid increase in intracellular ROS, suggesting that oxidative stress contributes to their mechanism of action. The antitumor potential of the complexes was further assessed using multicellular tumor spheroids, where both compounds reduced spheroid viability, altered spheroid morphology, and significantly inhibited spheroid-derived cell migration.

**Discussion:**

Among the compounds studied, the binuclear complex Cu_2_L2_2_ displayed the highest antiproliferative activity in both 2D and 3D models. Overall, our findings demonstrate that coordination of hydrazone ligands to Cu(II) markedly enhances their biological activity and identify CuL1 and particularly the binuclear complex Cu_2_L2_2_ as promising candidates for the development of new copper-based therapeutics against osteosarcoma.

## 1 Introduction

Cancer remains a leading cause of mortality worldwide, with osteosarcoma (OS) standing out as the most prevalent primary bone malignancy in children and young adults (Bray et al., 2024). OS is characterized by rapid proliferation, high invasiveness, and the production of immature osteoid matrix, which together contribute to poor prognosis (Kansara et al., 2014). Current treatment strategies combining surgical resection with multi-agent chemotherapy, commonly including methotrexate, doxorubicin, and cisplatin, have improved survival rates only modestly (Li et al., 2023). Despite these efforts, relapse and metastasis occur in a substantial fraction of patients, underscoring the urgent need for novel and more effective therapeutic approaches (Moreno et al., 2017).

In this context, metal-based drugs have emerged as a promising alternative, leveraging the unique chemical and biological properties of transition metals (León, 2024; Santa Maria de la Parra et al., 2024a). Their versatility stems from redox activity, coordination geometry, and the capacity to engage in diverse interactions with biomolecules, thereby enabling selective cytotoxicity in tumor cells (Ferretti and León, 2022; Lee et al., 2020). Among these, copper-based complexes have garnered significant attention due to their potent antitumor activity observed across multiple cancer models (Balsa et al., 2023a; Balsa et al., 2025). Copper compounds exert their anticancer activity through multiple mechanisms, including generation of reactive oxygen species (ROS), induction of oxidative DNA damage, proteasome inhibition, and selective targeting of cancer stem cells (Balsa et al., 2023b; Balsa et al., 2023c; Brooks et al., 2026; Del Bello et al., 2022; Fernández et al., 2024; Levín et al., 2021; Rodríguez et al., 2019; Romo et al., 2021). Importantly, the bioactivity of these compounds is often enhanced by rational ligand design, highlighting the critical role of metal–ligand coordination chemistry in modulating reactivity and specificity (Burgos-López et al., 2022).

Despite their promising attributes, only a limited number of copper complexes have been explored against OS, leaving a considerable gap in the development of bioinorganic strategies for this aggressive malignancy (Burgos-Lopez et al., 2019; Cadavid-Vargas et al., 2017). Therefore, the rational design, synthesis, and biological evaluation of copper-based metallodrugs represent a compelling avenue to overcome the limitations of conventional therapies and offer novel, mechanism-driven interventions for osteosarcoma.

*N*-acylhydrazones constitute a versatile class of ligands exhibiting notable pharmacological properties, including anticancer, antidiabetic, and antioxidant activities, largely attributed to their strong metal-chelating capabilities. Within the framework of a research program aimed at elucidating the relationship between ligand structure and anticancer activity, we have designed and synthesized a series of *N*-acylhydrazone ligands (L3–L9 ligands in Figure 1) and their corresponding copper (II) complexes. These compounds have demonstrated significant cytotoxicity against a range of solid tumors, with particularly pronounced activity in osteosarcoma and breast cancer models (Balsa et al., 2021; Balsa et al., 2023b; Balsa et al., 2023d; Balsa et al., 2024; Burgos-Lopez et al., 2019; Santa Maria de la Parra et al., 2026a; Santa Maria de la Parra et al., 2026b; Santa Maria de la Parra et al., 2024b; Santa Maria de la Parra et al., 2026).

**FIGURE 1:**
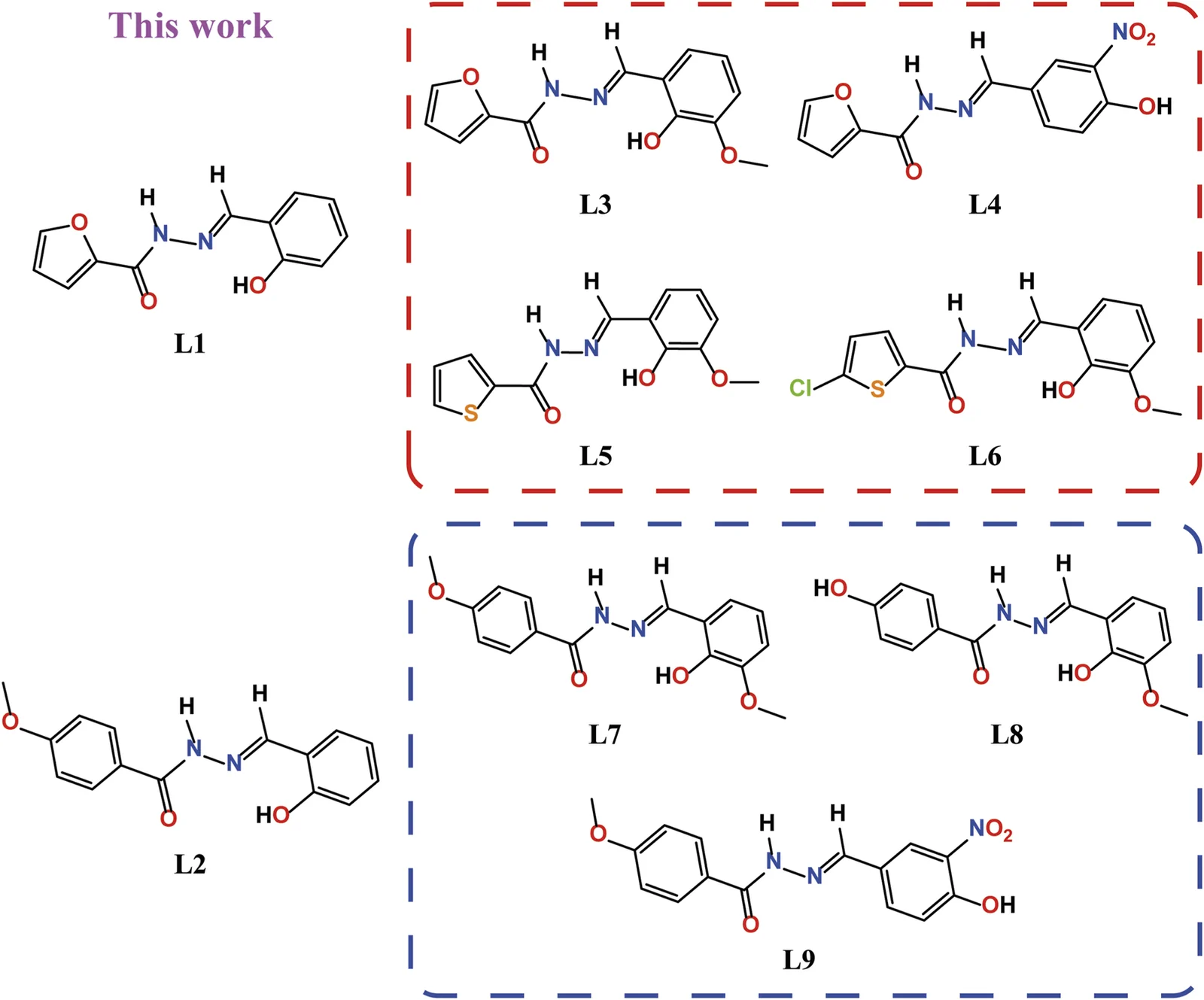
Schematic structures of ligands presented in this work (L1 and L2) and similar ligands with furan/thiophene (red line) or methoxy-benzo/hydroxy-benzo (blue line) hydrazide reported previously by our group.

Our previous studies have shown that structural differences in the hydrazide group of the ligand are reflected in the potency of their anticancer activity (furan = methoxy-benzohydrazide > hydroxy-benzohydrazide). Furthermore, we found that coligands such as o-phen and bipy enhance the biological activity of the complexes in osteosarcoma cells. Notably, the chlorine substitution in Cu_4_(L6)_4_ was shown to increase the potency of the cytotoxic effect by four times compared to CuL5, surpassing the effect of the change from furan to thiophene (Santa Maria de la Parra et al., 2026a). Since we found that the cytotoxic activity of the complexes increases with greater nuclearity (Espindola-Moreno et al., 2025), the observed effect of Cu_4_L6_4_ may be partially due to its higher Cu nuclearity than that of the monomeric complexes. Additionally, we found that tridentate compounds exhibited higher anticancer activity than structurally related bidentate complexes (Santa Maria de la Parra et al., 2026).

Building on these findings, we report here the synthesis and characterization of two novel Cu(II) coordination complexes (CuL1 and Cu_2_L2_2_) with the hydrazone ligands (*E*)-*N*'-(2-hydroxybenzylidene)furan-2-carbohydrazide (L1) and (*E*)-*N*'-(2-hydroxybenzylidene)-4-methoxybenzohydrazide (L2), together with their X-ray crystal structures. Furthermore, our study focuses on the assessment of the *in vitro* anticancer activity of the ligands and the new Cu(II) complexes using complementary 2D monolayer and 3D multicellular spheroid OS models derived from MG-63 osteosarcoma cells.

## 2 Materials and methods

### 2.1 Materials

Dulbecco’s modified Eagle medium (DMEM) and TrypLE™ were purchased from Gibco (Gaithersburg, MD, USA). Fetal bovine serum (FBS) was bought from Internegocios S.A. (Mercedes, Argentina). OS (MG-63) cell culture line was acquired from the American Type Culture Collection (ATCC). Tissue culture materials were purchased from Jet Bio-Filtration Co. (Guangzhou, China). Tetrazolium salt MTT (3-(4,5-dimethylthiazol-2-yl)-2,5-diphenyl-tetrazoliumbromide) was supplied by Invitrogen Co. (Buenos Aires, Argentina). Agarose was purchased from Inbio Highway, Argentina. Resazurin was purchased from Santa Cruz Biotechnology, Dallas, United States. Other reagents were of analytical or HPLC grade, obtained from commercial sources, and used as received from Merck (Darmstadt, Germany) or similar brands.

### 2.2 Methods

#### 2.2.1 Synthesis

##### 2.2.1.1 Synthesis of L1 and L2 ligands

The ligand L1 was synthesized by the condensation reaction of 2-furoic hydrazide (2-FH) with 2-hydroxybenzaldehyde (2-HBA), as shown in Figure 2. Similarly, to obtain L2, hydrazide 2-FH was replaced by 4-methoxybenzohydrazide (4-MBH), using the same aldehyde as for L1. For both syntheses, equimolar ethanolic solutions of hydrazide and aldehyde were heated and stirred separately. After adding the aldehyde solution dropwise to the hydrazide solution, the mixture was stirred under mild heating for 1 h. It was left to gradually evaporate at room temperature for 3 weeks. The needle-shaped light-yellow crystals were filtered off, washed with cold ethanol, and dried in a desiccator.

**FIGURE 2:**
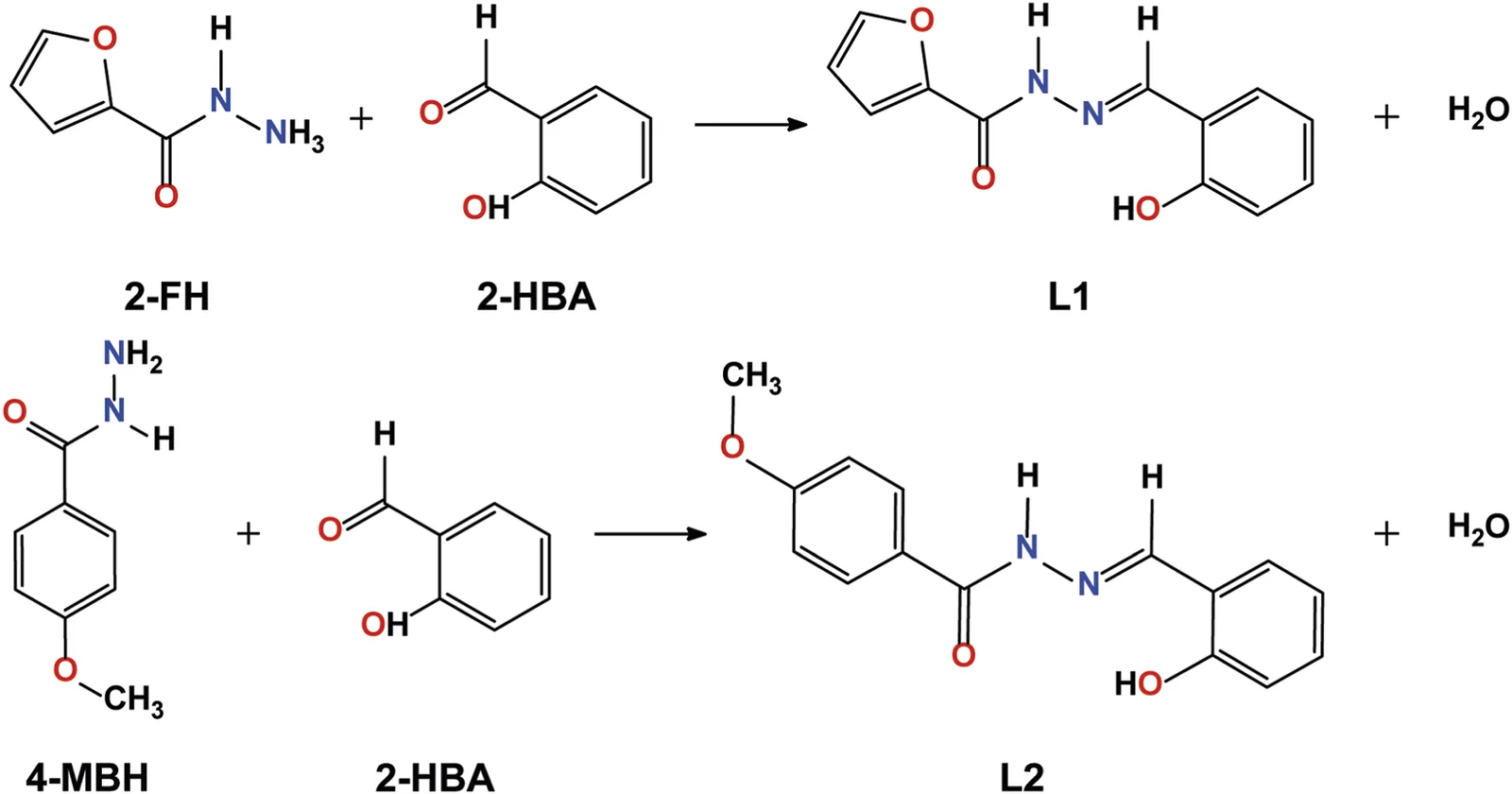
Schematic representation of the L1 and L2 ligand formation reaction.

L1·H_2_O: Yield 72% (0.179 g). Anal. (%) calc. for C_12_H_12_N_2_O_4_ (MW: 248.2): C, 58.07; H, 4.87; N, 11.29. Found: C, 58.24; H, 5.13; N, 11.64. ^1^H NMR (400 MHz, DMSO-*d*_6,_ δ/ppm): δ 12.11 (s, 1H, H10), 11.14 (s, 1H, H7), 8.64 (s, 1H, H8), 7.97 (d, *J* = 1.6 Hz, 1H, H15), 7.54 (d, *J* = 7.7 Hz, 1H, H6), 7.36–7.22 (m, 2H, H3, H13), 6.93 (d, *J* = 8.0 Hz, 2H, H4, H5), and 6.72 (dd, *J* = 3.5, 1.8 Hz, 1H, H14). ^13^C NMR (101 MHz, DMSO-*d*_6,_ δ/ppm): δ 157.81 (C11), 148.59 (C8), 144.73 (C15), 131.88 (C13), 129.77 (C6), 119.85 (C7), 116.85 (C4, C5), 115.70 (C3), and 112.64 (C14). HRMS (ESI+): m/z calc for C_12_H_11_N_2_O_3_ [M + H]^+^: 231.0764; found [M + H]^+^: 231.0751.

L2·H_2_O: Yield 85% (0.122 g). Anal. (%) calc. for C_15_H_16_N_2_O_4_ (MW: 288.3): C, 62.49; H, 5.59; N, 9.72. Found: C, 62.60; H, 5.81; N, 9.78^1^H NMR (400 MHz, DMSO-*d*_6,_ δ/ppm): δ 12.03 (s, 1H, H10), 11.42 (s, 1H, H7), 8.64 (s, 1H, H8), 7.96 (d, *J* = 8.5 Hz, 2H, H14, H16), 7.54 (dd, *J* = 7.7, 1.7 Hz, 1H, H6), 7.37–7.26 (m, 1H, H3), 7.14–7.05 (m, 2H, H13, H17), 7.01–6.88 (m, 2H, H4, H5), and 3.85 (s, 3H, H18). ^13^C NMR (101 MHz, DMSO, δ/ppm): 162.67 (C11), 157.93(C2), 148.33 (C8), 131.68 (C3), 130.06 (C14, C16, C6), 125.23 (C15), 119.77 (C1), 116.87 (C4, C5), 114.28 (C12, C13, C17), and 55.90 (C18). HRMS (ESI+): m/z calc for C_15_H_15_N_2_O_3_ [M + H]^+^: 271.1077; found [M + H]^+^: 271.1064.

##### 2.2.1.2 Synthesis of [Cu(L1-H) (NO_3_)]·H_2_O and [Cu_2_(μ-L2-H)_2_(NO_3_)_2_(CH_3_CH_2_OH)_2_] complexes

The complexes [Cu(L1-H) (NO_3_)] and [Cu_2_(μ-L2-H)_2_(NO_3_)_2_(CH_3_CH_2_OH)_2_], abbreviated as CuL1·H_2_O and Cu_2_L_2_ respectively, were synthesized following a common protocol, in which an ethanolic solution of copper (II) nitrate (0.25 mmol) was added dropwise to a solution containing 0.25 mmol of ligand L1 or ligand L2 in absolute ethanol under continuous stirring (Figure 3). The reaction was conducted under stirring at a moderate temperature (<50 °C) for 1 h, after which the solvent was allowed to evaporate slowly at room temperature for 6 weeks until dark-green crystals formed. The crystals were collected by filtration, washed with cold ethanol, and dried at room temperature.

**FIGURE 3:**
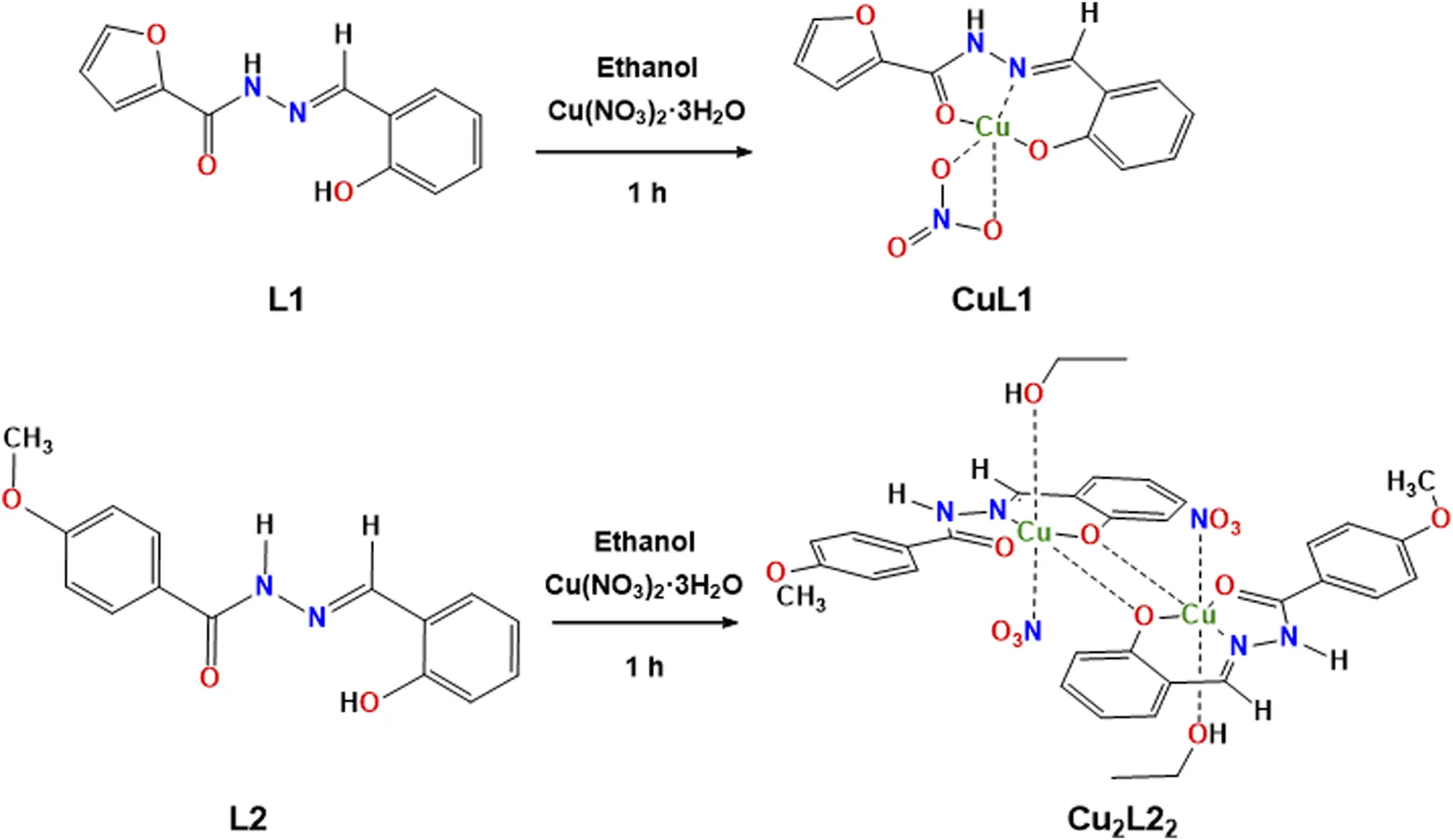
Depiction of synthetic procedures for Cu(II) complexes CuL1 and Cu_2_L2_2_ from their ligands L1 and L2, respectively.

CuL1·H_2_O: Yield 72% (0.067 g). Anal. (%) calc. for C_12_H_11_CuN_3_O_7_ (MW: 372.78): C, 38.66; H, 2.97; N, 11.27. Found: C, 38.69; H, 3.00; N, 11.33. HRMS (ESI-): m/z calc for C_12_H_8_CuN_3_O_6_ [Cu(L1-2H) (NO_3_)]^-^: 352.9715; found [Cu(L1-2H) (NO_3_)]^-^: 352.9720.

Cu_2_L2_2_: Yield 85% (0.094 g). Anal. (%) calc. for C_34_H_38_Cu_2_N_6_O_14_ (MW: 881.78): C, 46.31; H, 4.34; N, 9.53. Found: C, 46.35; H, 4.33; N, 9.55. HRMS (ESI+): m/z calc for C_30_H_26_Cu_2_N_4_O_6_ [Cu_2_(μ-L2-H)_2_]^2+^: 332.0217; found [Cu_2_(μ-L2-H)_2_]^2+^: 332.0206.

#### 2.2.2 Crystal data and structure refinement

##### 2.2.2.1 X-ray diffraction data

The measurements were performed on a Rigaku Oxford Gemini Eos CCD diffractometer with graphite-monochromated CuKα (λ = 1.54184 Å) radiation. X-ray diffraction intensities were collected (ω scans with ϑ and κ-offsets), integrated, and scaled using the CrysAlisPro suite of programs (Rigaku, 2015). The unit cell parameters were obtained by least-squares refinement (based on the angular setting for all collected reflections with intensities greater than seven times the standard deviation of measurement errors) using CrysAlisPro. Data were corrected empirically for absorption employing the multi-scan method implemented in CrysAlisPro.

L1·H_2_O. Most of the diffraction pattern was interpreted in terms of a two-component monoclinic twin. The unit cell parameters for both crystal domains were equal to within experimental accuracy. The domains were rotated from each other in 180° around the crystal *a*-axis. The reflections were indexed in the reciprocal unit cell of the corresponding domains. It was resorted to the twin crystal data reduction procedure of CrysAlisPro to generate two data sets, a regular one with the diffraction data indexed in the reciprocal unit cell of the largest domain (with approximately 70% of diffracting power) and a second one including all reflections from both domains with the overlapping ones flagged for structure development and refinement. The first dataset was employed to solve the structure using the dual procedure of SHELXT (Sheldrick, 2015) and its non-H atoms were refined with isotropic displacement parameters by a full-matrix least-squares procedure using SHELXL (Sheldrick, 2008).

Despite a correct molecular model, however, the refinement evidenced the presence of overlap between reflections from both crystal domains. The evidence included (besides the visual rendering with CrysAlisPro of the weighted reciprocal space) many observed violations of systematic extinctions (Ferraresi-Curotto et al., 2015), a relatively large agreement R1-factor (0.21), and the list of the most disagreeable reflections showing F (obs) values systematically larger than the corresponding F (calc) values. We, therefore, refined the initial molecular model against the second dataset, which included all collected reflections for both crystal domains, employing the untwining procedure of SHELXL. Now that there were no systematic extinction violations, the R1-factor dropped to 0.11, and the sign of F (obs)-F (calc) difference for the most disagreeable reflections was more evenly distributed. Following anisotropic refinement, a difference Fourier map showed all H-atoms except the one of a heterocycle ring. All but the water H-atoms were positioned at their expected locations and refined with the riding model. The hydroxyl H-atoms were refined by treating the O-H group as a rigid body allowed to rotate around the >C (ph)-OH bond to maximize the residual electron density at their calculated positions. A difference Fourier map phased on all determined atoms showed the water H-atoms among the first four peaks. These were refined at their found positions with isotropic displacement parameters. The diffracting weight of the minor crystal domain was 0.285 (1).

CuL1·H_2_O. The ligand hydrogen atoms were positioned at their expected locations and refined with the riding model. The water H-atoms were in a difference Fourier map and refined at their found positions with isotropic displacement parameters and Ow-H and H … H distances restrained to target values of 0.86 (1) and 1.36 (1) Å, respectively.

L2·H_2_O. The H-atoms were in a different Fourier map phased on the heavier atoms and refined at their found positions with isotropic displacement parameters.

Cu_2_L2_2_. The hydrogen atoms, except the one of the alcohol groups, were positioned at their expected locations and refined with the riding model. The methyl angular conformation was optimized by treating -CH_3_ as a rigid group allowed to rotate around the O-CH_3_ bond, such as maximizing the sum of the residual electron density at the calculated hydrogen positions. The alcohol H-atom of the EtOH ligand was in a different Fourier map and refined with an isotropic displacement parameter at its found position, with O-H and H_2_C(eth)…H distances restrained to target values of 0.86 (1) and 1.83 (1) Å, respectively.

Crystal data and structure refinement results are summarized in Tables 1, 2. Crystallographic structural data have been deposited at the Cambridge Crystallographic Data Centre (CCDC). Any request to the Cambridge Crystallographic Data Centre for this material should quote the full literature citation and the reference numbers CCDC 2306862 (L1.H_2_O), 2392963 (CuL1.H_2_O), 2392965 (L2.H_2_O), and 2392966 (Cu_2_L2_2_).

**TABLE 1:** Crystal data and structure refinement results for the L1 ligand and its Cu(II) complex.

Compound	L1·H_2_O	CuL1·H_2_O
Empirical formula	C_12_H_12_N_2_O_4_	C_12_H_11_CuN_3_O_7_
Formula weight	248.24	372.78
Temperature (K)	293 (2)	295 (2)
Wavelength (Å)	1.54184	1.54184
Crystal system	Monoclinic	Orthorhombic
Space group	*P*2_1/c_	*P*2_1_2_1_2_1_
Unit cell dimensions		
a (Å)	4.6682 (2)	5.6318 (1)
b (Å)	19.915 (1)	15.1761 (2)
c (Å)	25.803 (1)	16.2969 (2)
β (°)	94.712 (4)	90.00
Volume (Å^3^) *Z*	2390.7 (2) 8	1392.88 (4) 4
Density, calc. (Mg/m^3^)	1.379	1.778
Absorption coefficient (mm^-1^)	0.886	2.643
F (000)	1040	756
Crystal shape/color	Needle/colorless	Fragment/green
Crystal size (mm^3^) ϑ-range (°) for data collection	0.569 × 0.163 × 0.054 3.437 to 72.971	0.367 × 0.242 × 0.142 3.980 to 72.434
Index ranges	−3≤h ≤ 5, −24 ≤ k ≤ 24, −31 ≤ l ≤ 31	−5≤h ≤ 6, −18 ≤ k ≤ 18, −20 ≤ l ≤ 20
Reflections collected	5,120	12,384
Independent reflections	5,120 [R (int) = 0.0372]	2,730 [R (int) = 0.0280]
Observed reflections [I > 2σ(I)]	3,823	2,678
Completeness ϑ = 67.684° (%)	100.0	100.0
Data/restraints/parameters	5,120/0/344	2,730/3/220
Goodness-of-fit on F^2^	0.975	1.057
Final R indices^a^ [i > 2σ(i)]	R1 = 0.0589, wR2 = 0.1461	R1 = 0.0306, wR2 = 0.0858
R indices (all data)	R1 = 0.0757, wR2 = 0.1544	R1 = 0.0311, wR2 = 0.0865
Absolute structure parameter	​	−0.01 (1)
Larg. diff. peak and hole (e.Å-3)	0.231 and −0.218	0.626 and −0.416

^a^ R_1_ = Σ||F_o_|-|F_c_||/Σ|F_o_|, wR_2_ = [Σw (|F_o_|2-|Fc|2)2/Σw (|F_o_|2)2]1/2.

**TABLE 2:** Crystal data and structure refinement results for the L2 ligand and its Cu(II) complex.

Compound	L2·H_2_O	Cu_2_L2_2_
Empirical formula	C_15_H_16_N_2_O_4_	C_34_H_38_Cu_2_N_6_O_14_
Formula weight	288.30	881.78
Temperature (K)	295 (2)	295 (2)
Wavelength (Å)	1.54184	1.54184
Crystal system	Orthorhombic	Monoclinic
Space group	*P*ca2_1_	*P*2_1/c_
Unit cell dimensions		
a (Å)	12.708 (1)	11.5193 (1)
b (Å)	4.8265 (4)	7.8173 (1)
c (Å)	23.712 (2)	20.2533 (2)
β (°)	90.00	90.152 (1)
Volume (Å^3^) *Z*	1454.4 (2)	​
Density, calc. (Mg/m^3^)	4	1823.80 (4)
Absorption coefficient (mm^-1^)	2	​
F (000)	1.317	1.606
Crystal shape/color	Plate/colorless	Fragment/green
Crystal size (mm^3^) ϑ-range (°) for data collection	0.498 × 0.193 × 0.046 6.969 to 72.122	0.283 × 0.119 × 0.091 3.837 to 72.338
Index ranges	−14 ≤ h ≤ 15, −4≤k ≤ 5, −27 ≤ l ≤ 29	−14 ≤ h ≤ 7, −9≤k ≤ 9, −24 ≤ l ≤ 25
Reflections collected	3,657	7,290
Independent reflections	1854 [R (int) = 0.0354]	3,556 [R (int) = 0.0211]
Observed reflections [I > 2σ(I)]	1,580	3,159
Completeness ϑ = 67.684° (%)	99.9	100.0
Data/restraints/parameters	1,854/1/254	3,556/2/259
Goodness-of-fit on F^2^	1.028	1.033
Final R indices^a^ [i > 2σ(i)]	R1 = 0.0509, wR2 = 0.1398	R1 = 0.0357, wR2 = 0.1021
R indices (all data)	R1 = 0.0590, wR2 = 0.1519	R1 = 0.0406, wR2 = 0.1078
Absolute structure parameter	0.2 (5)	​
Larg. diff. peak and hole (e.Å^-3^)	0.198 and −0.210	0.403 and −0.302

^a^ R_1_ = Σ||F_o_|-|Fc||/Σ|F_o_|, wR_2_ = [Σw (|Fo|2-|Fc|2)2/Σw (|Fo|2)2]1/2.

##### 2.2.2.2 Physicochemical characterization

Elemental analyses for C, N, and H were performed using a Thermo Scientific Flash 2000 analyzer equipped with an Eager 200 data processor. The FT-IR spectra (4,000–400 cm^–1^) were recorded using KBr pellets on a Bruker Invenio-R. FT-IR spectrometer. The electronic spectra were recorded in a Shimadzu UV2600 Spectrophotometer. The 1D (^1^H and ^13^C) and 2D (^1^H–^1^H COSY, ^1^H–^13^C HSQC, and ^1^H–^13^C HMBC) NMR spectra were recorded in DMSO-*d*6 at 25 °C on a Bruker Avance III 400 MHz spectrometer. Chemical shifts are reported in ppm using the solvent residual peak as an internal standard for ^1^H NMR (δH 2.50 ppm) and ^13^C NMR (δC 39.52 ppm). HRMS was obtained in a Q Exactive Plus mass spectrometer (Thermo Scientific, United States) by direct injection using methanol as solvent and an Ion Max API source with a HESI-II probe. The mass spectrometer was operated in negative and positive modes; the ion spray voltage was set at 4.5 kV, and the capillary temperature was set at 250 °C.

#### 2.2.3 Cell culture conditions

The human OS (MG-63) cell line was cultured in Dulbecco’s modified Eagle medium (DMEM) supplemented with 10% fetal bovine serum (FBS), 100 IU/mL penicillin, and 100 μg/mL streptomycin at 37 °C in 5% CO_2_ atmosphere.

#### 2.2.4 2D studies

##### 2.2.4.1 Cell viability

A cytotoxic study was performed according to Mosmann (1983). Cells were seeded in a 96-well plate at a density of 3.5 × 10^4^ cells/mL, allowed to attach for 24 h, and then treated with different concentrations of ligands (10–100 µM) and complexes (0.5–3 µM) at 37 °C for 24 h. Afterward, the medium was replaced, and the cells were incubated with 0.5 mg/mL MTT under normal culture conditions for 3 h. Cell viability was assessed by the conversion of the tetrazolium salt MTT (3-(4,5-dimethylthiazol-2-yl)-2,5-diphenyl-tetrazoliumbromide) to a colored formazan by mitochondrial dehydrogenases. Color development was measured spectrophotometrically with a microplate reader (Multiplate Reader Multiskan FC, Thermo Scientific) at 570 nm after cell lysis in DMSO (100 µL per well). Cell viability was plotted as the percentage of the control value.

##### 2.2.4.2 Reactive oxygen species study

Oxidative stress in MG-63 cells was evaluated by measuring the intracellular production of ROS after treating the cell monolayer with different concentrations of Cu(II) complexes. In brief, the cells were grown in 24-well plates (10^5^ cells/well), incubated for 30 min in the dark with dihydrorhodamine-123 (DHR-123), and then treated for 3 h with increasing concentrations of the Cu(II) complexes at 37 °C in darkness. ROS generation was determined by the oxidation of DHR-123 to rhodamine by spectrofluorescence at 530 nm. Protein content was measured using the Pierce™ BCA Protein Assay Kit (Thermo Fisher Scientific, Waltham, Massachusetts, United States), and the results were adjusted accordingly.

#### 2.2.5 3D studies

##### 2.2.5.1 Multicellular tumor spheroid formation

MG-63 cells were used to create multicellular tumor spheroids using the modified hanging drop technique that we had previously presented (León et al., 2016; Ruiz et al., 2019). In brief, on the cell culture plate lid, 25 µL drops of cell suspension containing 400 cells each were suspended. Following the 72-h period needed for cell aggregation, the spheroids were transferred to 96-well plates coated with agarose (one droplet per well) and cultivated using 150 µL of complete medium. Spheroids were allowed to grow for another 48 h until reaching a diameter of 400 µm.

##### 2.2.5.2 Spheroid cell viability studies

Multi-cellular spheroids (MCS) were treated in 96-well plates with 0.5% DMSO in DMEM (control) and with Cu(II) complexes in a range of concentrations from 5 to 50 µM in DMEM for 24 h. Afterward, cell viability was evaluated using the resazurin reduction assay, in which the resazurin dye is irreversibly reduced by intracellular oxidoreductases to a pink-red fluorescent dye known as resorufin (Anoopkumar-Dukie et al., 2005). Fluorescence was measured using a Shimadzu RF-6000 fluorometer (excitation at 560 nm, emission at 590 nm). Cell viability was plotted as a percentage of the basal condition (solvent control).

##### 2.2.5.3 MCS spreading assay

To evaluate the migratory and proliferative capacity of spheroid-derived cells following 24 h exposure to CuL1 and Cu_2_L2_2_, spheroids were transferred to 48-well plates containing 500 µL of DMEM either alone (control) or supplemented with different concentrations of the complexes (1–10 µM) and incubated under standard culture conditions. Upon attachment of the spheroids to the plastic surface, cells initiated radial migration, progressively expanding the adhesion area. After 24 h of treatment, cells were fixed with methanol, stained with 20% Giemsa solution, and cell migration was assessed using an inverted microscope and ImageJ v1.53t software.

#### 2.2.6 Statistical analysis

Three independent experiments were performed, and the results are presented as the mean ± standard error of the mean (SEM), unless otherwise stated. Data normality was assessed using both the Shapiro–Wilk and D'Agostino–Pearson normality tests. Data that satisfied the normality assumption were analyzed using Student’s t-test (for comparisons between two groups) or one-way analysis of variance (ANOVA) followed by Tukey’s multiple comparisons test (for comparisons among three or more groups), as appropriate. Differences were considered statistically significant at *p* < 0.05. All statistical analyses were performed using GraphPad Prism version 8.0.1 (GraphPad Software Inc., San Diego, CA, United States).

## 3 Results

### 3.1 Crystallography

Crystal data and structure refinement results for L1·H_2_O, L2·H_2_O, CuL1·H_2_O, and CuL2 were described below.

L1·H_2_O. There are two closely related but independent molecules per crystal asymmetric unit (Z’ = 2, Z = 8), as shown in the ORTEP (Farrugia, 2012) drawing of Figure 4. Because of extended π-bond delocalization, both L1·H_2_O molecules are nearly planar (*rms* deviation of non-H atoms from the best least-squares plane less than 0.13 Å). Planarity is further favored by an intra-molecular OH … N bond [O … N bond distances of 2.671 (3) and 2.693 (3) Å and corresponding O-H … N bond angles of 146 and 144°]. Observed bond distances and angles conform to stablished organic chemistry knowledge and are listed in Supplementary Table S1. They are in close agreement with the corresponding data of the solid-state anhydrous organics reported by Bikas et al. (2010). The lattice is further stabilized by a complex network of medium-strength H-bonds. Neighboring organic molecules are bridged by crystallization water molecules acting as donor in intermolecular > C=O … HO_w_H … O=C< bonds [O_w_ … O_carb_ distances of 2.873 (3) and 2.807 (3) Å and corresponding O_w_-H … O_carb_ angles of 179 (5) and 166 (5)° for molecule #1 and Ow … O_carb_ distances of 2.827 (3) and 2.755 (3) Å and O_w_-H … O_carb_ angles of 167 (4) and 170 (4)° for #2] and as acceptor in >N-H … O_w_ bonds [N … O_w_ distances of 2.808 (3) and 2.864 (3) Å and N-H … Ow angles of 164 and 166°]. The H-bonding structure is further detailed in Supplementary Table S2.

**FIGURE 4:**
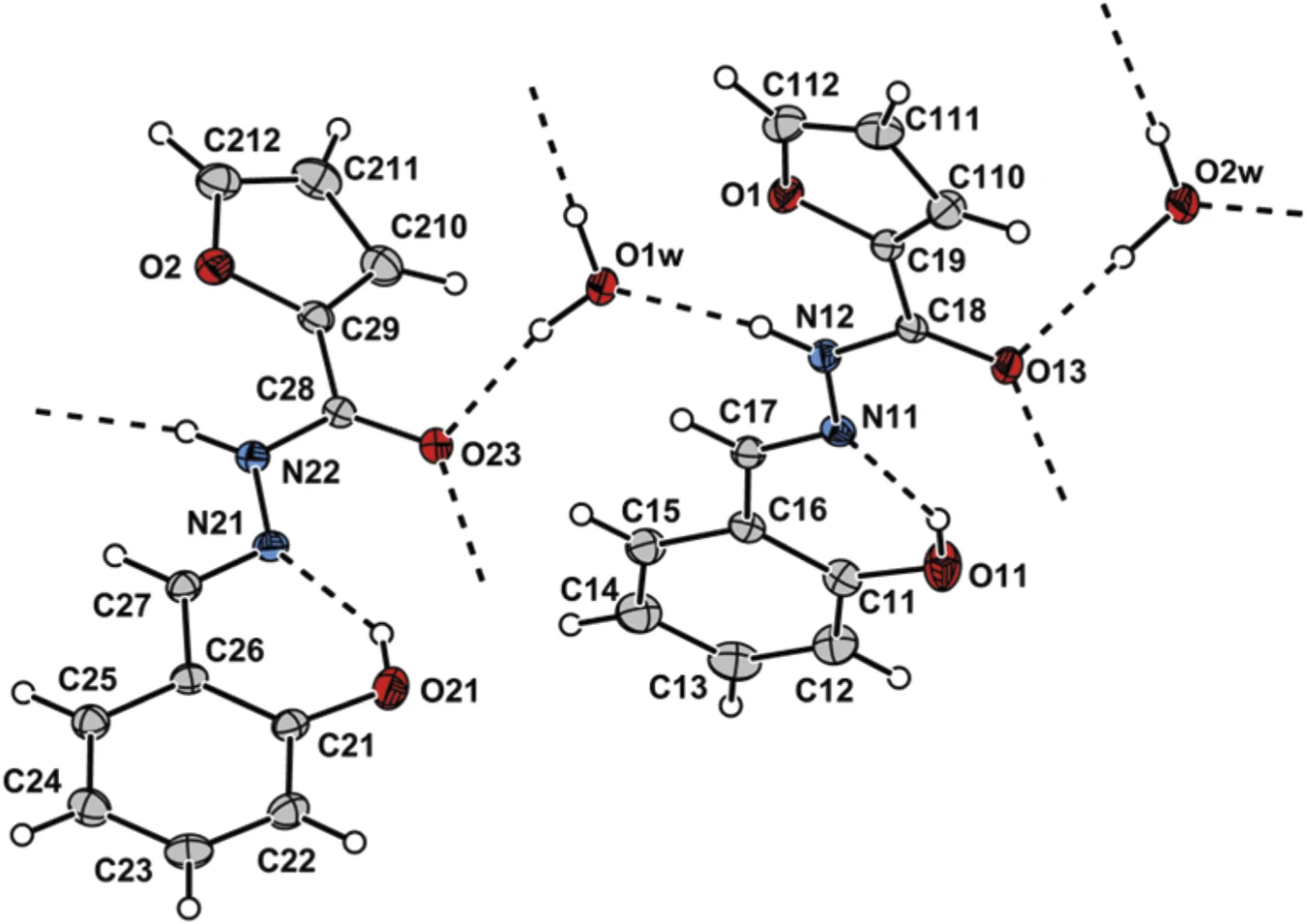
View of the two independent molecules in the solid-state L1·H_2_O compound showing the labeling of the non-H atoms and their displacement ellipsoids at the 30% probability level. Intra- and selected intermolecular H-bonds are indicated by dashed lines.

L2·H_2_O. A view of the crystal asymmetric unit content is shown in Figure 5 and its metrics are detailed in Supplementary Table S3. It is a monohydrate conformer of the molecule reported by Tang (2008), differing mainly by a 180° rotation of the methoxy -OCH_3_ group. In turn, L2 differs from L1 by the replacement of the furan cycle with the bulkier methoxybenzene group. Here, the above-mentioned tendency toward planarity due to π-bond delocalization is hampered by steric hindrance, and the methoxybenzene plane subtends a dihedral angle of 37.0 (1)° with the rest of the backbone skeleton. As for L1, the planarity of this molecular fragment is further favored by an intra-molecular OH … N bond [d (O … N) = 2.648 (6) Å and ∠(O-H … N) = 145 (10)°]. Neighboring molecules are bridged by the crystallization water molecule, acting as a donor in O_w_H … O (carb) bonds [O_w_H … O (carb) distances of 2.843 (5) and 2.843 (5) Å, and corresponding O_w_-H … O (carb) angles of 175 (8) and 157 (6)°] and as acceptor in NH … O_w_ interactions [d (N … O_w_) = 2.832 (5) Å and ∠(N-H … O_w_) = 168 (6)°]. H-bonds are further detailed in Supplementary Table S4.

**FIGURE 5:**
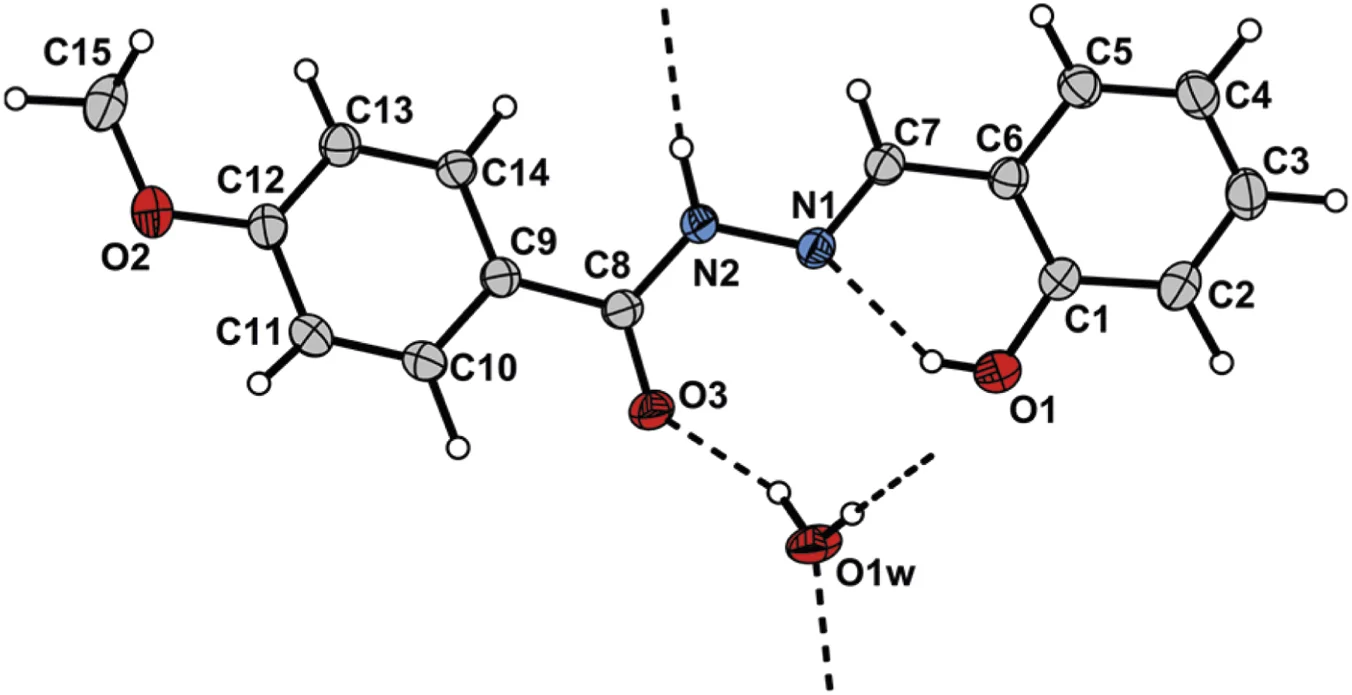
View of the solid-state L2.H_2_O compound showing the labeling of the non-H atoms and their displacement ellipsoids at the 30% probability level. Intra- and selected intermolecular H-bonds are indicated by dashed lines.

CuL1·H_2_O. The copper complex is shown in Figure 6, and bond distances and angles are in Supplementary Table S5. The Cu(II) ion is in a distorted square environment, coordinated to a L1^-^ anion acting as a tridentate ligand through his hydroxyl oxygen atom [d (Cu-O_hyd_) = 1.884 (3) Å], the carbonyl oxygen [d (Cu-O_carb_) = 1.971 (3) Å], and the N-atom [d (Cu-N) = 1.929 (3) Å] and a nitrate oxygen atom [d (Cu-O) = 2.002 (3) Å], all along electron lone-pair orbital lobes of the ligand atoms. The copper ion is capped by a slanted and much weaker Cu … O interaction with another nitrate oxygen atom [d (Cu … O) = 2.466 (3) Å]. *Trans* L1-Cu-L1 bond angles in the coordination plane are ∠(O_carb_-Cu-O_hyd_) = 173.9 (1)° and ∠(N-Cu-O_nitr_) = 171.2 (1)° and *cis* L1-Cu-L1 angles are in the 81.9(1)–93.1(1)° range. The crystallographic metrics around the metal indicate the copper d (x^2^-y^2^) orbital as the ground-state orbital for the unpaired electron (or hole).

**FIGURE 6:**
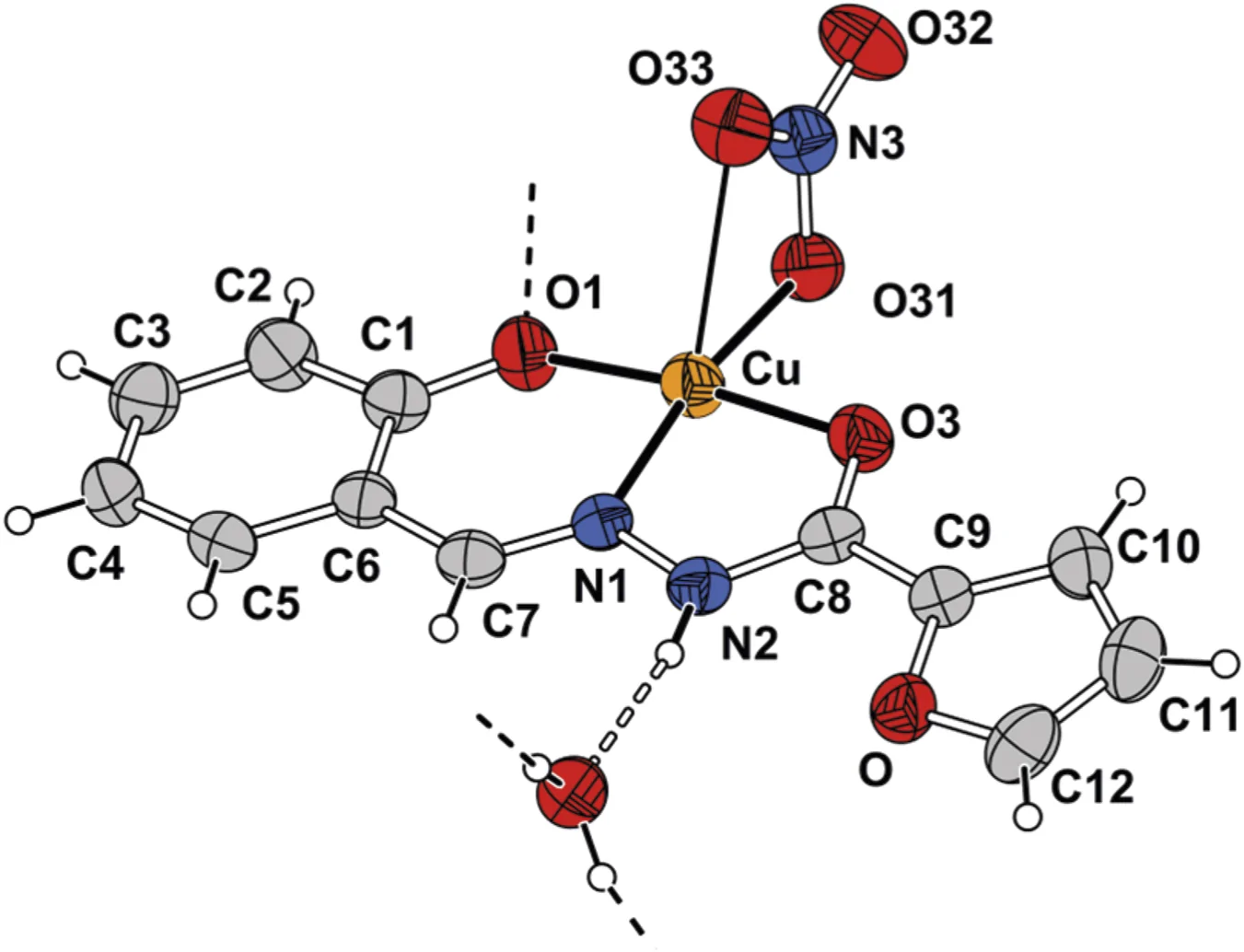
View of CuL1.H_2_O shows the labeling of the non-H atoms and their displacement ellipsoids at the 30% probability level. Strong copper–ligand coordination bonds are indicated by solid lines, weaker coordination bonds by thin lines, and H-bonds by dashed lines.

As expected, the major bond changes of the ligand L1^-^ upon hydroxyl deprotonation of L1 and coordination to the metal occur at the hydroxyl and carbonyl groups. >C (ph)-O bond length shortens from 1.353 (4) Å in unbonded L1 to 1.318 (4) Å in coordinated-to-metal L1^-^ (−0.035 Å = 9 times the standard deviation error σ) and to a much less degree > C=O bond distance lengthens from 1.240 (3) to 1.251 (4) Å (+0.011 Å = 3σ).

The crystallization water molecule links neighboring complexes in the lattice, as H-acceptor in a NH … O_w_ bond [d (H … O_w_) = 1.85 (4) Å and ∠(N-H … O_w_) = 168 (4)°] and as H-donor in O_w_H … O_hyd_ and O_w_H … O_nitr_ interactions [d (H … O_hyd_) = 1.93 (2) Å, ∠(O_w_-H … O_hyd_) = 176 (7)° and d (H … O_nitr_) = 2.07 (2) Å, ∠(O_w_-H … O_nitr_) = 164 (5)°, respectively]. H-bonds are further detailed in Supplementary Table S6.

Cu_2_L2_2_. The substance crystallizes as a centrosymmetric binuclear Cu(II) complex (Figure 7), and bond distances and angles are in Supplementary Table S7. The inversion-related Cu(II) ions are in a distorted square bipyramidal environment; each equatorially coordinated to a L2^-^ anion acting as a tridentate ligand through his hydroxyl oxygen atom [d (Cu-O_hyd_) = 1.960 (1) Å], the carbonyl oxygen [d (Cu-O_carb_) = 1.999 (1) Å], and the N-atom [d (Cu-N) = 1.938 (2) Å]. The fourth ligand is the hydroxyl oxygen atom of a neighboring L2^-^ ligand which bridges de metals in the binuclear complex [d (Cu-O_hyd_’) = 1.970 (1) Å, ∠(Cu-O_hyd_’-Cu’) = 99.74 (6)°, d (Cu … Cu’) = 3.0052 (5) Å], all nearly along electron lone-pair orbital lobes of the ligand atoms. The six-fold coordination around copper is completed by a nitrate oxygen atom at one pyramid axis [d (Cu-O_nitr_) = 2.637 (3) Å] and a solvent ethanol molecule occupies the opposite apical site at 2.371 (2) Å from the metal, hence completing a strongly elongated square bipyramidal coordination around the copper ions. *Trans* L-Cu-L bond angles in the coordination plane are ∠(O_carb_-Cu-O_hyd_) = 171.08 (6)° and ∠(N-Cu-O_hydr_’) = 171.75 (7)° and *cis* L-Cu-L angles are in the 80.26(6)-107.71(6)° range. The *Trans* L-Cu-L bond angle involving the axial ligands is 164.12 (7)°. Full bond distances and angles are provided in Supplementary Table S7. The crystallographic metrics indicate a copper *d* (*x* ^2^-*y* ^2^) ground state and, according to Anderson–Goodenough–Kanamori rules, the copper binding structure favors an AF exchange coupling between the unpaired electrons of neighboring copper (II) ions (Anderson, 1950; Goodenough, 1955; Goodenough, 1958; Kanamori, 1959).

**FIGURE 7:**
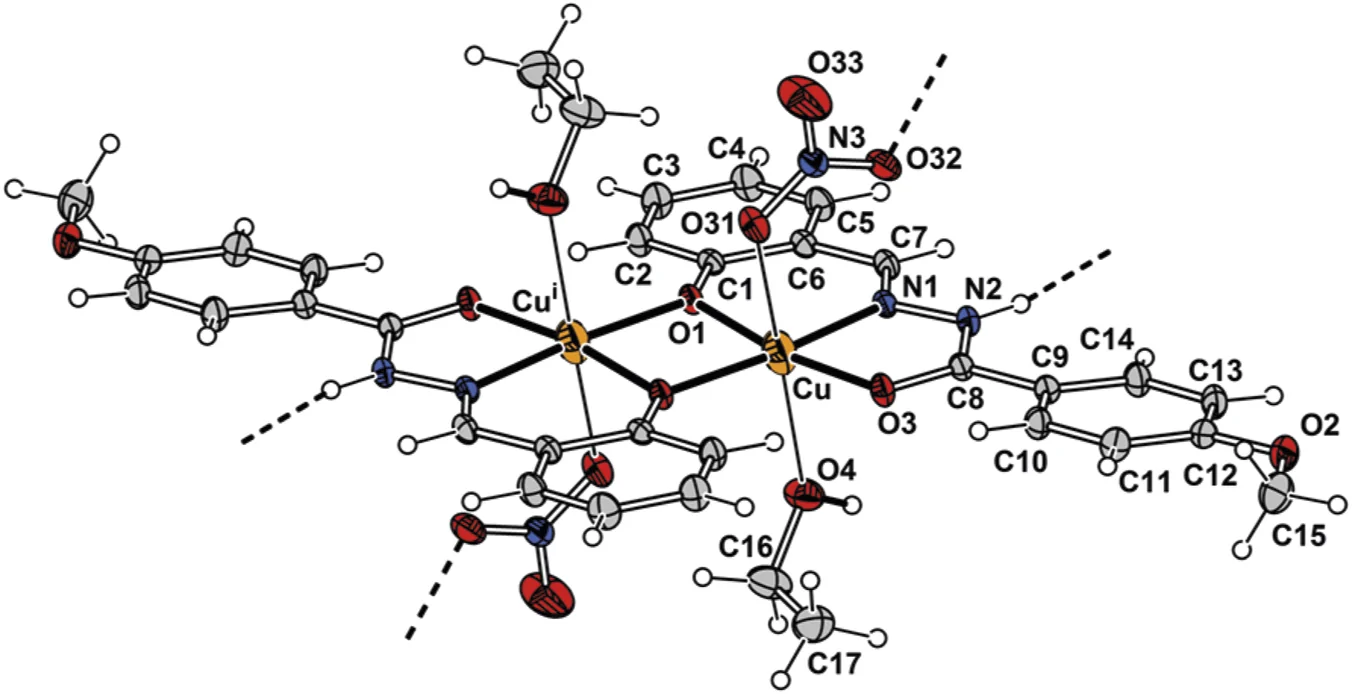
View of the centrosymmetric Cu_2_L2_2_ binuclear complex showing the labeling of the non-H atoms and their displacement ellipsoids at the 30% probability level. The unlabeled half of the complex is obtained from the labeled half through the inversion symmetry operation -x+1, -y+2, -z. Interdimeric NH … O_nitr_ bonds are indicated with dashed lines.

Neighboring binuclear units in Cu_2_L2_2_ are linked through a single NH … O_nitr_ bond [d (N … O_nitr_) = 2.769 (2) Å and ∠(N-H … O_nitr_) = 165°]. Further details of this bond along with other much weaker inter-dimeric EtOH … O_nitr_ and CH … O interactions are provided in Supplementary Table S8.

### 3.2 Characterization of L1, L2, and complexes CuL1 and Cu_2_L2_2_

The synthesized ligands and complexes were characterized by elemental analysis, ^1^H and ^13^C NMR, FT-IR, electronic spectra, and high-resolution mass spectrometry (HRMS). Elemental analysis of carbon, hydrogen, and nitrogen showed percentage values in agreement with those calculated for the proposed molecular formulas (see Materials and Methods section). FT-IR spectra of the solid ligands and their corresponding complexes were recorded in the 4000–400 cm^-1^ frequency range (Supplementary Figures S1 and S2). The vibrational assignments were based on the reported data (Balsa et al., 2021; Rodríguez et al., 2021; Santa Maria de la Parra et al., 2024b) and are summarized in Table 3. The compounds L1, L2, and CuL1 crystallize with one lattice water molecule, as we previously determined by X-ray diffraction. The bands assigned to the antisymmetric and symmetric stretching vibrations of hydrogen-bonded water molecules were observed between 3,550 and 3,300 cm^-1^ in the three spectra. The vibrational modes associated with the NH group remain largely unaffected in the complexes’ spectra compared to those observed for the free ligands, indicating retention of this proton upon coordination, which is also consistent with the results obtained from X-ray diffraction. The bands observed at 3,001, 1,369, and 848 cm^-1^ in the spectrum of L1 and 2999, 1375, and 849 cm^-1^ for L2, assigned to the OH group in the ligands, are absent in the spectra of the corresponding complexes. This finding confirms the deprotonation of the phenolic oxygen, leading to the formation of a phenoxide group coordinated to the Cu(II) center. The bands observed at 528 and 556 cm^-1^ in the spectra of CuL1 and Cu_2_L2_2_, respectively, indicate coordination of the metal center to this oxygen atom. Furthermore, the bands assigned to νC = O of the amide moiety (1,642 and 1,623 cm^-1^) and imine group (1,609 and 1,605 cm^-1^) in the L1 and L2 spectra, respectively, are overlapped and shifted to lower frequencies in the complex spectra, thus confirming the coordination to the metal center through these groups. These findings indicate tridentate coordination of ligands L1 and L2 to the metal center through the imine *N*-atom, the O-atom from the carbonyl group, and the O-atom from the phenolic group.

**TABLE 3:** Assignment of the most significant vibrational modes for ligand L1 and L2 and complexes CuL1 and Cu_2_L2_2_ acquired experimentally.

L1		CuL1	
Frequency (cm^-1^)	Assignment	Frequency (cm^-1^)	Assignment
3,451^sh^	${\left[\nu_{\text{as}}\text{OH}\right]}^{H_{2}O\;crist}$	3,406^m-w,b^	${\left[\nu_{\text{as}}\text{OH}\right]}^{H_{2}O\;crist}$
3,409^s,b^	${\left[\nu_{s}\text{OH}\right]}^{H_{2}O\;crist}$	3,332^m-w,b^	${\left[\nu_{s}\text{OH}\right]}^{H_{2}O\;crist}$
3,201^m,b^	νNH	3,195^vw,b^	νNH
3,001^m^	νOH	​	​
1,642^vs^ 1,624^vs^	${\left[\nu C=O\right]}^{2FH}$ ${\left[\delta \text{NH}\right]}^{2FH}$	1,629s	${\left[\nu C=O+\delta \text{NH}\right]}^{2FH}$
1,609^vs^	νC=N	1,601^m-s^	νC=N
​	​	1,384^vs^	${\nu_{as}NO}_{3}$
1369^m-s^	$\delta \text{OH}+{\left[\nu^{R}\right]}^{ph}$	​	​
1,158^m-w^	νN−N	1,161^w^	νN−N
​	​	1,051^w^	${\nu_{s}NO}_{3}$
848^m^	γOH	​	​
​	​	528^w^	νCu−O
L2		Cu_2_L2_2_	
3,531^m,b^	${\left[\nu_{\text{as}}\text{OH}\right]}^{H_{2}O\;crist}$	​	​
3,353^m,b^	${\left[\nu_{s}\text{OH}\right]}^{H_{2}O\;crist}$	​	​
3,212^m,b^	νNH	3,188^m,b^	νNH
2,999^m-s^	νOH	​	​
1,623^vs^ 1,605^vs^	${\left[\nu C=O\right]}^{4MBH}$ νC=N	1,603^vs^	νC=O+νC=N
​	​	1,386^vs^	${\nu_{as}NO}_{3}$
1,375^m-s^	$\delta \text{OH}+{\left[\nu^{R}\right]}^{ph}$	​	​
1,151^m-w^	νN−N	1,167^m-w^	νN−N
​	​	1,046^w^	${\nu_{s}NO}_{3}$
849^m^	γOH	​	​
617^w^	γNH	607^w^	γNH
​	​	556^w^	νCu−O

References: ph, phenyl ring; νas, antisymmetric stretching mode; νs, symmetric stretching mode; δ, bending mode; γ, out-of-plane bending mode. Intensities of the experimental bands vs, very strong; s, strong; m, medium; m-s, medium–strong; m-w, medium–weak; w, weak; b, broad; sh, shoulder.

High-resolution mass spectrometry (HRMS) further confirmed the composition of the compounds in solution. For the free ligands, the spectra recorded in positive ionization mode cleanly displayed the characteristic protonated molecular ions [L + H]^+^ (Supplementary Figures S3, 4). Conversely, the mononuclear copper complex CuL1 exhibited significantly better ionization efficiency and resolution in negative mode, where it was detected as a deprotonated species (Supplementary Figure S5). In the case of the dimeric complex Cu_2_L2_2_, ESI + -HRMS analysis revealed the predominant presence of the dicationic core [Cu_2_(µ-L2-H)_2_]^2+^ (Supplementary Figure S4). These results indicate that nitrate anions and coordinated ethanol molecules, although explicitly observed in the solid-state crystal structure, readily dissociate in methanol solution during electrospray ionization.

The ^1^H and ^13^C NMR spectra of the free ligands (L1 and L2) were recorded in DMSO-d_6_ solution, and two-dimensional NMR experiments (^1^H–^1^H COSY, ^1^H–^13^C HSQC, and ^1^H–^13^C HMBC) were employed to achieve complete signal assignment. The ligand atom numbering scheme used for the ^1^H and ^13^C NMR discussion is depicted in Supplementary Figure S7. The chemical shifts, signal multiplicities, and integrations are consistent with the proposed structures of the ligands, confirming the integrity of the hydrazone framework (Supplementary Figures S8-S17). The complete assignment including chemical shifts (δ), multiplicities, and coupling constants (J) for all studied compounds are presented after the description of the synthesis. ^1^H NMR signal integration and signal multiplicities agree with the proposed molecular formula. Both free ligands displayed similar features to the structurally related compounds previously reported by us (Santa Maria de la Parra et al., 2026). Signals corresponding to the aromatic protons were clearly detected in the 7.0–8.7 ppm region, and the key singlet signal corresponding to H8 was identified in both molecules. Furthermore, heteroatom-labile protons (H7 and H10) appeared upfield without any coupling with another proton or carbon. The signal corresponding to the methyl group in L2 was identified at 3.85 ppm. The ^13^C NMR signals were assigned using the bidimensional spectra. All ^13^C were identified with exception of quaternary carbon in L1 since they do not appear in the spectra (Supplementary Figure S9). Overall, signal integrals and multiplicities matched the stoichiometry derived from the synthetic procedure and elemental analysis, further supporting structural consistency.

Electronic absorption spectra in the UV−VIS region of the CuL1 and Cu_2_L2_2_ complexes, as well as their corresponding ligands, were recorded between 280 and 900 nm both in the solid state and DMSO solution at different concentrations, to identify transitions with different molar absorptivities.

Considering the square pyramid geometry of the coordination environment observed for CuL1 by crystallography, absorption bands assignable to *d–d* electronic transitions are expected to be observed in the visible region of the spectrum. As shown in Figure 8a, this band was observed at 694 nm, consistent with the green color of the solution and characteristic of *d–d* electronic transitions of Cu(II). The band centered at 392 nm, with a shoulder at 408 nm, is attributed to ligand-to-metal charge transfer transitions (LMCTs) between σO and dCu orbitals (Burgos-Lopez et al., 2019). The set of bands observed at 303, 317, and 332 nm, assigned to intraligand transitions (ILCTs), appears at longer wavelengths than those corresponding to L1 (291, 302, and 331), which is consistent with the decrease in their energy upon complex formation. The band assignments were based on results previously reported for similar compounds (Rodríguez et al., 2021; Santa Maria de la Parra et al., 2024b).

**FIGURE 8:**
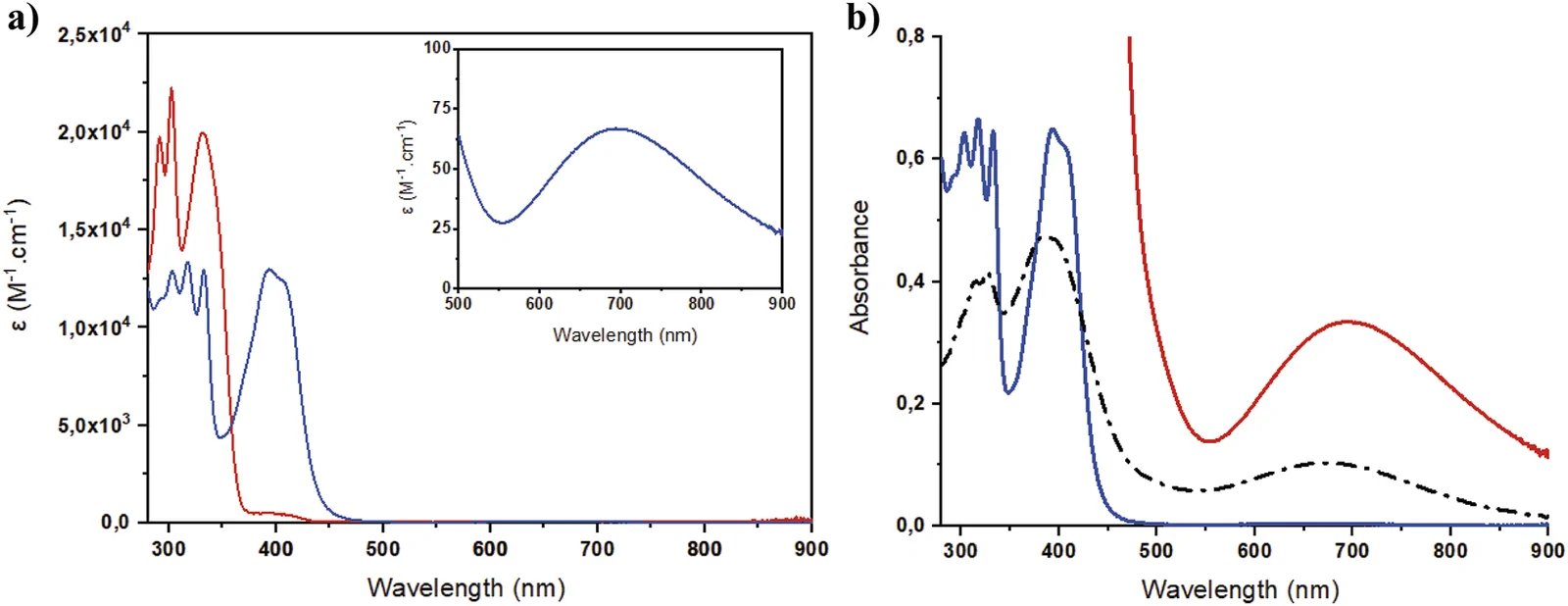
**(a)** UV–visible spectra of CuL1 (blue) in DMSO at concentrations of 5 × 10^−5^ M and 5 × 10^−3^ M (inset), compared with that obtained for the ligand L1 at 2.5 × 10^−5^ M (red). **(b)** UV–visible spectra of CuL1 in DMSO at concentrations of 5 × 10^−5^ M (blue) and 5 × 10^−3^ M (red), compared with that obtained for the complex in the solid state (black, dashed).

Furthermore, comparison with the spectrum obtained for the solid sample reveals the same bands as those observed in solution, with only minor differences attributable to the distinct measurement techniques (Figure 8b). The experimental electronic spectra, together with the proposed assignments, are detailed in Table 4.

**TABLE 4:** Experimental electronic spectra of the complexes CuL1 and Cu_2_L2_2_ in DMSO solution and the solid state. Band maxima are expressed in nm. Molar absorptivity (in M^-1^cm^-1^) is indicated in parentheses. The proposed assignment is also included.

Compound	Experimental in DMSO (ε, M^-1^cm^-1^)	Experimental, solid sample	Assignment
CuL1	694 (67)	676	*d* → *d*
	408 (shoulder) 393 (1,3 × 10^4^)	395 (shoulder) 384	LMCT
	332 (1,3 × 10^4^) 317 (1,3 × 10^4^) 303 (1,3 × 10^4^)	326 314	ILCT
Cu_2_L2_2_	708 (226)	703	*d* → *d*
	403 (shoulder) 392 (3,6 × 10^4^)	392 (shoulder) 371	LMCT
	332 (3,3 × 10^4^) 317 (4,2 × 10^4^) 303 (4,1 × 10^4^)	330 (shoulder) 321	ILCT

Similarly, the spectra obtained for Cu_2_L2_2_ and L2 in the solid state and in DMSO solution were analyzed (see Figure 9) and compared with the spectra obtained for similar compounds (Balsa et al., 2021; Ferraresi-Curotto et al., 2015). The band corresponding to the *d–d* transition of the Cu(II) ion appears at 708 nm in the spectrum obtained for the 2.5 × 10^−5^ M solution of Cu_2_L2_2_ and at 703 nm in the spectrum of the solid sample. The band appearing with a maximum at 392 nm in the solution spectrum and at 371 nm in the solid sample was assigned to ligand-to-metal charge transfer. Finally, the set of bands between 300 and 335 nm in both spectra of the complex is attributed to intraligand processes, consistent with those observed in the spectrum of L2.

**FIGURE 9:**
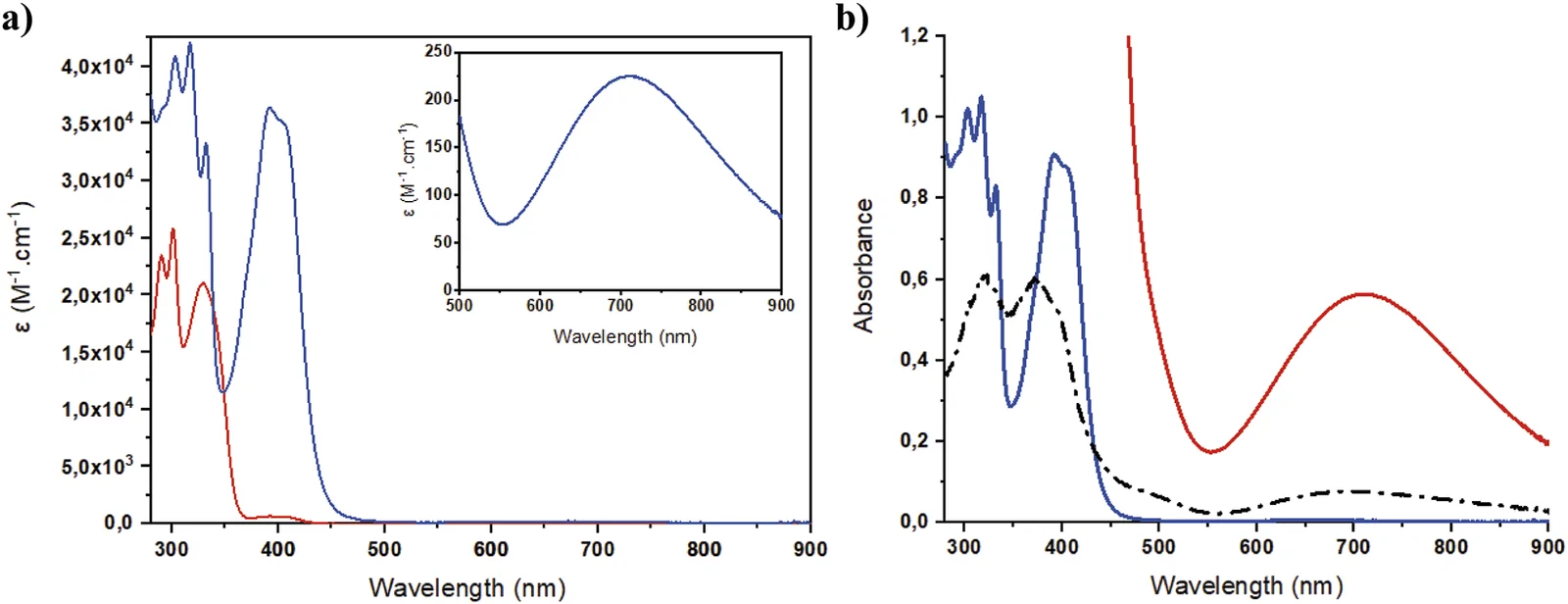
**(a)** UV–visible spectra of Cu_2_L2_2_ (blue) in DMSO at concentrations of 2.5 × 10^−5^ M and 2.5 × 10^−3^ M (inset), compared with that obtained for the ligand L2 at 2.5 × 10^−5^ M (red). **(b)** UV–visible spectra of Cu_2_L2_2_ in DMSO at concentrations of 2.5 × 10^−5^ M (blue) and 2.5 × 10^−3^ M (red), compared with that obtained for the complex in the solid state (black, dashed).

Furthermore, stability studies performed over 24 h (Supplementary Figures S18, 19) demonstrated that both CuL1 and Cu_2_L_2_ remained stable in DMSO. In PBS, CuL1 also remained stable, whereas Cu_2_L_2_ exhibited a gradual decrease in signal intensity over time. This decrease is attributed to its lower solubility in aqueous media at this concentration, rather than to chemical degradation as no shift in the characteristic absorption bands was observed throughout the experiment. Although Cu_2_L_2_ displays lower aqueous solubility, the solutions used for the biological assays were freshly prepared immediately before use, thereby minimizing any potential impact of this limitation. Moreover, because cell culture medium is substantially more complex than PBS and cannot be accurately reproduced by this simplified system, both complexes were considered sufficiently stable for subsequent *in vitro* biological studies.

### 3.3 2D studies

#### 3.3.1 Cell viability

Cytotoxicity studies for ligands (L1 and L2) and their copper (II) complexes (CuL1 and Cu_2_L2_2_) on the human OS MG-63 cell line were performed using the MTT assay. Supplementary Figure S20 shows that both complexes exhibited anticancer activity against MG-63 cells at low-micromolar concentrations from 0.5 to 3 µM (*p* < 0.0001) in a dose-dependent manner with IC_50_ values of 2.3 ± 0.1 and 0.9 ± 0.1 µM for CuL1 and Cu_2_L2_2_, respectively (Table 5). Moreover, both complexes exhibited stronger anticancer activity than CDDP (IC_50,CDDP_ = 39 ± 1.8 µM). However, both ligands and Cu(NO_3_)_2_ did not affect the cell viability until 100 μM, highlighting the significance of metal coordination to enhance the anticancer activity of the inactive ligands, as we previously reported (Espindola-Moreno et al., 2025; Rodríguez et al., 2021; Santa Maria de la Parra et al., 2026a; Santa Maria de la Parra et al., 2026b; Santa Maria de la Parra et al., 2024b; Santa Maria de la Parra et al., 2026).

**TABLE 5:** IC_50_ (µM) values of L1, L2, CuL1, Cu_2_L2_2_, and structurally related Cu(II) complexes in MG-63 and L929 cells (2D) after 24 h of incubation. n.d, not determined.

Compound	MG-63	L929	SI	Reference
L1	>100	>100	-	This work
L2	>100	>100	-	This work
CuL1	2.3 ± 0.1	3.4 ± 0.1	1.5	This work
Cu_2_L2_2_	0.9 ± 0.1	0.8 ± 0.1	0.9	This work
CuL3	1.1 ± 0.1	2.5 ± 0.1	2.3	Santa Maria de la Parra et al. (2024b)
CuL4	>25	>25	-	Santa Maria de la Parra et al. (2026)
CuL5	2.0 ± 0.5	3.6 ± 0.3	1.8	Rodríguez et al. (2021)
Cu_4_(L6)_4_	0.50 ± 0.04	1.04 ± 0.04	2.1	Santa Maria de la Parra et al. (2026a)
CuL7	2.6 ± 0.3	4.2 ± 0.1	1.6	Balsa et al. (2021), Balsa et al. (2024)
CuL8	8.8 ± 0.3	-	-	Burgos-Lopez et al. (2019)
CuL8 (o-phen)	3.5 ± 0.3	-	-	Burgos-López et al. (2022)
CuL8 (bipy)	5.6 ± 1.0	-	-	Burgos-López et al. (2022)
CuL9	24.7 ± 2.2	52.6 ± 4.2	2.1	Santa Maria de la Parra et al. (2026)
CDDP	39.0 ± 1.8	11.2 ± 1.6	0.3	Ruiz et al. (2019)

Several scientific reports indicate that copper complexes with IC_50_ values lower than 10 µM are efficient cytotoxic agents against several human cancer cell lines (Denoyer et al., 2018; Santini et al., 2014). As discussed above, our group reported a series of Cu(II)-hydrazone complexes with potent cytotoxic activity against MG-63 cells (see Figure 1; Table 5). The complexes CuL3 and CuL7 are structurally related to CuL1 and Cu_2_L2_2_, respectively, via the hydrazide moiety of the ligand (furan and methoxy-benzohydrazide), as shown in Figure 1. However, the structural difference in the aldehyde group of the ligand (2-hydroxy-3-methoxybenzaldehyde > 2-hydroxy-benzaldehyde) is reflected in the lower cytotoxic potency of CuL1 compared to CuL3. In contrast, Cu_2_L2_2_ exhibits enhanced anticancer activity relative to CuL7, as evidenced by an almost 3-fold reduction in the IC_50_ value. This difference may be attributed to the fact that Cu_2_L2_2_ is a dimer, whereas CuL7 is a monomeric complex; notably, increased nuclearity has previously been associated with enhanced cytotoxic activity, as earlier demonstrated for Cu_4_(L6)_4_ (Santa Maria de la Parra et al., 2026a).

We also evaluated the effect of ligands and complexes on the viability of non-tumor cells, such as the L929 mouse fibroblast line (see Table 5; Supplementary Figure S21). The selectivity index (SI = IC_50_ non-tumoral cells/IC_50_ tumor cells) of both complexes exceeded that of CDDP (SI = 0.3), with CuL1 exhibiting the greatest selectivity. Given these promising results, we continued the study of the cytotoxic activity of both complexes.

#### 3.3.2 Oxidative stress

Numerous studies have demonstrated a correlation between redox potential and the anticancer activity of copper compounds, which is largely attributed to the accumulation of reactive oxygen species (ROS) generated through Fenton-like reactions induced by these complexes (Garcia‐Bosch and Siegler, 2016; Jungwirth et al., 2011). In this context, the DHR-123 probe, which selectively reacts with hydrogen peroxide and peroxynitrite, was employed to evaluate the potential effect of the complexes on ROS formation (Kooy et al., 1994; Qin et al., 2008). Following cellular uptake, intracellular ROS oxidize DHR-123 to fluorescent rhodamine 123. The incubation of MG-63 cells with CuL1 for 3 h caused a marked increase in intracellular ROS levels from 340% to 450% over basal in the range of 5–20 μM, as shown in Figure 10. Similarly, incubation with Cu_2_L2_2_ triggered an increase in ROS production from 1 µM (215%) to 20 µM (515%) over basal (*p* < 0.0001).

**FIGURE 10:**
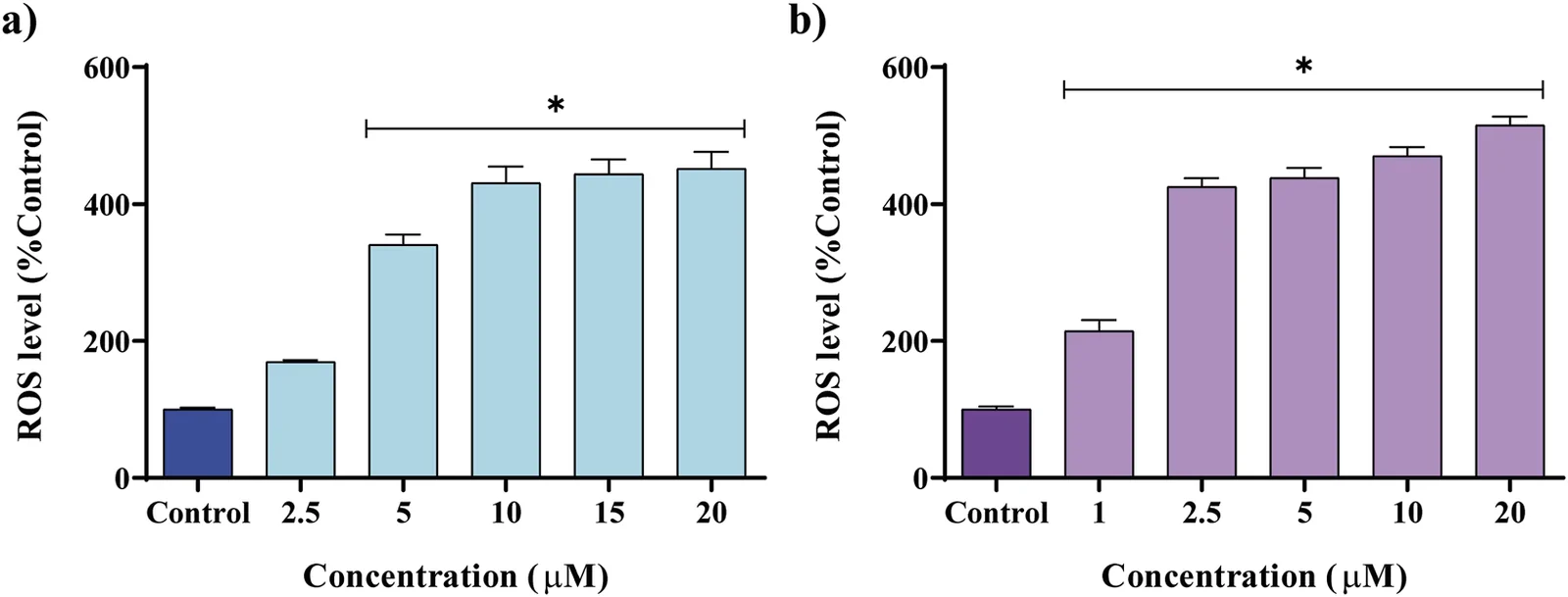
Generation of intracellular ROS in MG-63 cells after 3 h treatment with **(a)** CuL1 or **(b)** Cu_2_L2_2_. The results of fluorescence were corrected by their protein content and are expressed as the % of basal level (Control) as the mean ± SEM (n = 9). *p < 0.0001 differences between Control and treatment.

Our results demonstrate that both complexes are internalized by MG-63 cells and induce ROS production within as little as 3 h, indicating that ROS generation may be a key component of their mechanism of action. Evidence indicates that cancer cells experience oxidative stress that supports proliferation and other processes essential for tumor development (Schumacker, 2006; Valko et al., 2006). Consequently, basal ROS levels are typically higher in cancer cells than in normal cells, rendering them more susceptible to chemotherapeutic and redox-active agents—such as copper (II) complexes—that further enhance ROS production.

### 3.4 3D studies

Although 2D cultures are the first step in assessing a compound’s cytotoxicity, 3D cultures offer a more accurate representation of the intratumoral environment, including gradients of nutrients and oxygen, as well as cell–cell interactions (Kondo and Inoue, 2019). Multicellular resistance (MCR), a hallmark of solid tumors, can also be reproduced using 3D models, which reduce cellular sensitivity to anticancer therapies and result from cell–cell interaction, limited drug penetration, and the resistance of quiescent cells found in the MCS core (Mitrakas et al., 2023). Due to its impact on apoptotic factor expression, hypoxia has also been implicated as a cause of MCR (Erler et al., 2004).

#### 3.4.1 Spheroid cell viability studies

To further investigate the antitumor activity of CuL1 and Cu_2_L2_2_, we generated MCS from MG-63 cells by the hanging drop technique (León et al., 2016). Our results indicated that complexes CuL1 and Cu_2_L2_2_ impaired the cell viability of spheroids derived from MG-63 cells showing IC_50_ of 25.0 ± 0.4 and 14.7 ± 0.9 µM, respectively, corresponding to cytotoxic activities approximately 2.5-fold and 4.5-fold higher than CDDP (IC_50_ = 65.1 ± 5.6 µM). As shown in Table 6, comparison of the potency of the CuL1 and Cu_2_L2_2_ complexes with that of the CuL3 and CuL7 complexes indicates that the removal of the methoxy group from the benzaldehyde moiety diminished the cytotoxicity of the complexes. In addition, the complexes exerted dose-dependent effects on the spheroids’ volume and shape, as shown in Figure 11.

**TABLE 6:** IC_50_ (µM) values of CuL1, Cu_2_L2_2_, and structurally related Cu(II) complexes in MG-63 cells (2D) and spheroids derived from MG-63 cells (3D) after 24 h of incubation. n.d, not determined.

Compound	2D	3D	Reference
CuL1	2.3 ± 0.1	25.0 ± 0.4	This work
Cu_2_L2_2_	0.9 ± 0.1	14.7 ± 0.9	This work
CuL3	1.1 ± 0.1	16.3 ± 3.1	Santa Maria de la Parra et al. (2024b)
CuL5	2.1 ± 0.3	9.1 ± 1.0	Balsa et al. (2023d)
Cu_4_(L6)_4_	0.50 ± 0.04	4.1 ± 0.1	Santa Maria de la Parra et al. (2026a)
CuL7	2.6 ± 0.3	9.9 ± 1.4	Balsa et al. (2024)
CDDP	39.0 ± 1.8	65.1 ± 5.6	Ruiz et al. (2019)

**FIGURE 11:**
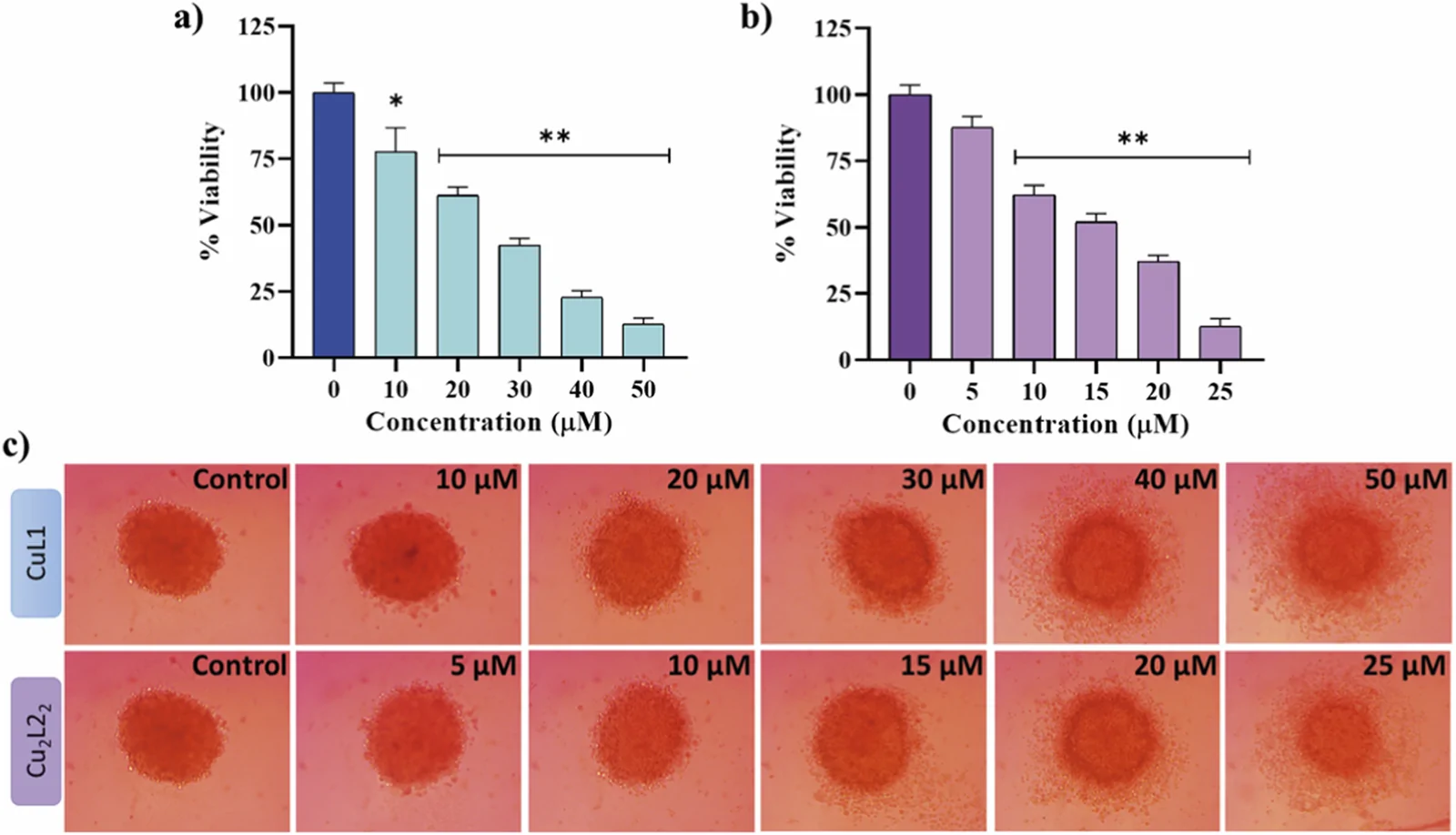
Cell viability assay of the MG-63 spheroids treated for 24 h with DMEM (Control) or different concentrations of **(a)** CuL1 and **(b)** Cu_2_L2_2_. **(c)** Representative images of the multicellular spheroids treated with different concentrations of CuL1 and Cu_2_L2_2_ for 24 h *p < 0.01 and **p < 0.0001 differences between Control and treatment.

#### 3.4.2 MCS spreading assay

Metastasis represents the leading cause of cancer-related mortality and involves the dissemination of malignant cells from the primary tumor to adjacent tissues and distant organs (Fares et al., 2020). This multistep process is driven by several factors, including cellular migration through blood and lymphatic vessels, invasive growth across the extracellular matrix (ECM), and the establishment of secondary tumor colonies at distant sites (Kramer et al., 2013). In this context, spheroid spreading assays provide a valuable *in vitro* model for mimicking the migration of cells from small tumor aggregates and investigating processes associated with tumor dissemination. To investigate the effect of Cu(II) complexes on the migration of spheroid cells, MCS derived from MG-63 cells were treated with CuL1 at concentrations ranging from 2.5 to 10 µM and Cu_2_L2_2_ at concentrations ranging from 1 to 5 µM (see Supplementary Figure S22). Using the migration area of untreated spheroids as 100% cell migration, treatment of the spheroids with the CuL1 complex reduced cell migration to 60% at 5 µM and further to 20% at 10 µM (see Figure 12). A similar reduction in cell migration was observed following treatment with the Cu_2_L2_2_ complex at concentrations of 2.5 and 5 µM. Importantly, this effect was observed even at sub-IC_50_ concentrations, a phenomenon we reported previously for CuL3 and Cu_4_(L6)_4_ complexes on MG-63-derived MCS (Santa Maria de la Parra et al., 2026a; Santa Maria de la Parra et al., 2024b). These findings further support the inhibitory effect of the CuL1 and Cu_2_L2_2_ complexes on cell migration from OS spheroids.

**FIGURE 12:**
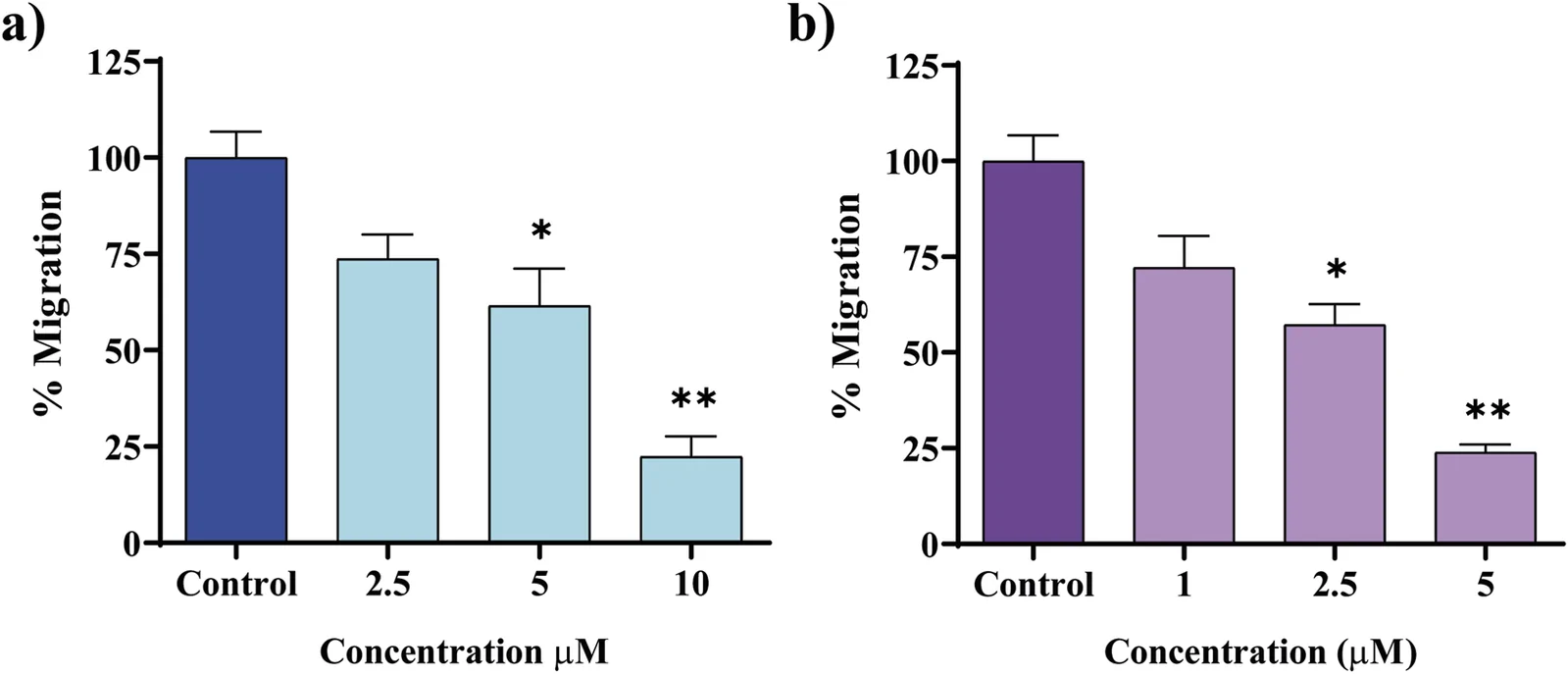
Spreading assay of spheroids. MCS were incubated 24 h with 0.5% DMSO in DMEM (Control) or different concentrations of **(a)** CuL1 or **(b)** Cu_2_L2_2_. The results are expressed as the % migration and represent the mean ± SEM. *p < 0.01 and **p < 0.0001 differences between control and treatment.

## 4 Discussion

In this study, two new Cu(II)-hydrazone complexes were successfully synthesized, structurally characterized, and evaluated for their antitumor activity against osteosarcoma using complementary 2D and 3D cellular models. Metal coordination markedly enhanced the biological activity of the hydrazone ligands, yielding compounds with potent antiproliferative effects that clearly outperformed cisplatin under the experimental conditions evaluated. Both complexes also promoted intracellular ROS generation and significantly impaired tumor cell migration, highlighting their ability to affect multiple processes associated with tumor progression.

Among the investigated compounds, the binuclear complex Cu_2_L2_2_ consistently exhibited superior biological performance, demonstrating greater cytotoxic potency than its mononuclear analog in both monolayer cultures and multicellular spheroids. The preservation of its activity in the more physiologically relevant 3D model further supports its therapeutic potential.

Overall, these findings reinforce the value of hydrazone-based Cu(II) complexes as promising candidates for osteosarcoma treatment and emphasize the importance of rational coordination design in the development of copper-based metallodrugs. Further mechanistic investigations and *in vivo* studies will be essential to establish their pharmacological profile and assess their potential for preclinical development.

## Data Availability

Crystal data and structure refinement results are summarized in Tables 1, 2. Crystallographic structural data have been deposited at the Cambridge Crystallographic Data Centre (CCDC). Any request to the Cambridge Crystallographic Data Centre for this material should quote the full literature citation and the reference numbers CCDC 2306862 (L1.H_2_O), 2392963 (CuL1.H_2_O), 2392965 (L2.H_2_O), and 2392966 (Cu2L2_2_).

## References

[B1] Anderson P. W. (1950). Antiferromagnetism. Theory of superexchange interaction. Phys. Rev. 79 (2), 350–356. 10.1103/PhysRev.79.350

[B2] Anoopkumar-Dukie S. Carey J. B. Conere T. O’Sullivan E. Van Pelt F. N. Allshire A. (2005). Resazurin assay of radiation response in cultured cells. Br. J. Radiology 78 (934), 945–947. 10.1259/bjr/54004230 16177019

[B3] Balsa L. M. Ferraresi-Curotto V. Lavecchia M. J. Echeverría G. A. Piro O. E. García-Tojal J. (2021). Anticancer activity of a new copper(II) complex with a hydrazone ligand. Structural and spectroscopic characterization, computational simulations and cell mechanistic studies on 2D and 3D breast cancer cell models. Dalton Trans. 50 (28), 9812–9826. 10.1039/d1dt00869b 34190268

[B4] Balsa L. M. Baran E. J. León I. E. (2023a). Copper Complexes as Antitumor Agents: *in vitro* and *in vivo* Evidence. Curr. Med. Chem. 30 (5), 510–557. 10.2174/0929867328666211117094550 34789122

[B5] Balsa L. M. Rodriguez M. R. Ferraresi-Curotto V. Parajón-Costa B. S. Gonzalez-Baró A. C. León I. E. (2023b). Finding new molecular targets of two Copper(II)-Hydrazone complexes on triple-negative breast cancer cells using mass-spectrometry-based quantitative proteomics. Int. J. Mol. Sci. 24 (8), 7531. 10.3390/ijms24087531 37108690 PMC10139133

[B6] Balsa L. M. Ferretti V. Sottile M. Nunes P. Costa Pessoa J. Correia I. (2023c). New copper(ii) and oxidovanadium(iv) complexes with a vitamin B6 Schiff base: mechanism of action and synergy studies on 2D and 3D human osteosarcoma cell models. Dalton Trans. 53 (7), 3039–3051. 10.1039/d3dt02964f 38111362

[B7] Balsa L. M. Solernó L. M. Rodriguez M. R. Parajón-Costa B. S. Gonzalez-Baró A. C. Alonso D. F. (2023d). Cu(II)-acylhydrazone complex, a potent and selective antitumor agent against human osteosarcoma: mechanism of action studies over *in vitro* and *in vivo* models. Chem. Biol. Interact. 384, 110685. 10.1016/j.cbi.2023.110685 37666443

[B8] Balsa L. Santa Maria de la Parra L. Ferretti V. León I. (2024). Deciphering the effect of a Cu(II)‐hydrazone complex on intracellular cell signalling pathways in a human osteosarcoma 2D and 3D models. ChemBioChem 25, e202400373. 10.1002/cbic.202400373 39121373

[B9] Balsa L. M. Santa Maria de la Parra L. Espindola-Moreno O. León I. E. (2025). Advances in copper complexes in cancer treatment. Drug Discov. Today 30 (12), 104522. 10.1016/j.drudis.2025.104522 41183662

[B10] Bikas R. Hosseini Monfared H. Kazak C. Arslan N. B. Bijanzad K. (2010). (E)-N′-(2-Hydroxybenzylidene)furan-2-carbohydrazide. Acta Crystallogr. Sect. E Struct. Rep. Online 66 (8), o2015. 10.1107/S1600536810024839 21588326 PMC3007379

[B11] Bray F. Laversanne M. Sung H. Ferlay J. Siegel R. L. Soerjomataram I. (2024). Global cancer statistics 2022: GLOBOCAN estimates of incidence and mortality worldwide for 36 cancers in 185 countries. CA Cancer J. Clin. 74 (3), 229–263. 10.3322/caac.21834 38572751

[B12] Brooks L. Palmeira-Mello M. V. Fernandez C. Y. Arandia I. Ellena J. Costa-Filho A. J. (2026). New Cu complexes containing methyl-phenanthroline and dipeptides as cytotoxic agents. Synthesis, characterization and *in vitro* studies. J. Inorg. Biochem. 274, 113089. 10.1016/j.jinorgbio.2025.113089 41043283

[B13] Burgos-Lopez Y. Del Plá J. Balsa L. M. León I. E. Echeverría G. A. Piro O. E. (2019). Synthesis, crystal structure and cytotoxicity assays of a copper(II) nitrate complex with a tridentate ONO acylhydrazone ligand. Spectroscopic and theoretical studies of the complex and its ligand. Inorganica Chim. Acta 487, 31–40. 10.1016/j.ica.2018.11.039

[B14] Burgos-López Y. Balsa L. M. Piro O. E. León I. E. García-Tojal J. Echeverría G. A. (2022). Tridentate acylhydrazone copper(II) complexes with heterocyclic bases as coligands. Synthesis, spectroscopic studies, crystal structure and cytotoxicity assays. Polyhedron. 213, 115621. 10.1016/j.poly.2021.115621

[B15] Cadavid-Vargas J. Leon I. Etcheverry S. Santi E. Torre M. Di Virgilio A. (2017). Copper(II) complexes with saccharinate and glutamine as Antitumor agents: Cyto- and genotoxicity in human osteosarcoma cells. Anticancer Agents Med. Chem. 17 (3), 424–433. 10.2174/1871520616666160513130204 27173967

[B16] Del Bello F. Pellei M. Bagnarelli L. Santini C. Giorgioni G. Piergentili A. (2022). Cu(I) and Cu(II) complexes based on Lonidamine-Conjugated ligands designed to promote synergistic Antitumor effects. Inorg. Chem. 61 (12), 4919–4937. 10.1021/acs.inorgchem.1c03658 35285628 PMC8965879

[B17] Denoyer D. Clatworthy S. A. S. Cater M. A. (2018). “Metallo-Drugs: Development and action of anticancer agents,”. Editors Sigel A. Sigel H. Freisinger E. Sigel R. K. O. (Berlin, Boston: De Gruyter), 18, 469. 10.1515/9783110470734

[B18] Erler J. T. Cawthorne C. J. Williams K. J. Koritzinsky M. Wouters B. G. Wilson C. (2004). Hypoxia-Mediated down-regulation of bid and bax in tumors occurs *via* hypoxia-inducible factor 1-Dependent and -Independent mechanisms and contributes to drug resistance. Mol. Cell Biol. 24 (7), 2875–2889. 10.1128/MCB.24.7.2875-2889.2004 15024076 PMC371100

[B19] Espindola-Moreno O. da Silva Moura F. Santa Maria de la Parra L. Stellet C. Serna J. D. P. Diniz R. (2025). Antiproliferative activity of a series of copper(II) complexes derived from a furan-containing N-acylhydrazone: monomers, dimers, charge status, and cell mechanistic studies on triple negative breast cancer cells. Dalton Trans. 54 (9), 3872–3886. 10.1039/D4DT03445G 39883470

[B20] Fares J. Fares M. Y. Khachfe H. H. Salhab H. A. Fares Y. (2020). Molecular principles of metastasis: a hallmark of cancer revisited. Signal Transduct. Target Ther. 5 (1), 28. 10.1038/s41392-020-0134-x 32296047 PMC7067809

[B21] Farrugia L. J. (2012). WinGX and ORTEP for windows: an update. J. Appl. Crystallogr. 45 (4), 849–854. 10.1107/S0021889812029111

[B22] Fernández C. Y. Alvarez N. Rocha A. Mendes L. F. S. Costa-Filho A. J. Ellena J. (2024). Phenanthroline and phenyl carboxylate mixed ligand copper complexes in developing drugs to treat cancer. J. Inorg. Biochem. 260, 112700. 10.1016/j.jinorgbio.2024.112700 39163715

[B23] Ferraresi-Curotto V. Echeverría G. A. Piro O. E. Pis-Diez R. González-Baró A. C. (2015). Structural, spectroscopic and DFT study of 4-methoxybenzohydrazide schiff bases. A new series of polyfunctional ligands. Spectrochim. Acta A Mol. Biomol. Spectrosc. 137, 692–700. 10.1016/j.saa.2014.08.095 25255482

[B24] Ferretti V. León I. (2022). An overview of vanadium and cell signaling in potential cancer treatments. Inorganics (Basel) 10 (4), 47. 10.3390/inorganics10040047

[B25] Garcia‐Bosch I. Siegler M. A. (2016). Copper‐Catalyzed oxidation of alkanes with H2O2 under a fenton‐like regime. Angew. Chem. Int. Ed. 55 (41), 12873–12876. 10.1002/anie.201607216 27610603

[B26] Goodenough J. B. (1955). Theory of the role of covalence in the perovskite-type manganites [La, M(II)]MnO3. Phys. Rev. 100 (2), 564–573. 10.1103/PhysRev.100.564

[B27] Goodenough J. B. (1958). An interpretation of the magnetic properties of the perovskite-type mixed crystals La1−xSrxCoO3−λ. J. Phys. Chem. Solids 6 (2–3), 287–297. 10.1016/0022-3697(58)90107-0

[B28] Jungwirth U. Kowol C. R. Keppler B. K. Hartinger C. G. Berger W. Heffeter P. (2011). Anticancer activity of metal complexes: involvement of Redox processes. Antioxid. Redox Signal 15 (4), 1085–1127. 10.1089/ars.2010.3663 21275772 PMC3371750

[B29] Kanamori J. (1959). Superexchange interaction and symmetry properties of electron orbitals. J. Phys. Chem. Solids 10 (2–3), 87–98. 10.1016/0022-3697(59)90061-7

[B30] Kansara M. Teng M. W. Smyth M. J. Thomas D. M. (2014). Translational biology of osteosarcoma. Nat. Rev. Cancer 14 (11), 722–735. 10.1038/nrc3838 25319867

[B31] Kondo J. Inoue M. (2019). Application of cancer organoid model for drug screening and personalized therapy. Cells 8 (5), 470. 10.3390/cells8050470 31108870 PMC6562517

[B32] Kooy N. Royall J. Ischiropoulos H. Beckman J. (1994). Peroxynitrite-mediated oxidation of dihydrorhodamine 123. Free Radic. Biol. Med. 16 (2), 149–156. 10.1016/0891-5849(94)90138-4 8005510

[B33] Kramer N. Walzl A. Unger C. Rosner M. Krupitza G. Hengstschläger M. (2013). *In vitro* cell migration and invasion assays. Mutat. Research/Reviews Mutat. Res. 752 (1), 10–24. 10.1016/j.mrrev.2012.08.001 22940039

[B34] Lee S. Y. Kim C. Y. Nam T. G. (2020). Ruthenium complexes as anticancer agents: a brief history and perspectives. Drug Des. Devel Ther. 14, 5375–5392. 10.2147/DDDT.S275007 33299303 PMC7721113

[B35] León I. E. (2024). Transition metal complexes: a new generation of anticancer drugs. Future Med. Chem. 16, 1727–1730. 10.1080/17568919.2024.2383166 39119691 PMC11457602

[B36] León I. E. Cadavid-Vargas J. F. Resasco A. Maschi F. Ayala M. A. Carbone C. (2016). *In vitro* and *in vivo* antitumor effects of the VO-chrysin complex on a new three-dimensional osteosarcoma spheroids model and a xenograft tumor in mice. J. Biol. Inorg. Chem. 21 (8), 1009–1020. 10.1007/s00775-016-1397-0 27696106

[B37] Levín P. Ruiz M. C. Romo A. I. B. Nascimento O. R. Di Virgilio A. L. Oliver A. G. (2021). Water-mediated reduction of [Cu(dmp)2(CH3CN)]2+: implications of the structure of a classical complex on its activity as an anticancer drug. Inorg. Chem. Front. 8 (13), 3238–3252. 10.1039/d1qi00233c

[B38] Li S. Zhang H. Liu J. Shang G. (2023). Targeted therapy for osteosarcoma: a review. J. Cancer Res. Clin. Oncol. 149 (9), 6785–6797. 10.1007/s00432-023-04614-4 36807762 PMC11796608

[B39] Mitrakas A. G. Tsolou A. Didaskalou S. Karkaletsou L. Efstathiou C. Eftalitsidis E. (2023). Applications and advances of multicellular tumor spheroids: challenges in their development and analysis. Int. J. Mol. Sci. 24 (8), 6949. 10.3390/ijms24086949 37108113 PMC10138394

[B40] Moreno F. Cacciavillano W. Cipolla M. Coirini M. Streitenberger P. López Martí J. (2017). Childhood osteosarcoma: incidence and survival in Argentina. Report from the National Pediatric Cancer Registry, ROHA Network 2000–2013. Pediatr. Blood Cancer 64 (10), e26533. 10.1002/pbc.26533 28409896

[B41] Mosmann T. (1983). Rapid colorimetric assay for cellular growth and survival: application to proliferation and cytotoxicity assays. J. Immunol. Methods. 65 (1‐2), 55–63. 10.1016/0022-1759(83)90303-4 6606682

[B42] Qin Y. Lu M. Gong X. (2008). Dihydrorhodamine 123 is superior to 2,7‐dichlorodihydrofluorescein diacetate and dihydrorhodamine 6G in detecting intracellular hydrogen peroxide in tumor cells. Cell Biol. Int. 32 (2), 224–228. 10.1016/j.cellbi.2007.08.028 17920943

[B43] Rigaku (2015). Rigaku. Crysalispro 1.171.38.41. UK: Oxford Diffraction.

[B44] Rodríguez M. R. Del Plá J. Balsa L. M. León I. E. Piro O. E. Echeverría G. A. (2019). Cu(II) and Zn(II) complexes with a poly-functional ligand derived from: o -vanillin and thiophene. Crystal structure, physicochemical properties, theoretical studies and cytotoxicity assays against human breast cancer cells. New J. Chem. 43 (18), 7120–7129. 10.1039/c8nj06274a

[B45] Rodríguez M. R. Balsa L. M. Piro O. E. Etcheverría G. A. García-Tojal J. Pis-Diez R. (2021). Synthesis, crystal structure, spectroscopic characterization, DFT calculations and cytotoxicity assays of a new Cu(II) complex with an acylhydrazone ligand derived from thiophene. Inorganics (Basel) 9 (2), 9. 10.3390/inorganics9020009

[B46] Romo A. I. B. Carepo M. P. Levín P. Nascimento O. R. Díaz D. E. Rodríguez-López J. (2021). Synergy of DNA intercalation and catalytic activity of a copper complex towards improved polymerase inhibition and cancer cell cytotoxicity. Dalton Trans. 50 (34), 11931–11940. 10.1039/D1DT01358K 34374389

[B47] Ruiz M. C. Kljun J. Turel I. Di Virgilio A. L. León I. E. (2019). Comparative antitumor studies of organoruthenium complexes with 8-hydroxyquinolines on 2D and 3D cell models of bone, lung and breast cancer. Metallomics 11 (3), 666–675. 10.1039/c8mt00369f 30839008

[B48] Santa Maria de la Parra L. Assandri M. H. Solernó L. M. Serradell M. de los A. Alonso D. F. Garona J. (2026a). *In vitro* and *in vivo* effects of a Copper(II)-Hydrazone complex against human Osteosarcoma. Pharmaceutics 18 (3), 372. 10.3390/pharmaceutics18030372 41900858 PMC13029629

[B49] Santa Maria de la Parra L. Martínez V. R. Nayeem N. Contel M. León I. E. (2026b). Exploring the anticancer properties and mode of action of Copper(II)‐Furan acylhydrazone on human triple negative breast cancer cells. ChemMedChem 21 (4), e202500836. 10.1002/cmdc.202500836 41705660

[B50] Santa Maria de la Parra L. Balsa L. M. León I. E. (2024a). Metallocompounds as anticancer agents against osteosarcoma. Drug Discovery today. Drug Discov. Today 29, 104100. 10.1016/j.drudis.2024.104100 39019429

[B51] Santa Maria de la Parra L. Romo A. I. B. Rodríguez-López J. Nascimento O. R. Echeverría G. A. Piro O. E. (2024b). Promising dual anticancer and antimetastatic action by a Cu(II) complex derived from acylhydrazone on human Osteosarcoma models. Inorg. Chem. 63 (11), 4925–4938. 10.1021/acs.inorgchem.3c04085 38442008

[B52] Santa Maria de la Parra L. Rocha M. de la Sovera V. Echeverría G. A. Piro O. E. Scalese G. (2026). Synthesis, crystal structure, spectroscopic characterization, theoretical studies and cytotoxicity assays of novel two acylhydrazones and its Cu(II) complexes. J. Inorg. Biochem. 279, 113243. 10.1016/j.jinorgbio.2026.113243 41813535

[B53] Santini C. Pellei M. Gandin V. Porchia M. Tisato F. Marzano C. (2014). Advances in copper complexes as anticancer agents. Chem. Rev. 114, 815–862. 10.1021/cr400135x 24102434

[B54] Schumacker P. T. (2006). Reactive oxygen species in cancer cells: live by the sword, die by the sword. Cancer Cell 10 (3), 175–176. 10.1016/j.ccr.2006.08.015 16959608

[B55] Sheldrick G. M. (2008). A short history of SHELX. Acta Crystallogr. A 64 (1), 112–122. 10.1107/S0108767307043930 18156677

[B56] Sheldrick G. M. (2015). SHELXT– integrated space-group and crystal-structure determination. Acta Crystallogr. A Found. Adv. 71 (1), 3–8. 10.1107/S2053273314026370 25537383 PMC4283466

[B57] Tang C. B. (2008). N′-(2-Hydroxybenzylidene)-4-methoxybenzohydrazide. Acta Crystallogr. Sect. E Struct. Rep. Online 64 (4), o767. 10.1107/S1600536808008088 21202154 PMC2961042

[B58] Valko M. Rhodes C. J. Moncol J. Izakovic M. Mazur M. (2006). Free radicals, metals and antioxidants in oxidative stress-induced cancer. Chem. Biol. Interact. 160 (1), 1–40. 10.1016/j.cbi.2005.12.009 16430879

