# Supplementary material for: Development of novel Copper(II)–hydrazone complexes for targeting the inhibition of human osteosarcoma growth
Source: Front Chem. 2026 Oct 1;14:1897476. doi: 10.3389/fchem.2026.1897476 (PMC13670372; doi:10.3389/fchem.2026.1897476)
Supplement: Supplementary file 1 [file DataSheet1.pdf]

Supplementary Material

1 Supplementary Figures and Tables

1.1 Supplementary Figures

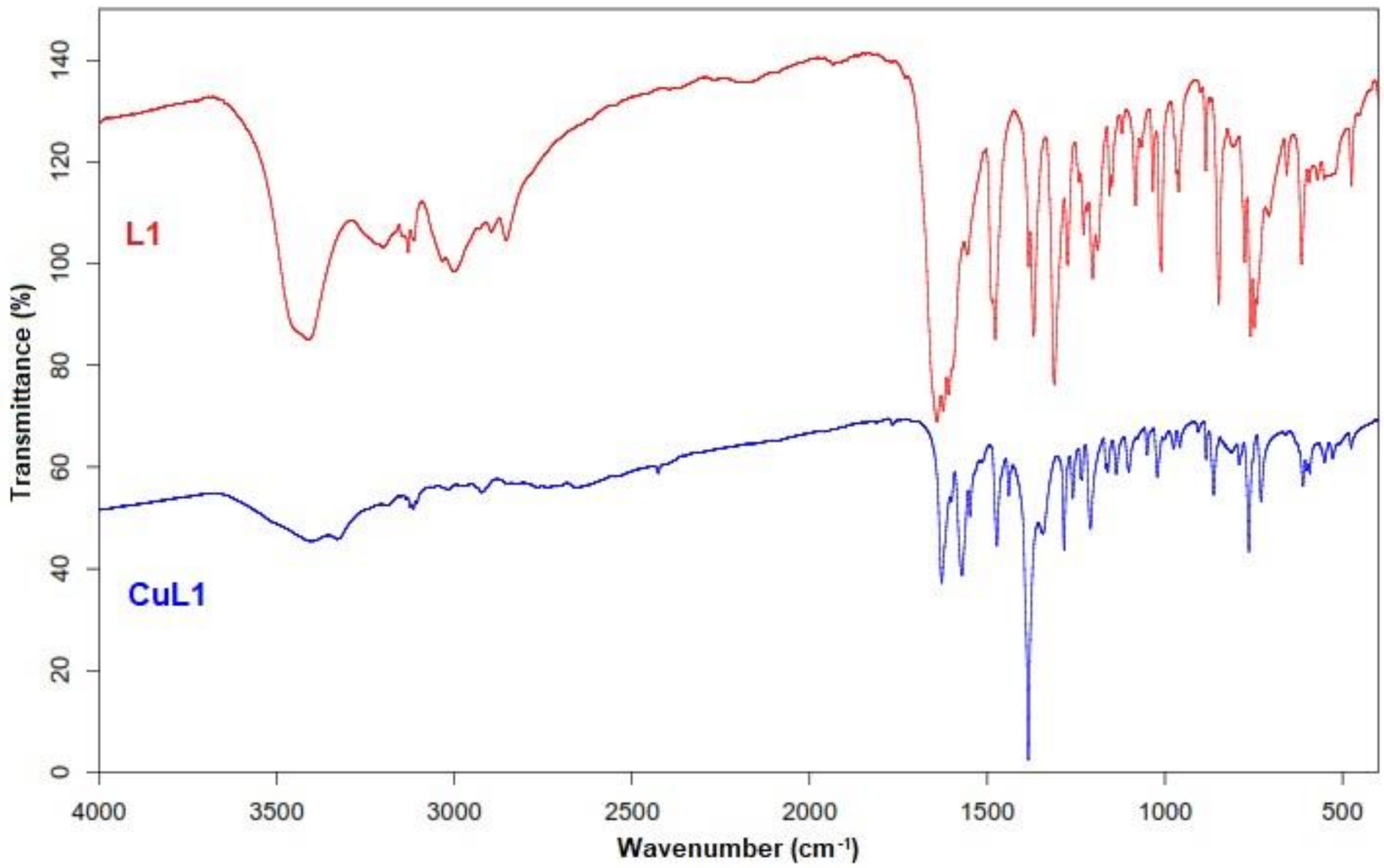

Supplementary Figure 1. Infrared spectra of L1 (red line) and CuL1 complex (blue line) in KBr pellets.

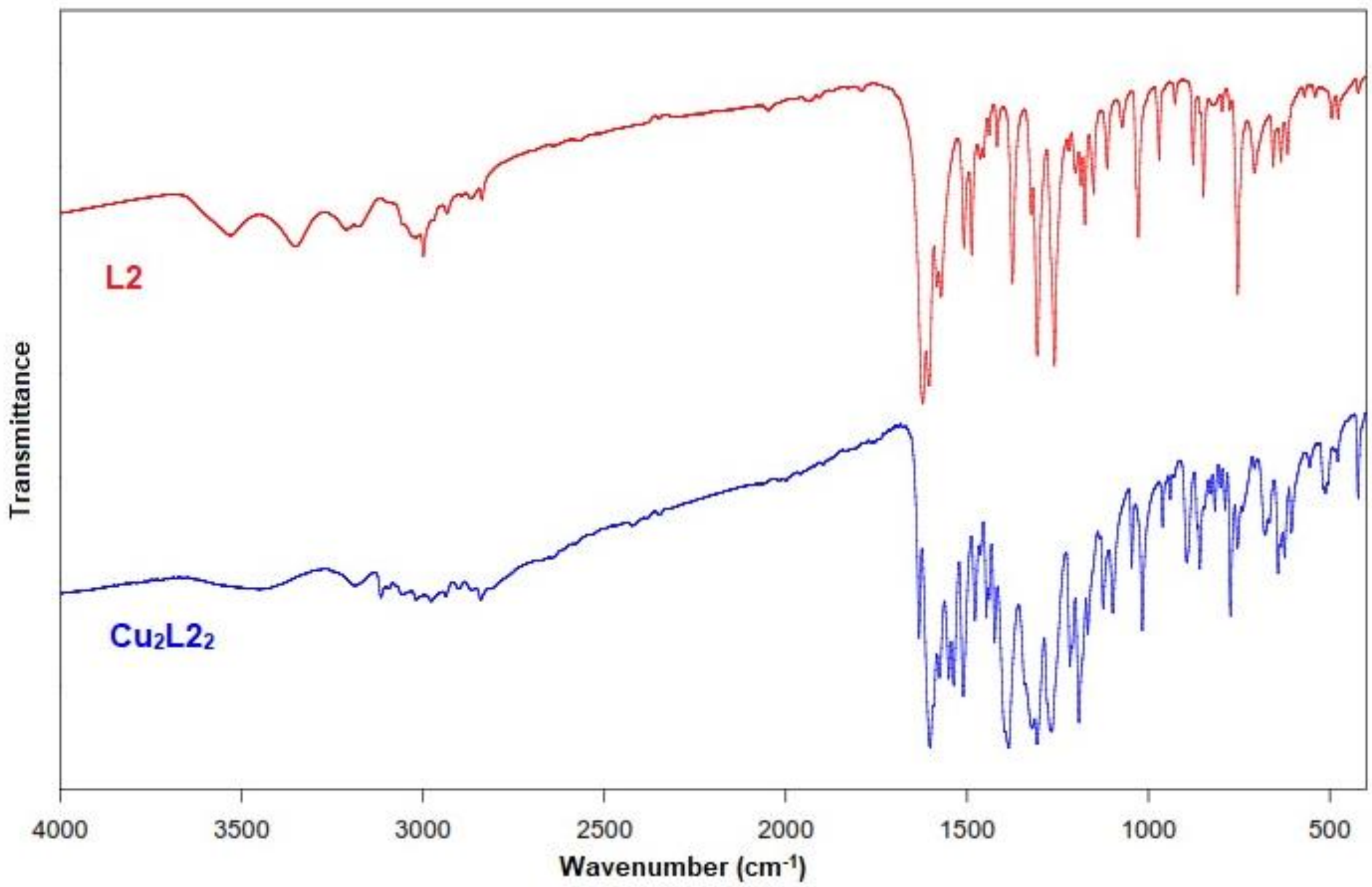

Supplementary Figure 2. Infrared spectra of L2 (red line) and Cu_2L2_2 complex (blue line) in KBr pellets.

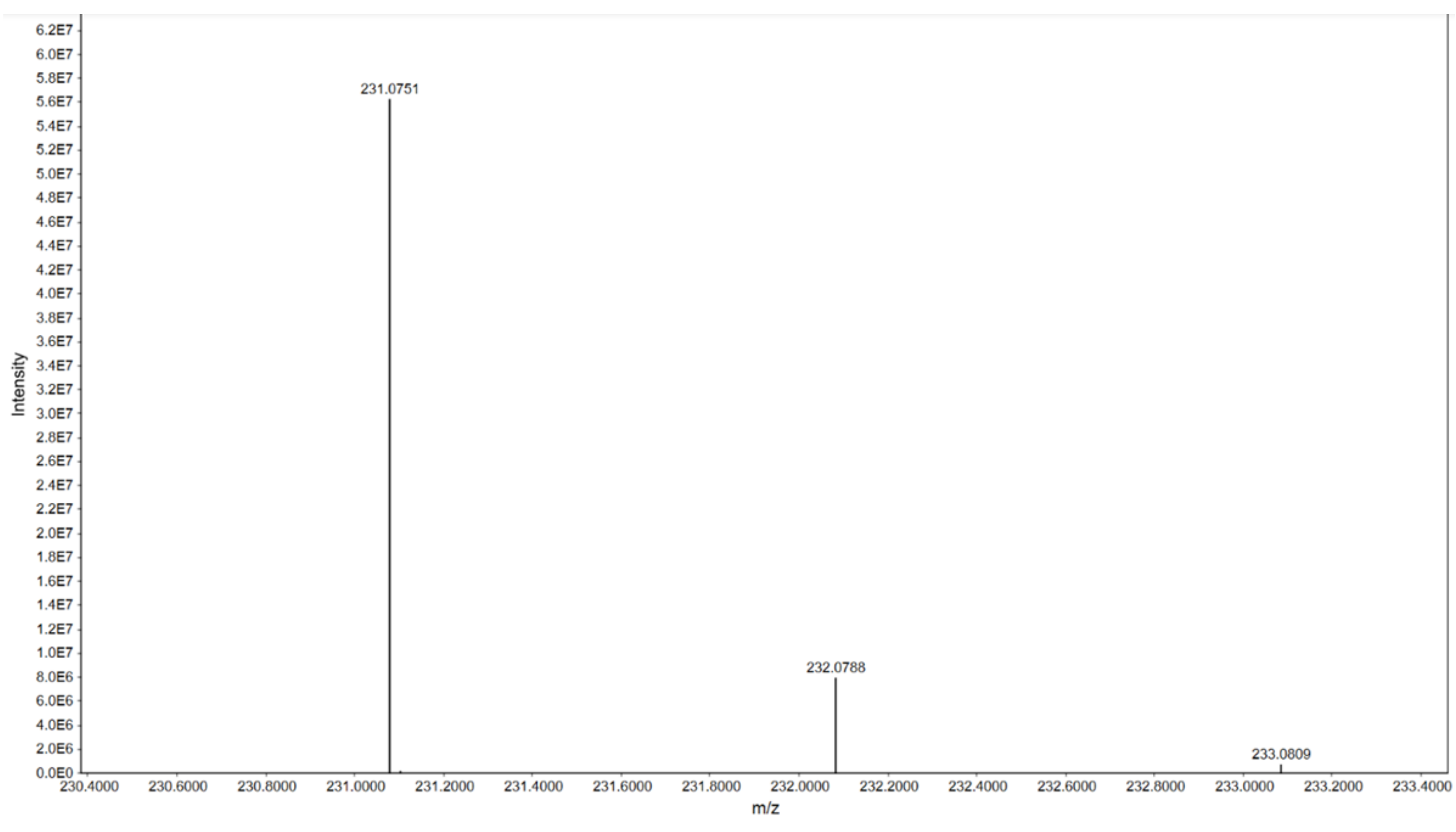

Supplementary Figure 3. High-resolution mass spectrum (HRMS, ESI$^+$) of the compound L1 showing the protonated molecular ion.

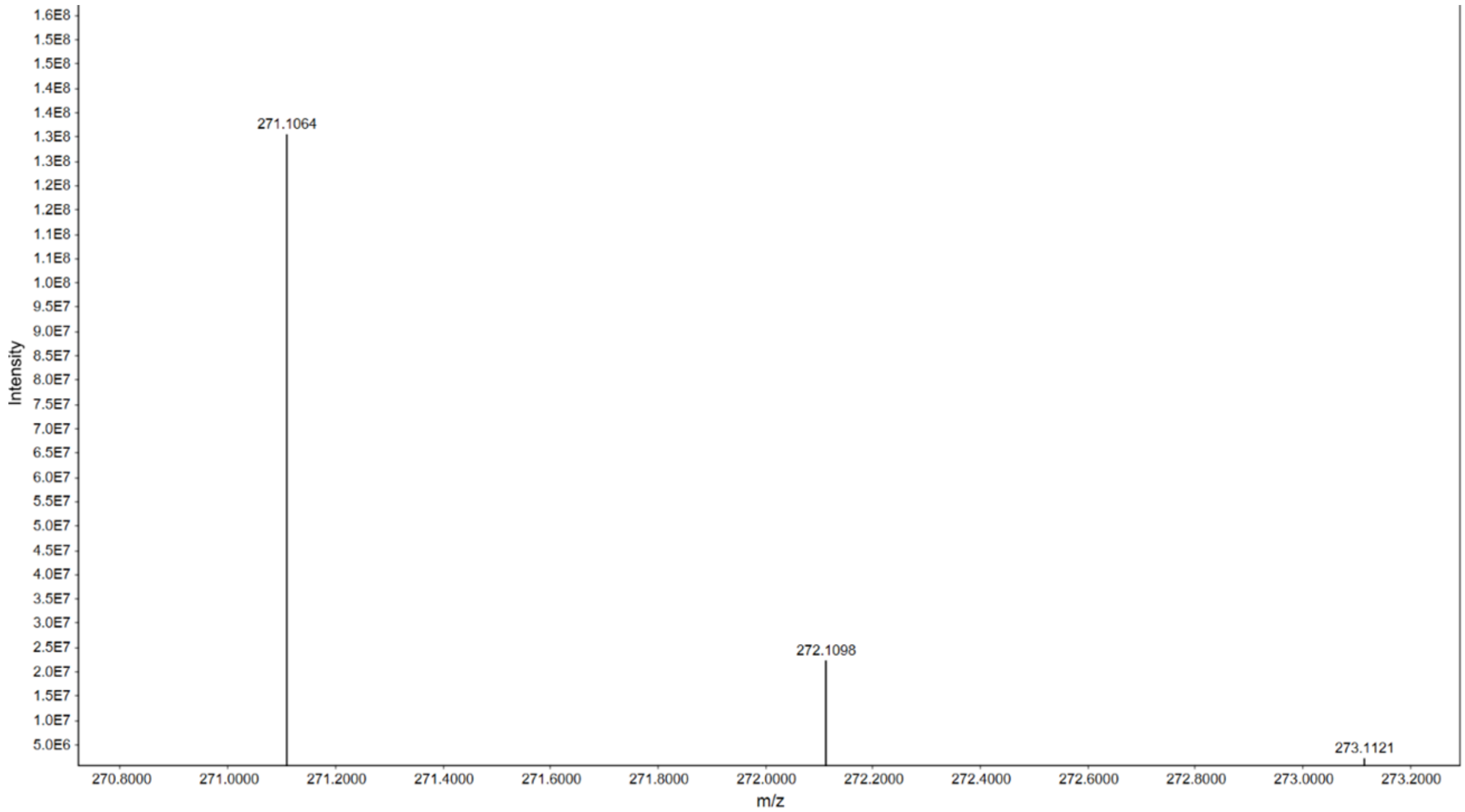

Supplementary Figure 4. High-resolution mass spectrum (HRMS, ESI$^+$) of the compound L2 showing the protonated molecular ion.

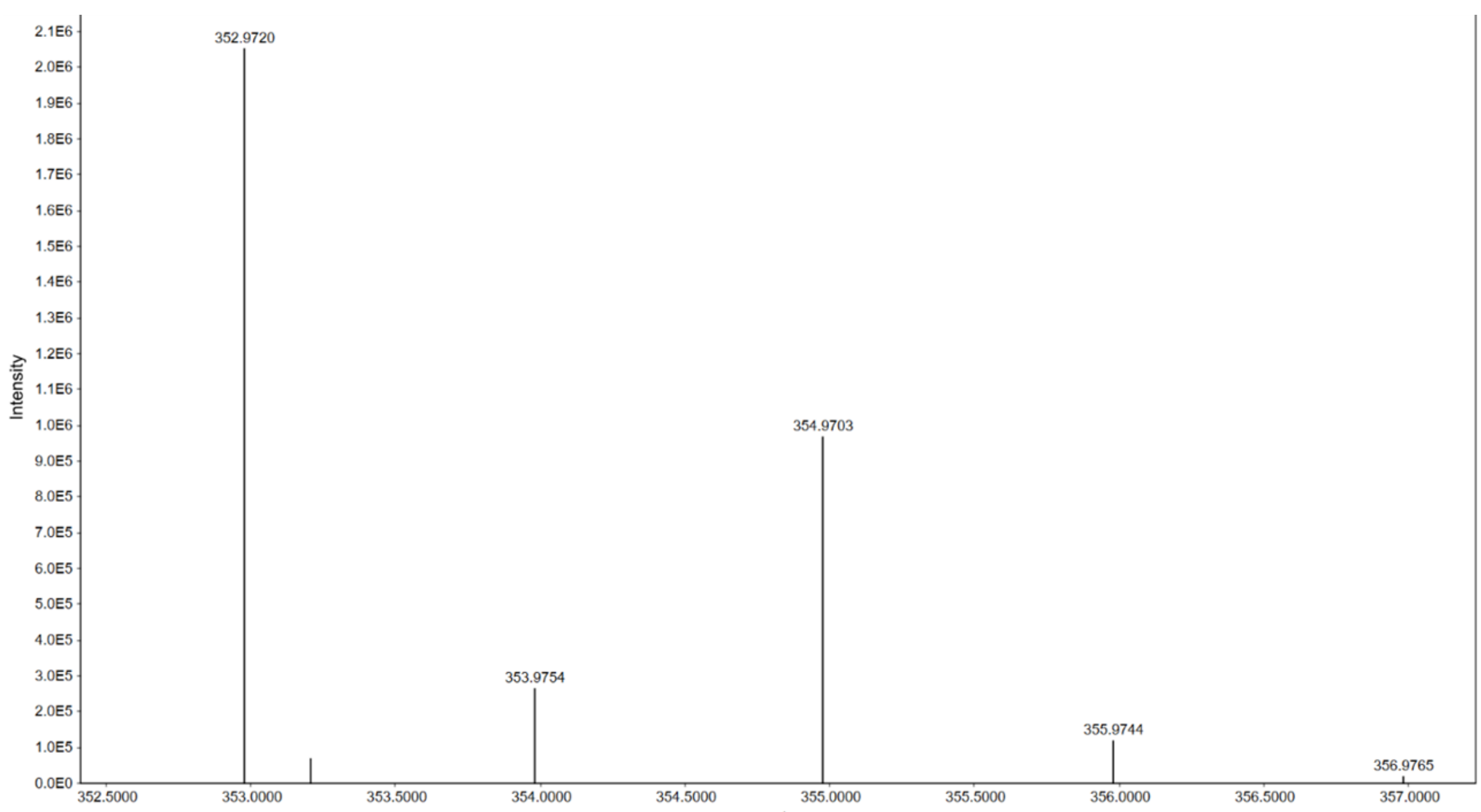

Supplementary Figure 5. High-resolution ESI mass spectrometry analysis in negative mode of the copper complex showing the isotopic pattern corresponding to the assigned species [Cu(L1-2H)(NO_3)]$^-$.

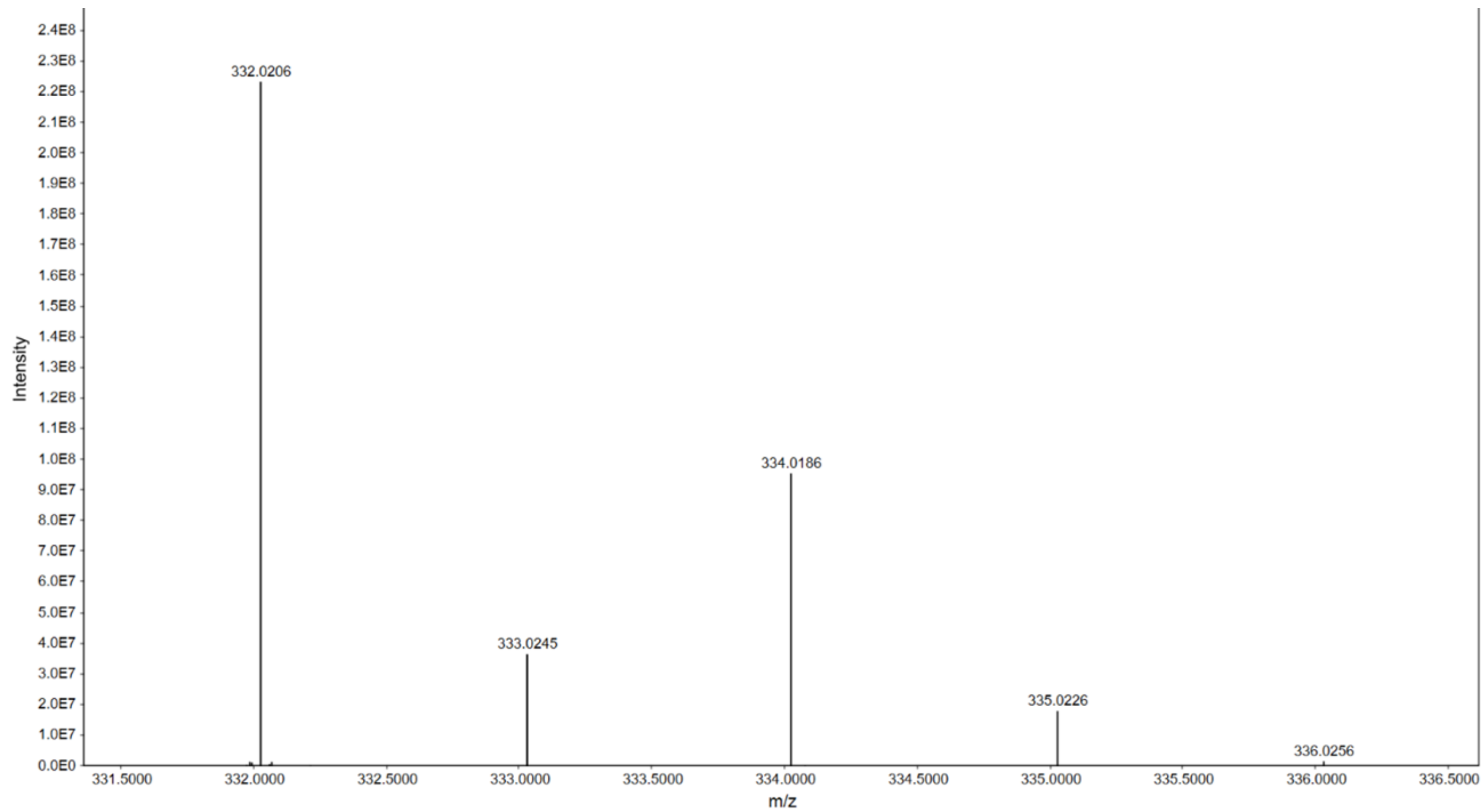

Supplementary Figure 6. High-resolution ESI mass spectrometry analysis in positive mode of the copper complex showing the isotopic pattern corresponding to the assigned species [Cu_2(μ-L2-H)$_2$]$^{2+}$.

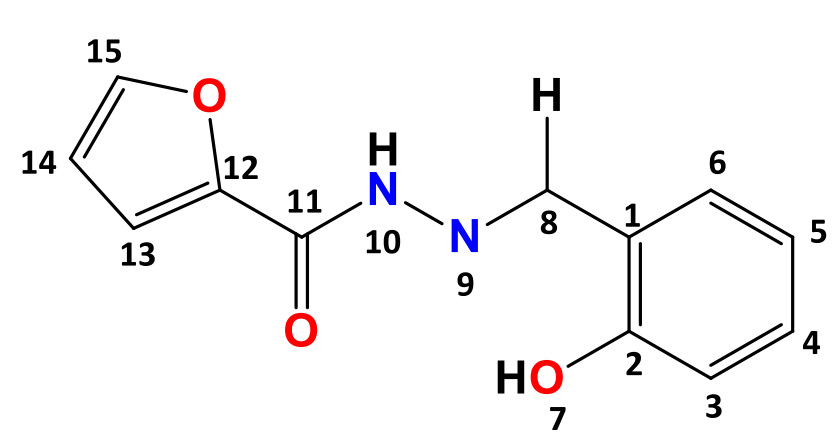

L1

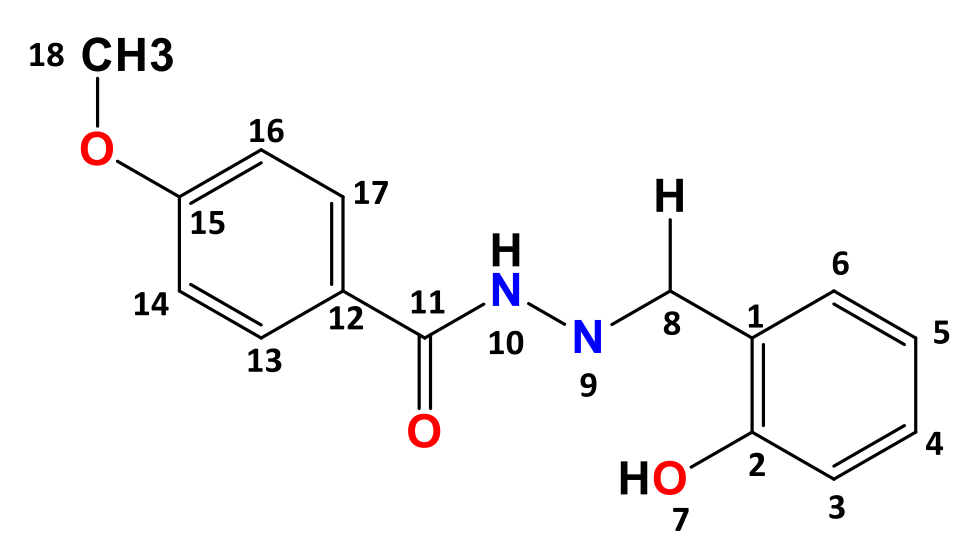

L2

Supplementary Figure 7. Numbering of free L ligands (L1 and L2) used for NMR assignments.

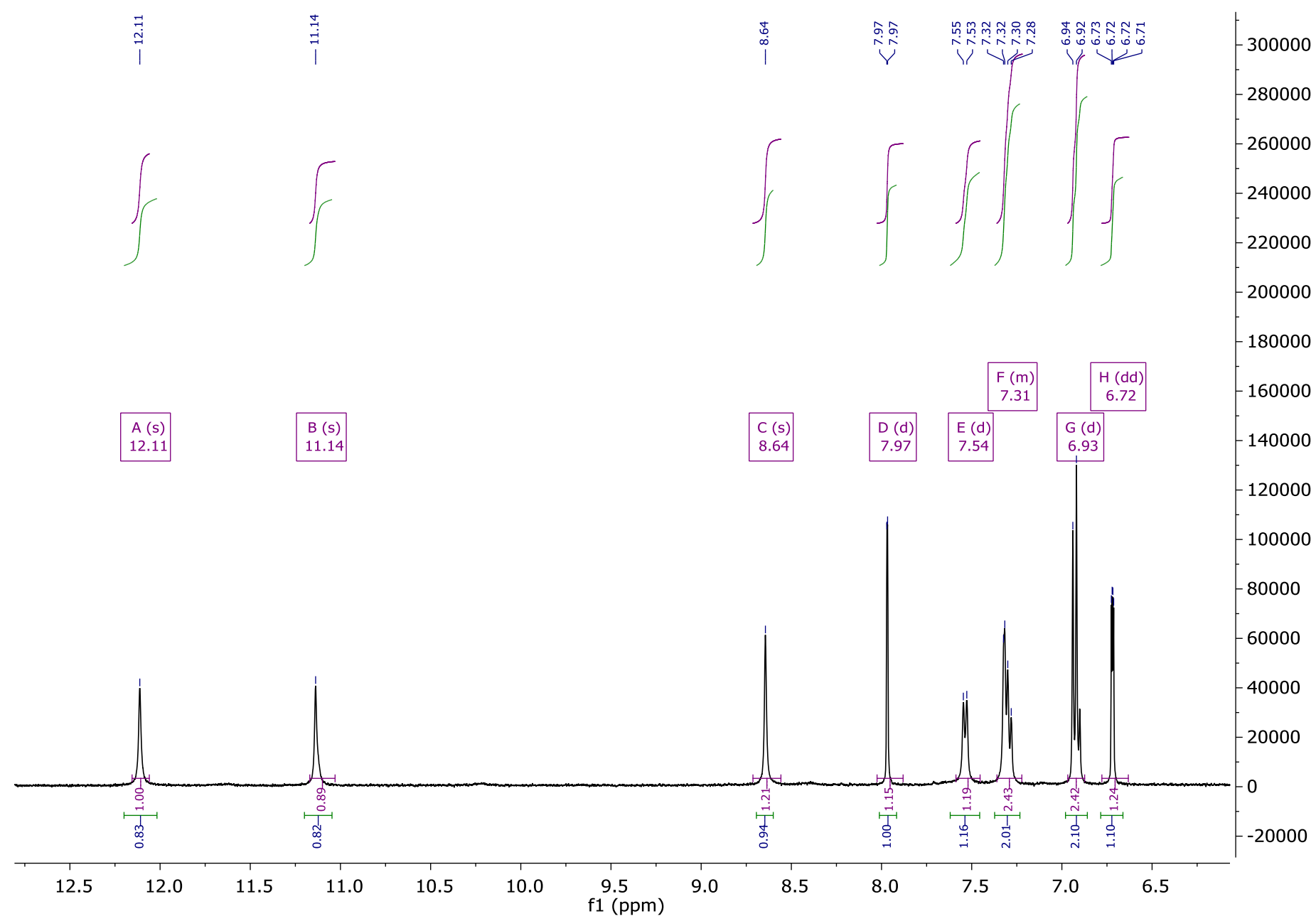

Supplementary Figure 8. ^{1}H NMR spectrum (400 MHz, DMSO-d_6) of L1

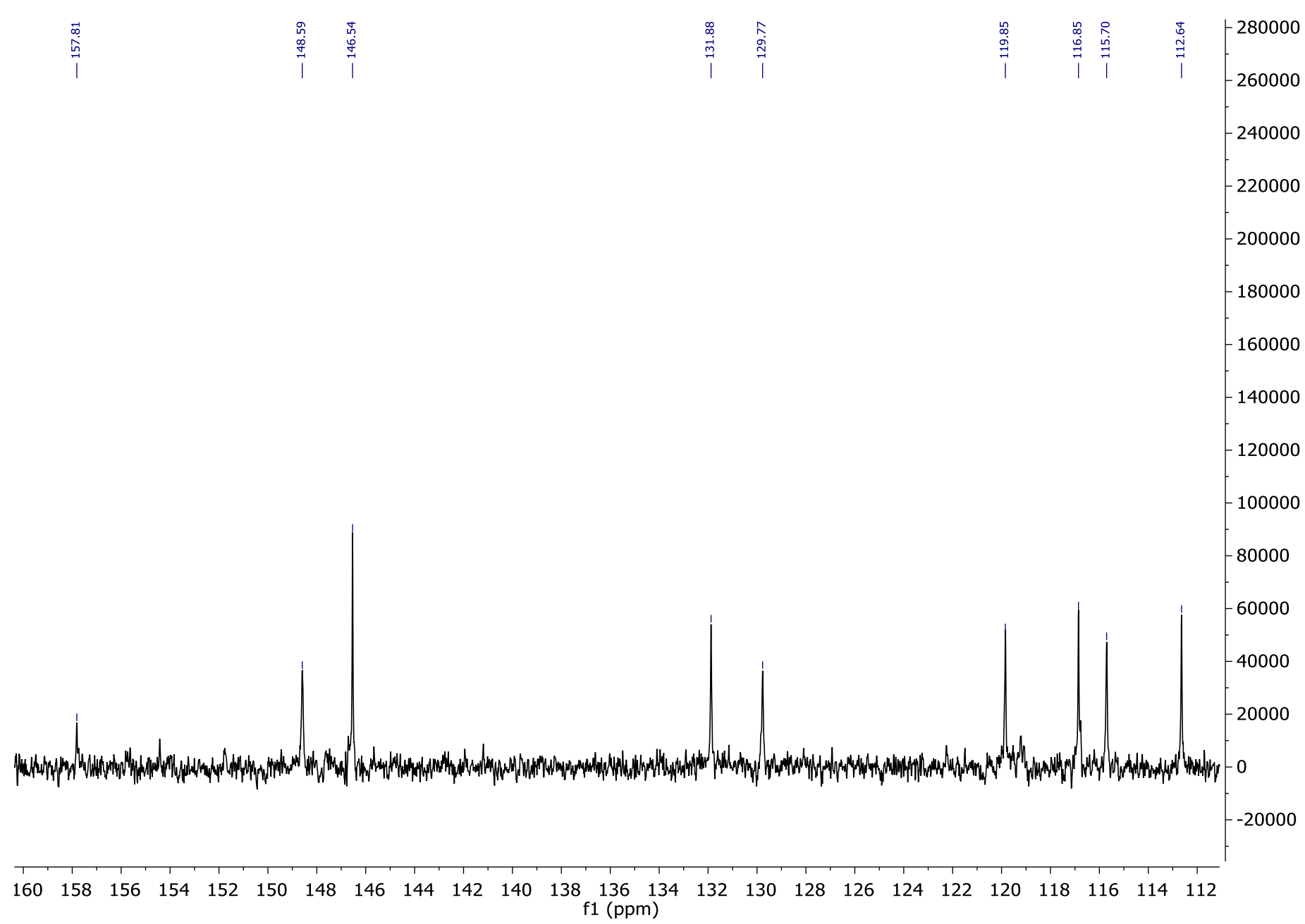

Supplementary Figure 9. ^{13}C NMR spectrum (101 MHz, DMSO-d_6) of L1

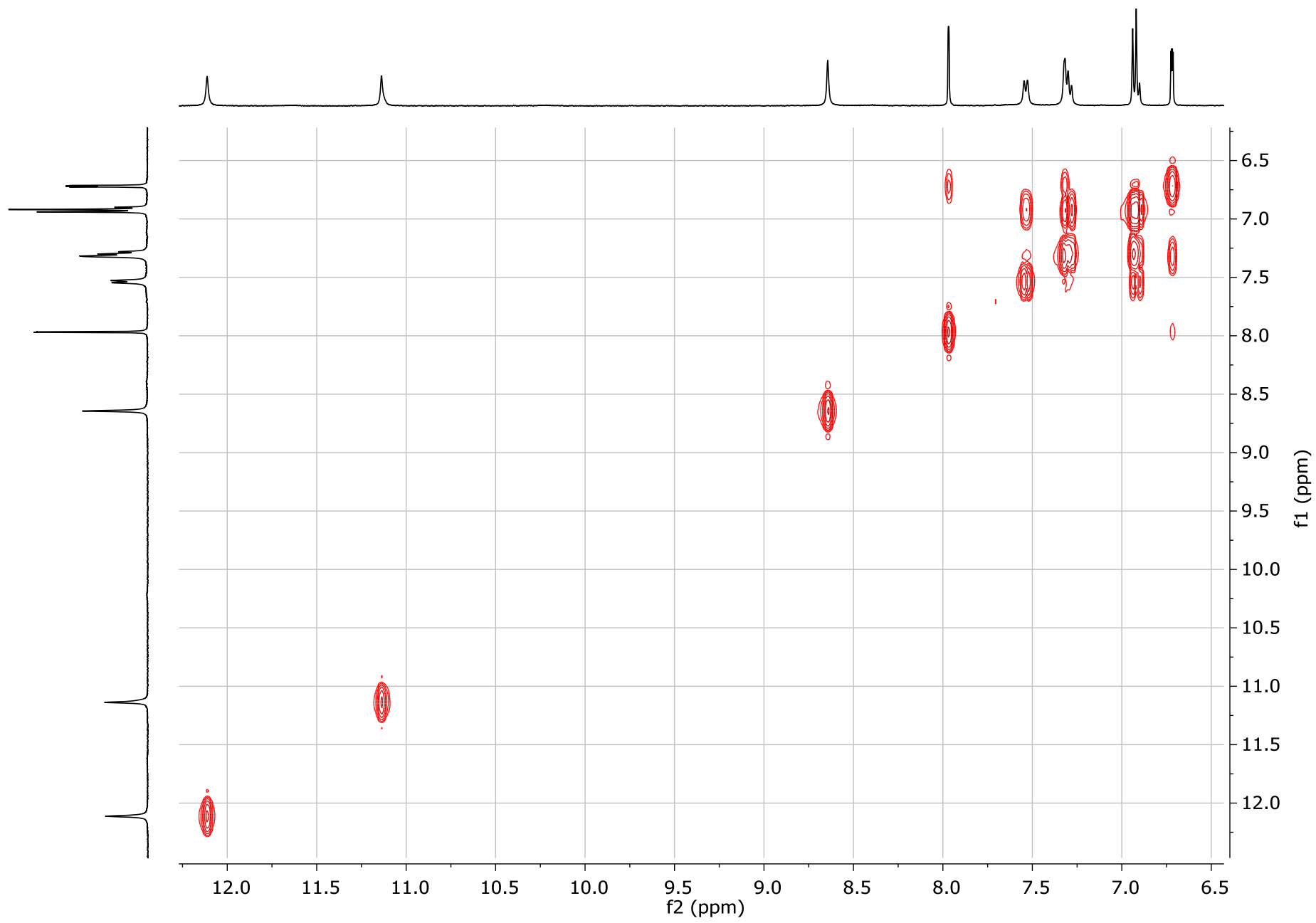

Supplementary Figure 10. 2D ^1H–^1H COSY spectrum (DMSO-d_6) of L1, showing the cross-peaks corresponding to scalar couplings between proton environments.

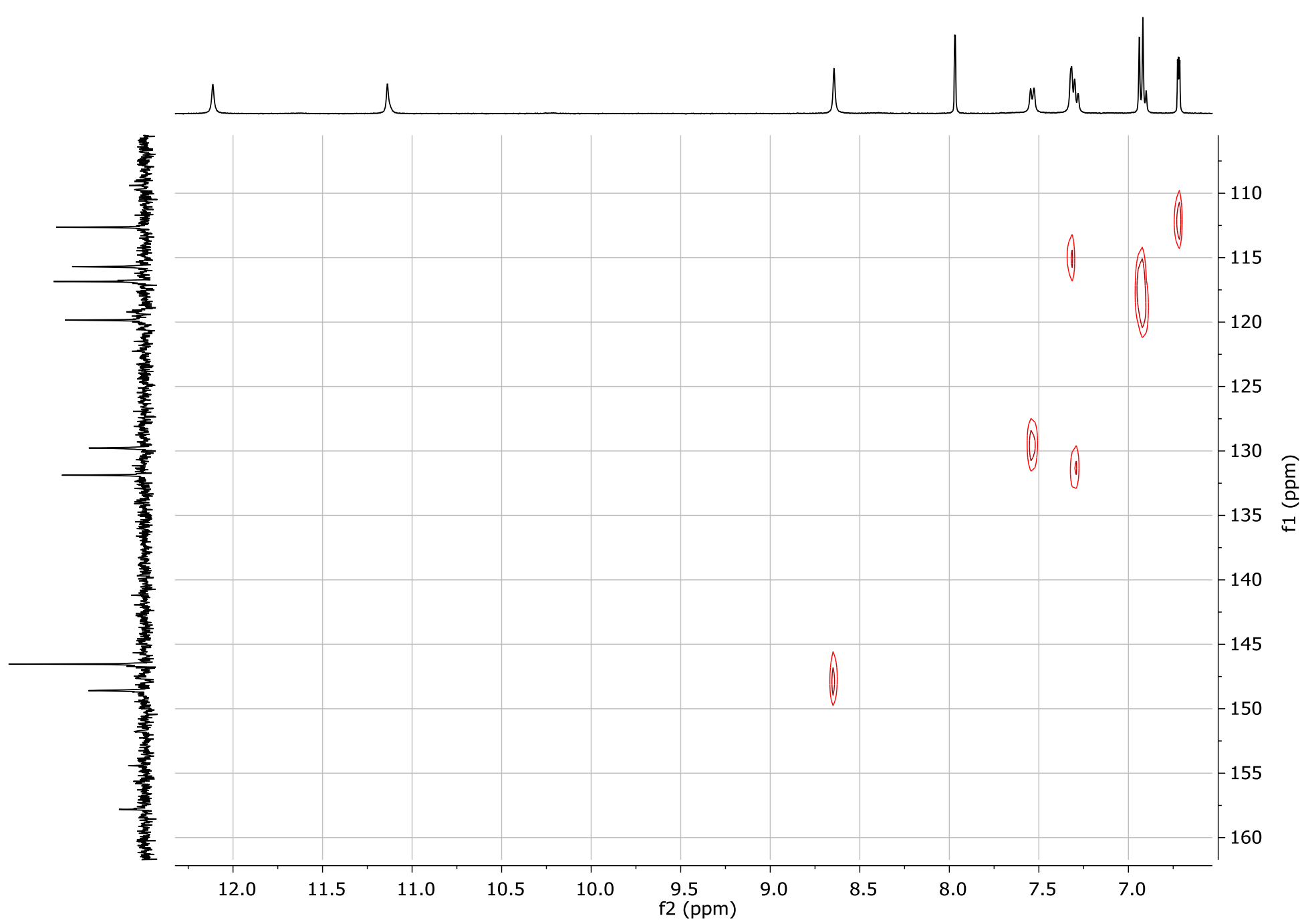

Supplementary Figure 11. 2D HSQC spectrum (DMSO-d_6) of L1, showing direct one-bond ^1H–^{13}C correlations used to confirm proton–carbon assignments.

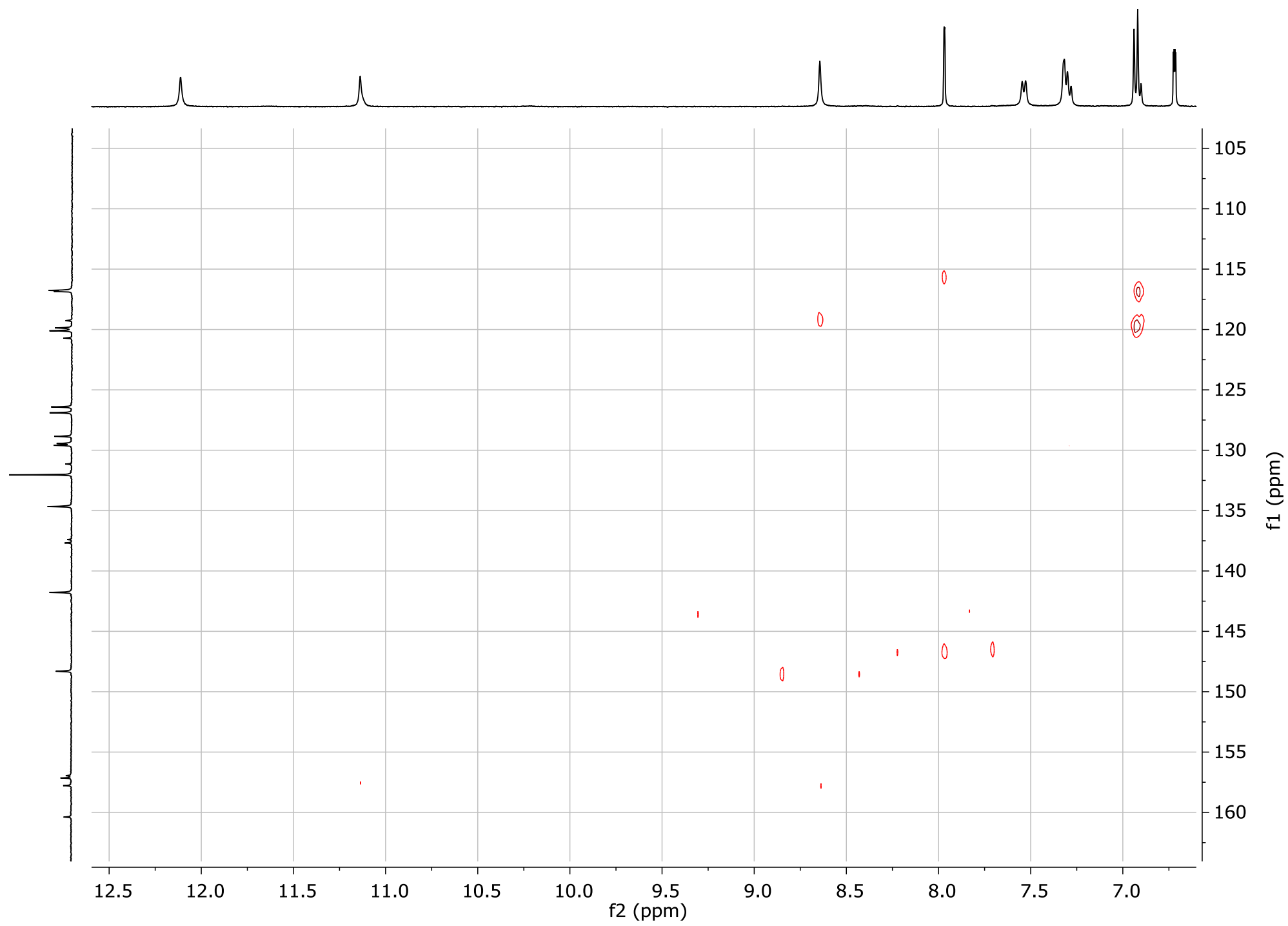

Supplementary Figure 12. 2D HSQC spectrum (DMSO-d₆) of L1, showing direct one-bond ¹H–¹³C correlations used to confirm proton–carbon assignments.

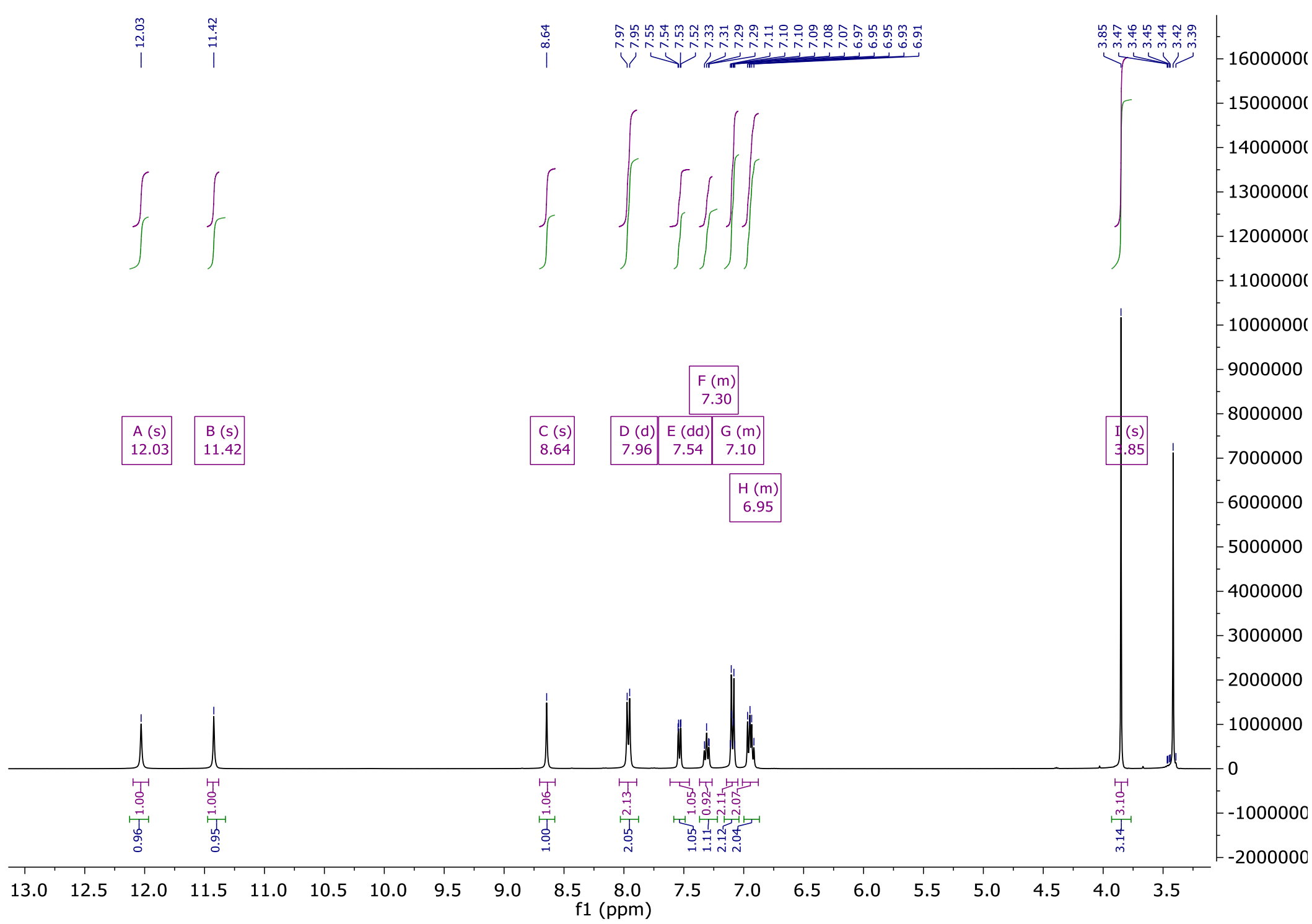

Supplementary Figure 13. ¹H NMR spectrum (400 MHz, DMSO-d₆) of L2

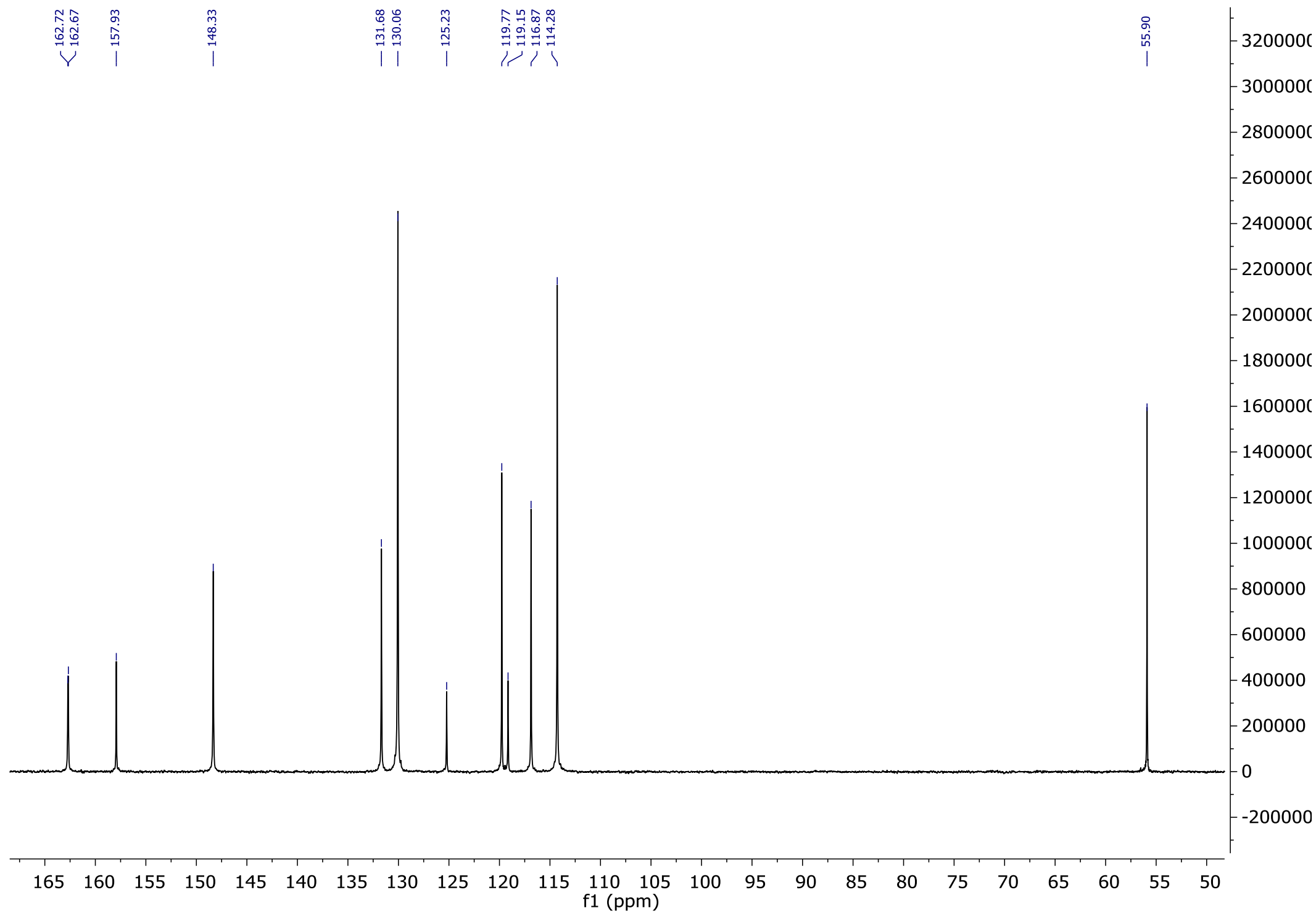

Supplementary Figure 14. ^{13}C NMR spectrum (101 MHz, DMSO-d_6) of L2

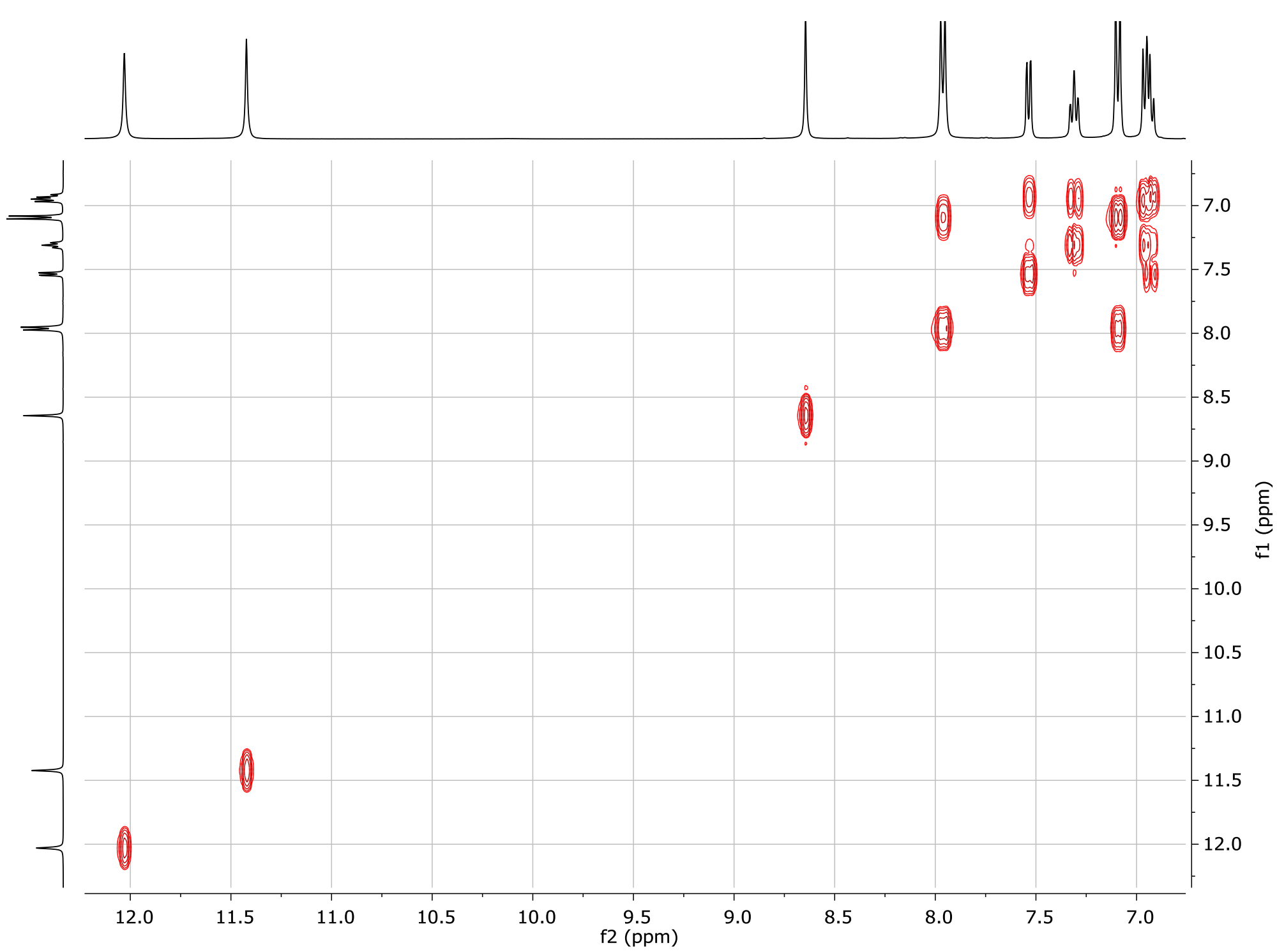

Supplementary Figure 15. 2D ^1H–^1H COSY spectrum (DMSO-d_6) of L2, showing the cross-peaks corresponding to scalar couplings between proton environments.

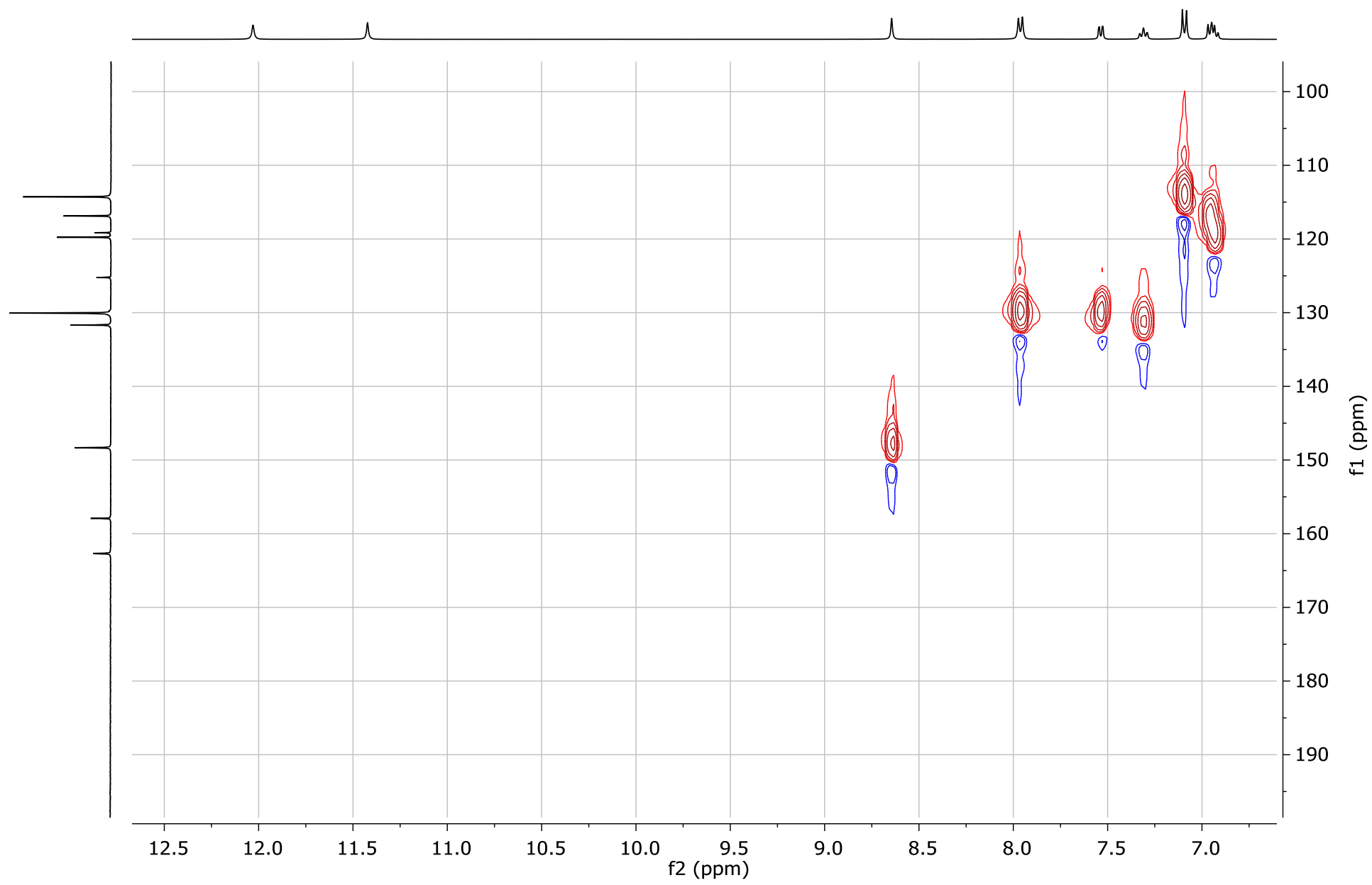

Supplementary Figure 16. 2D HSQC spectrum (DMSO-d₆) of L2, showing direct one-bond ^{1}H–^{13}C correlations used to confirm proton–carbon assignments.

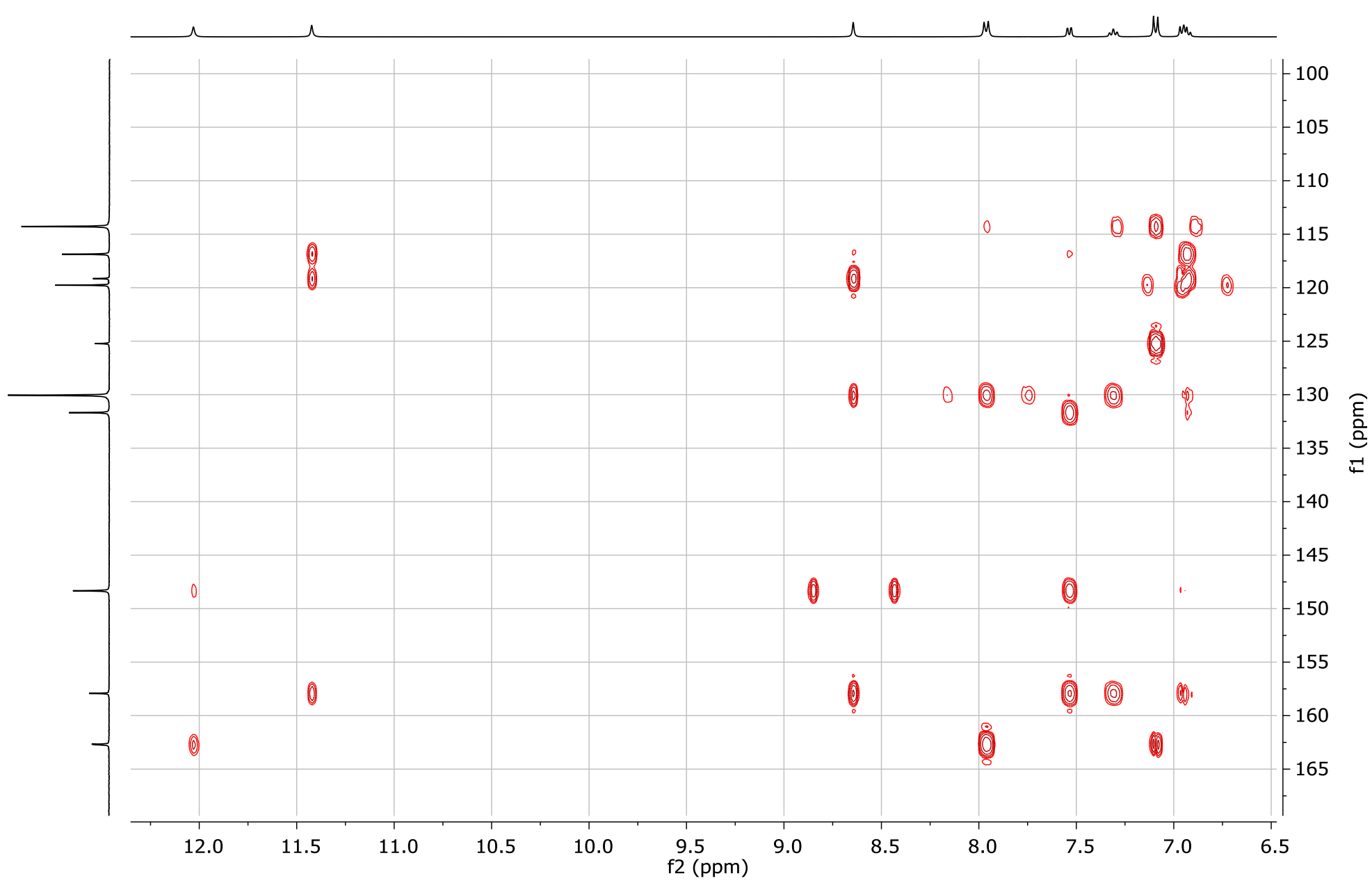

Supplementary Figure 17. 2D HMBC spectrum (DMSO-d₆) of L2, showing direct one-bond ^{1}H–^{13}C correlations used to confirm proton–carbon assignments.

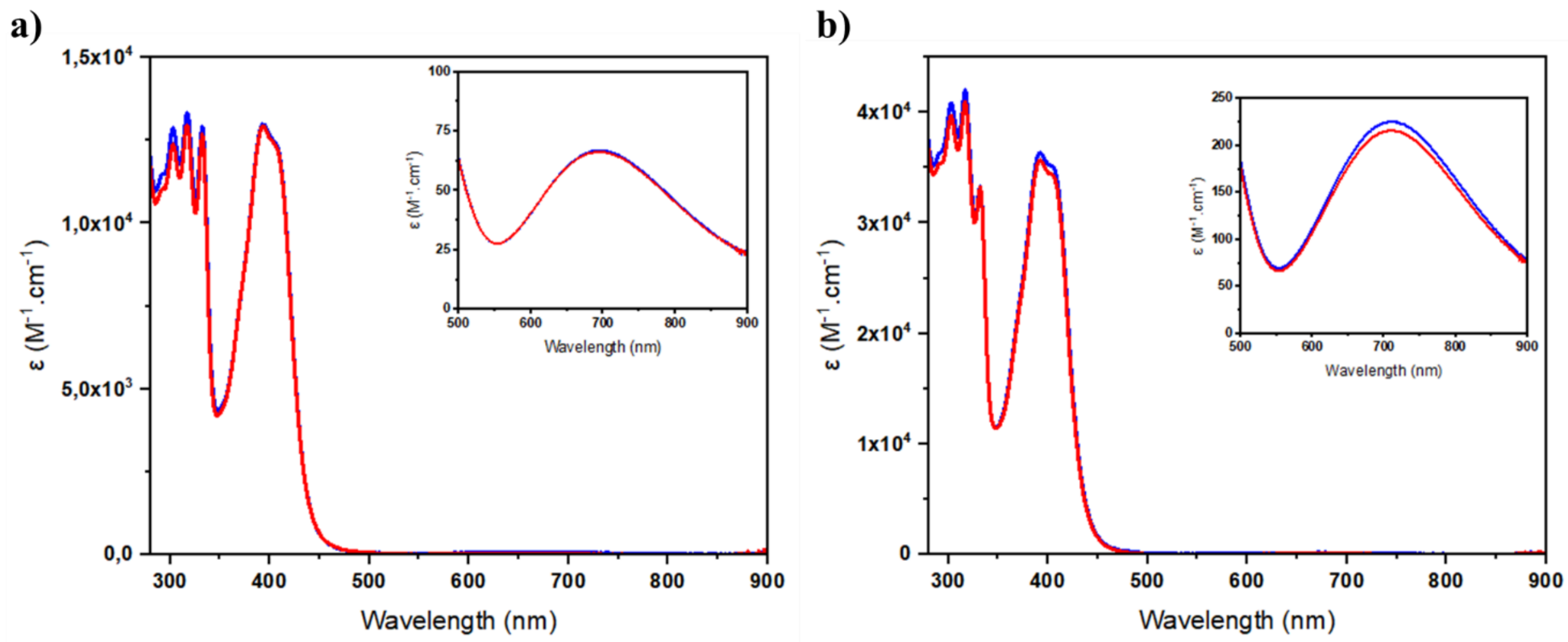

Supplementary Figure 18. Electronic absorption spectra in UV−vis region obtained at 0 h (blue line) and 24 h (red line) of a) CuL1 performed in DMSO at 5 × 10^{-5} and 5 × 10^{-3} M (inset) and b) Cu_2L2_2 performed in DMSO at 2.5 × 10^{-5} M and 2.5 × 10^{-3} M (inset).

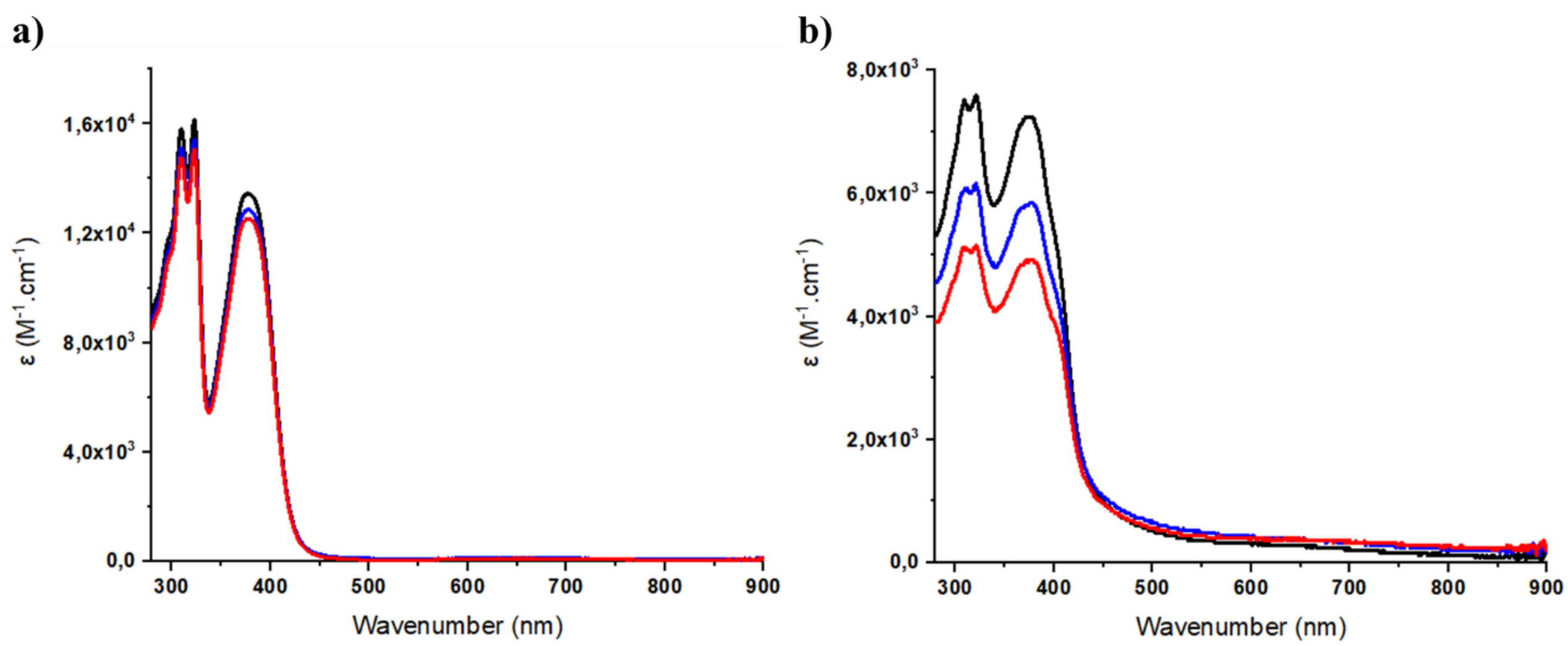

Supplementary Figure 19. Electronic absorption spectra in UV−vis region obtained at 0 h (black line), 3 h (blue line) and 24 h (red line) of a) CuL1 performed in PBS at 5 × 10^{-5} and b) Cu_2L2_2 performed in PBS at 2.5 × 10^{-5} M.

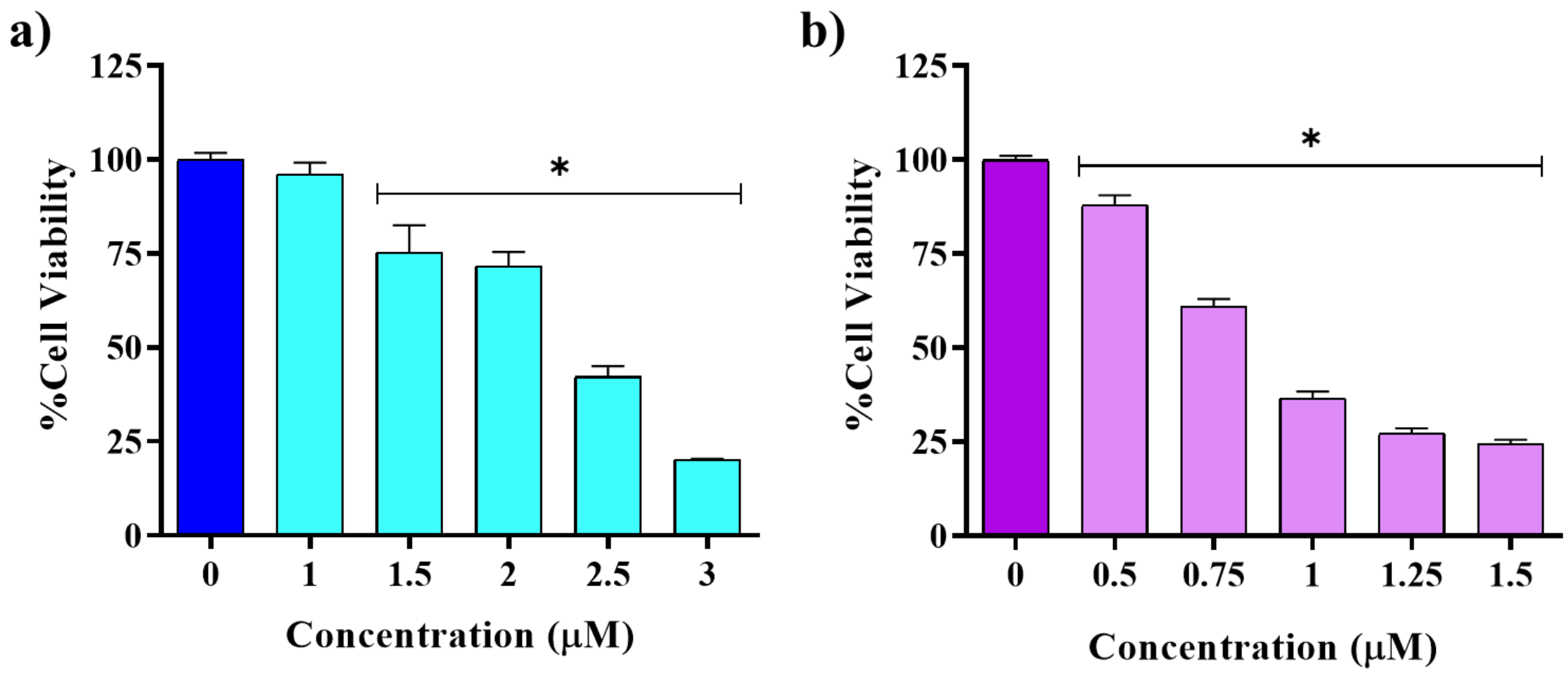

Supplementary Figure 20. Cellular viability was evaluated via MTT assay. MG-63 cells were incubated in Dulbecco's modified Eagle's medium (DMEM) alone (Control) or with different concentrations of a) CuL1 or b) Cu_2L2_2 for 24 h. The results are expressed as the mean ± the standard error of the mean (SEM). *p <0.0001 differences between Control and treatment.

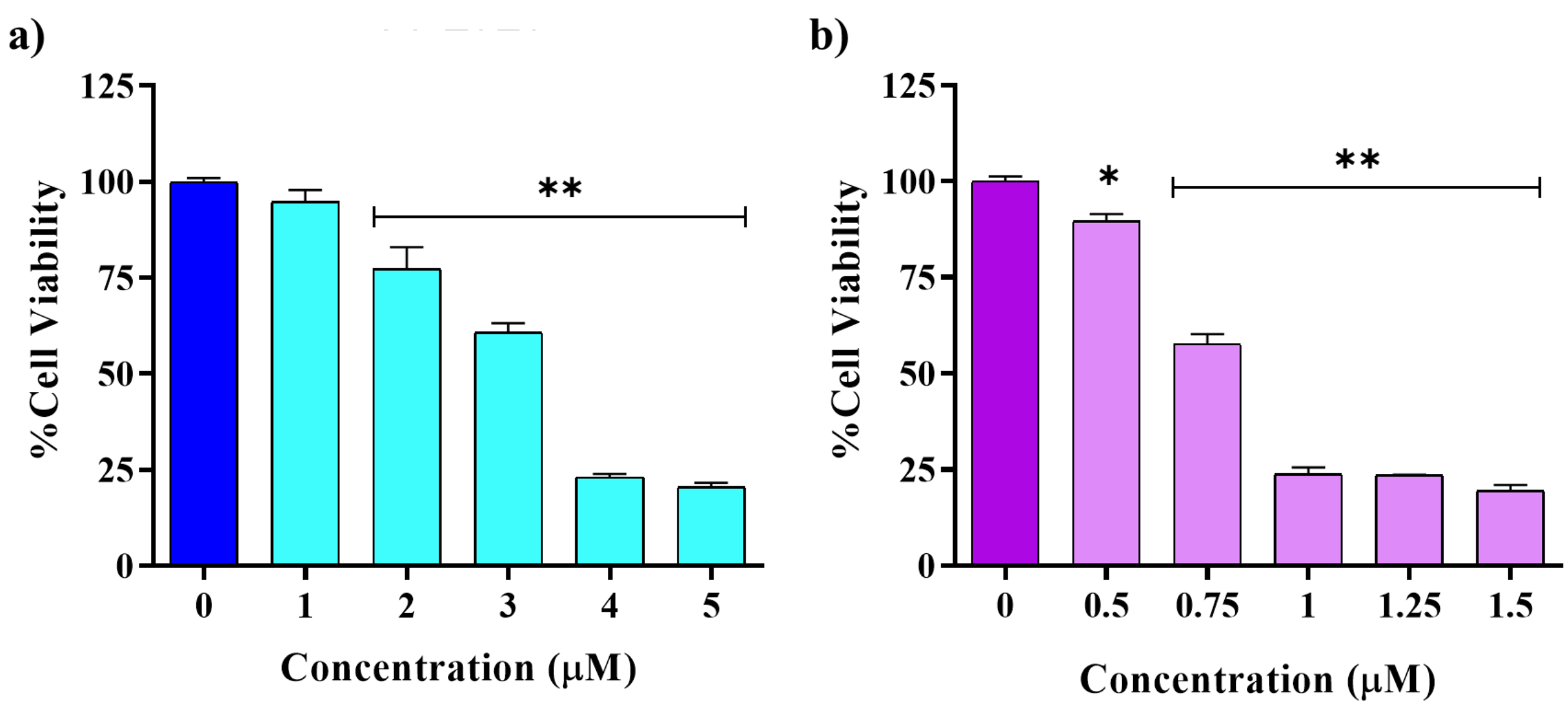

Supplementary Figure 21. Cellular viability was evaluated via MTT assay on a non-tumoral cell line. L929 cells were incubated in DMEM alone (Control) or with different concentrations of a) CuL1 or b)

Cu_2L2_2 for 24 h. The results are expressed as the mean ± SEM. *p <0.001 and **p <0.0001 differences between Control and treatment.

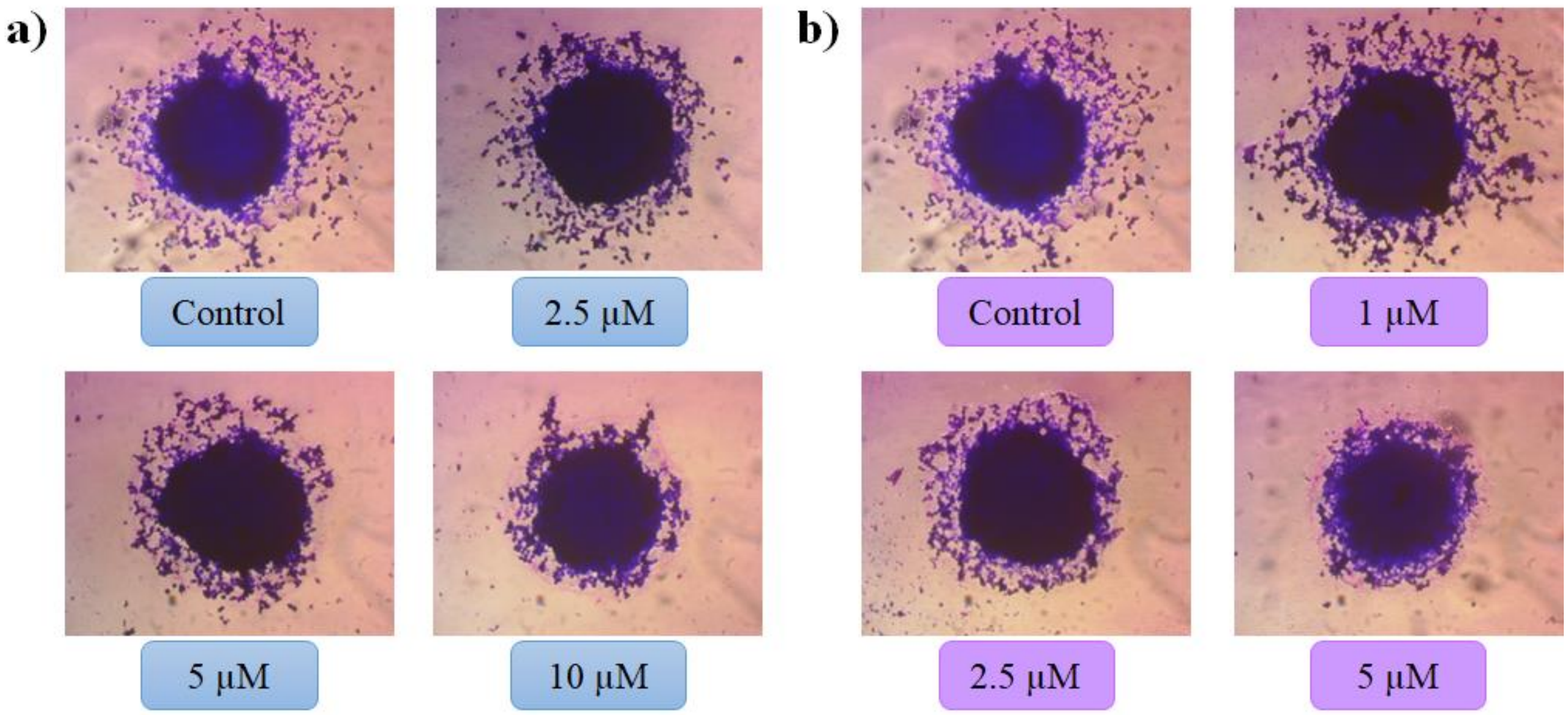

Supplementary Figure 22. Spreading assay of spheroids. Representative images of the spheroids incubated 24 h with 0.5% DMSO in DMEM (Control) or different concentrations of a) CuL1 or b) Cu_2L2_2. Pictures were taken after fixed with methanol and staining process.

1.2 Supplementary Tables

Supplementary Table 1. Bond lengths [Å] and angles (°) of the organic molecules in L1.H_2O.

Molecule 1		Molecule 2	
**Bond**	**Length (Å)**	**Bond**	**Length (Å)**
O(1)-C(19)	1.367(3)	O(2)-C(29)	1.365(4)
O(1)-C(112)	1.371(4)	O(2)-C(212)	1.362(4)
O(11)-C(11)	1.352(4)	O(21)-C(21)	1.355(4)
O(13)-C(18)	1.240(3)	O(23)-C(28)	1.237(3)
N(11)-C(17)	1.286(4)	N(21)-C(27)	1.286(4)
N(11)-N(12)	1.377(3)	N(21)-N(22)	1.387(3)
N(12)-C(18)	1.346(4)	N(22)-C(28)	1.337(4)
C(11)-C(12)	1.390(5)	C(21)-C(22)	1.384(5)
C(11)-C(16)	1.396(4)	C(21)-C(26)	1.408(4)
C(12)-C(13)	1.382(6)	C(22)-C(23)	1.381(5)
C(13)-C(14)	1.385(6)	C(23)-C(24)	1.382(5)
C(14)-C(15)	1.380(5)	C(24)-C(25)	1.369(5)
C(15)-C(16)	1.397(4)	C(25)-C(26)	1.393(4)
C(16)-C(17)	1.453(4)	C(26)-C(27)	1.442(4)
C(18)-C(19)	1.477(4)	C(28)-C(29)	1.459(4)
C(19)-C(110)	1.329(4)	C(29)-C(210)	1.354(5)

C(110)-C(111)	1.421(4)	C(210)-C(211)	1.425(5)
C(111)-C(112)	1.339(5)	C(211)-C(212)	1.331(6)
**Bond**	**Angle (°)**	**Bond**	**Angle (°)**
C(19)-O(1)-C(112)	105.5(2)	C(29)-O(2)-C(212)	106.4(3)
C(17)-N(11)-N(12)	114.4(2)	C(27)-N(21)-N(22)	115.9(2)
C(18)-N(12)-N(11)	120.3(2)	C(28)-N(22)-N(21)	118.8(2)
O(11)-C(11)-C(12)	118.2(3)	O(21)-C(21)-C(22)	118.1(3)
O(11)-C(11)-C(16)	122.1(3)	O(21)-C(21)-C(26)	122.7(3)
C(12)-C(11)-C(16)	119.7(3)	C(22)-C(21)-C(26)	119.2(3)
C(13)-C(12)-C(11)	120.6(3)	C(23)-C(22)-C(21)	121.0(3)
C(12)-C(13)-C(14)	120.5(3)	C(22)-C(23)-C(24)	120.2(3)
C(13)-C(14)-C(15)	118.9(3)	C(25)-C(24)-C(23)	119.2(3)
C(14)-C(15)-C(16)	121.7(3)	C(24)-C(25)-C(26)	122.1(3)
C(11)-C(16)-C(15)	118.6(3)	C(25)-C(26)-C(21)	118.2(3)
C(11)-C(16)-C(17)	122.9(3)	C(25)-C(26)-C(27)	118.8(3)
C(15)-C(16)-C(17)	118.4(3)	C(21)-C(26)-C(27)	122.9(3)
N(11)-C(17)-C(16)	122.1(3)	N(21)-C(27)-C(26)	122.4(3)
O(13)-C(18)-N(12)	125.0(3)	O(23)-C(28)-N(22)	124.2(3)
O(13)-C(18)-C(19)	119.9(3)	O(23)-C(28)-C(29)	119.3(3)
N(12)-C(18)-C(19)	115.1(2)	N(22)-C(28)-C(29)	116.6(2)
C(110)-C(19)-O(1)	110.9(2)	C(210)-C(29)-O(2)	109.9(3)
C(110)-C(19)-C(18)	131.7(3)	C(210)-C(29)-C(28)	129.9(3)
O(1)-C(19)-C(18)	117.4(2)	O(2)-C(29)-C(28)	120.2(3)
C(19)-C(110)-C(111)	106.8(3)	C(29)-C(210)-C(211)	106.2(3)
C(112)-C(111)-C(110)	106.2(3)	C(212)-C(211)-C(210)	106.7(3)
C(111)-C(112)-O(1)	110.7(3)	C(211)-C(212)-O(2)	110.8(3)

Supplementary Table 2. Hydrogen bond distances (Å) and angles (º) in L1.H_2O.

D-H···A	d(D-H)	d(H···A)	d(D···A)	<(DHA)
O(11)-H(11)...N(11)	0.82	1.95	2.671(3)	145.6
N(12)-H(12)...O(1W)	0.86	1.97	2.808(3)	164.0
C(17)-H(17)...O(1W)	0.93	2.38	3.180(4)	144.6
C(112)-H(112)...O(21)#1	0.93	2.52	3.444(4)	171.5
O(21)-H(21)...N(21)	0.82	1.98	2.693(3)	144.4
N(22)-H(22)...O(2W)#2	0.86	2.02	2.864(3)	165.7
C(27)-H(27)...O(2W)#2	0.93	2.57	3.349(4)	142.2
O(1W)-H(1A)...O(23)	0.92(5)	1.92(6)	2.827(3)	167(4)
O(1W)-H(1B)...O(23)#3	0.92(5)	1.85(5)	2.755(3)	170(4)
O(2W)-H(2A)...O(13)	0.96(7)	1.91(7)	2.873(3)	179(5)
O(2W)-H(2B)...O(13)#3	0.89(5)	1.94(5)	2.807(3)	166(5)

Symmetry transformations used to generate equivalent atoms: (#1) x+2, y, z; (#2) x-1, -y+1/2, z+1/2; (#3) x+1, y, z.

Supplementary Table 3. Bond distances (Å) and angles (º) of the organic molecule in L2.H_2O.

Bond	Length (Å)	C(8)-N(2)-N(1)	119.4(4)
O(1)-C(1)	1.355(7)	O(1)-C(1)-C(6)	122.4(4)
O(2)-C(12)	1.370(6)	O(1)-C(1)-C(2)	118.0(5)
O(2)-C(15)	1.411(9)	C(6)-C(1)-C(2)	119.6(5)
O(3)-C(8)	1.235(5)	C(1)-C(2)-C(3)	119.7(5)
N(1)-C(7)	1.287(6)	C(4)-C(3)-C(2)	121.5(5)
N(1)-N(2)	1.388(5)	C(5)-C(4)-C(3)	119.2(5)
N(2)-C(8)	1.342(6)	C(4)-C(5)-C(6)	121.4(5)
C(1)-C(6)	1.395(7)	C(1)-C(6)-C(5)	118.6(4)
C(1)-C(2)	1.394(7)	C(1)-C(6)-C(7)	122.7(4)
C(2)-C(3)	1.384(10)	C(5)-C(6)-C(7)	118.6(4)
C(3)-C(4)	1.364(9)	N(1)-C(7)-C(6)	121.2(4)
C(4)-C(5)	1.373(8)	O(3)-C(8)-N(2)	122.0(4)
C(5)-C(6)	1.405(7)	O(3)-C(8)-C(9)	121.6(4)
C(6)-C(7)	1.445(6)	N(2)-C(8)-C(9)	116.4(4)
C(8)-C(9)	1.475(6)	C(14)-C(9)-C(10)	118.0(4)
C(9)-C(14)	1.387(6)	C(14)-C(9)-C(8)	123.8(4)
C(9)-C(10)	1.406(7)	C(10)-C(9)-C(8)	118.3(4)
C(10)-C(11)	1.364(8)	C(11)-C(10)-C(9)	120.5(5)
C(11)-C(12)	1.395(8)	C(10)-C(11)-C(12)	121.1(5)
C(12)-C(13)	1.368(7)	O(2)-C(12)-C(13)	125.3(5)
C(13)-C(14)	1.392(6)	O(2)-C(12)-C(11)	115.5(5)
**Bond**	**Angle (°)**	C(13)-C(12)-C(11)	119.2(4)
C(12)-O(2)-C(15)	116.7(5)	C(12)-C(13)-C(14)	120.2(4)
C(7)-N(1)-N(2)	115.6(4)	C(13)-C(14)-C(9)	121.1(4)

Supplementary Table 4. Hydrogen bond distances (Å) and angles (º) in L2.H_2O.

D-H···A	d(D-H)	d(H···A)	d(D···A)	<(DHA)
O(1W)-H(1A)...O(3)#1	0.81(7)	2.04(8)	2.843(5)	175(8)
O(1W)-H(1B)...O(3)	0.88(8)	1.92(7)	2.757(5)	157(6)
C(7)-H(7)...O(1W)#2	0.97(5)	2.57(5)	3.336(6)	136(4)
O(1)-H(1O)...N(1)	0.78(11)	1.97(10)	2.648(6)	145(10)
N(2)-H(2N)...O(1W)#2	0.98(7)	1.87(7)	2.832(5)	168(6)

Symmetry transformations used to generate equivalent atoms: (#1) x, y+1, z; (#2) x+1/2, -y+1, z.

Supplementary Table 5. Bond distances (Å) and angles (º) of CuL1.H_2O.

Bond	Length (Å)	O(3)-Cu-O(31)	93.12(11)
Cu-O(1)	1.884(3)	C(12)-O-C(9)	105.6(3)
Cu-N(1)	1.929(3)	C(1)-O(1)-Cu	127.4(2)
Cu-O(3)	1.971(3)	C(8)-O(3)-Cu	111.3(2)
Cu-O(31)	2.002(3)	N(3)-O(31)-Cu	104.1(2)
O-C(12)	1.352(5)	C(7)-N(1)-N(2)	120.1(3)

O-C(9)	1.369(4)	C(7)-N(1)-Cu	128.6(2)
O(1)-C(1)	1.318(4)	N(2)-N(1)-Cu	111.3(2)
O(3)-C(8)	1.251(4)	C(8)-N(2)-N(1)	114.1(3)
O(31)-N(3)	1.292(4)	O(32)-N(3)-O(33)	123.8(3)
O(32)-N(3)	1.214(4)	O(32)-N(3)-O(31)	120.2(3)
O(33)-N(3)	1.237(4)	O(33)-N(3)-O(31)	116.0(3)
N(1)-C(7)	1.280(4)	O(1)-C(1)-C(2)	118.6(3)
N(1)-N(2)	1.379(4)	O(1)-C(1)-C(6)	124.3(3)
N(2)-C(8)	1.337(5)	C(2)-C(1)-C(6)	117.1(3)
C(1)-C(2)	1.409(5)	C(3)-C(2)-C(1)	122.0(3)
C(1)-C(6)	1.429(5)	C(2)-C(3)-C(4)	121.0(3)
C(2)-C(3)	1.369(5)	C(5)-C(4)-C(3)	118.4(3)
C(3)-C(4)	1.399(6)	C(4)-C(5)-C(6)	122.1(3)
C(4)-C(5)	1.373(5)	C(5)-C(6)-C(7)	117.7(3)
C(5)-C(6)	1.405(5)	C(5)-C(6)-C(1)	119.3(3)
C(6)-C(7)	1.431(5)	C(7)-C(6)-C(1)	123.0(3)
C(8)-C(9)	1.453(5)	N(1)-C(7)-C(6)	123.4(3)
C(9)-C(10)	1.353(5)	O(3)-C(8)-N(2)	121.2(3)
C(10)-C(11)	1.416(6)	O(3)-C(8)-C(9)	120.7(3)
C(11)-C(12)	1.348(6)	N(2)-C(8)-C(9)	118.1(3)
**Bond**	**Angle (°)**	C(10)-C(9)-O	110.9(3)
O(1)-Cu-N(1)	92.72(11)	C(10)-C(9)-C(8)	131.7(3)
O(1)-Cu-O(3)	173.92(11)	O-C(9)-C(8)	117.4(3)
N(1)-Cu-O(3)	81.88(11)	C(9)-C(10)-C(11)	105.8(3)
O(1)-Cu-O(31)	91.88(11)	C(12)-C(11)-C(10)	106.5(3)
N(1)-Cu-O(31)	171.23(12)	O-C(12)-C(11)	111.2(4)

Supplementary Table 6. Hydrogen bond distances (Å) and angles (°) in CuL1.H_2O.

D-H···A	d(D-H)	d(H···A)	d(D···A)	<(DHA)
N(2)-H(2N)...O(1W)	0.87(4)	1.85(4)	2.709(4)	168(4)
C(7)-H(7)...O(1W)	0.93	2.57	3.301(4)	135.7
C(10)-H(10)...O(33)#1	0.93	2.52	3.178(5)	128.1
C(11)-H(11)...O(33)#2	0.93	2.55	3.439(5)	159.9
C(12)-H(12)...O(1)#3	0.93	2.60	3.450(5)	151.8
O(1W)-H(1A)...O(1)#4	0.85(1)	1.93(2)	2.782(4)	176(7)
O(1W)-H(1B)...O(31)#3	0.86(1)	2.07(2)	2.904(4)	164(5)

Symmetry transformations used to generate equivalent atoms: (#1) x+1, y, z; (#2) x+3/2, -y+1/2, -z+1; (#3) -x+2, y-1/2, -z+1/2; (#4) -x+1, y-1/2, -z+1/2.

Supplementary Table 7. Bond distances (Å) and angles (°) of Cu_2L2_2.

**Bond**	**Length (Å)**	C(16)-O(4)-Cu	126.9(2)
Cu-N(1)	1.9380(16)	O(1)#1-Cu-O(4)	93.61(7)
Cu-O(1)	1.9601(14)	O(3)-Cu-O(4)	87.33(8)

Cu-O(1)#1	1.9702(14)	N(1)-Cu-Cu#1	131.76(5)
Cu-O(3)	1.9991(15)	O(1)-Cu-Cu#1	40.25(4)
Cu-O(4)	2.371(2)	O(1)#1-Cu-Cu#1	40.00(4)
Cu-Cu#1	3.0052(5)	O(3)-Cu-Cu#1	147.54(5)
O(1)-C(1)	1.343(2)	O(4)-Cu-Cu#1	96.49(6)
O(1)-Cu#1	1.9703(14)	C(1)-O(1)-Cu	127.27(12)
O(2)-C(12)	1.359(3)	C(1)-O(1)-Cu#1	131.82(12)
O(2)-C(15)	1.422(4)	Cu-O(1)-Cu#1	99.74(6)
O(3)-C(8)	1.258(3)	C(12)-O(2)-C(15)	117.5(2)
O(31)-N(3)	1.233(3)	C(8)-O(3)-Cu	112.22(13)
O(32)-N(3)	1.240(3)	C(7)-N(1)-N(2)	118.62(16)
O(33)-N(3)	1.211(3)	C(7)-N(1)-Cu	129.13(14)
N(1)-C(7)	1.276(3)	N(2)-N(1)-Cu	112.11(12)
N(1)-N(2)	1.373(2)	C(8)-N(2)-N(1)	115.26(16)
N(2)-C(8)	1.328(3)	O(33)-N(3)-O(31)	121.0(3)
C(1)-C(2)	1.396(3)	O(33)-N(3)-O(32)	119.0(2)
C(1)-C(6)	1.417(3)	O(31)-N(3)-O(32)	120.0(2)
C(2)-C(3)	1.382(3)	O(1)-C(1)-C(2)	119.96(18)
C(3)-C(4)	1.388(4)	O(1)-C(1)-C(6)	122.37(18)
C(4)-C(5)	1.371(4)	C(2)-C(1)-C(6)	117.67(19)
C(5)-C(6)	1.410(3)	C(3)-C(2)-C(1)	121.6(2)
C(6)-C(7)	1.443(3)	C(2)-C(3)-C(4)	121.0(2)
C(8)-C(9)	1.475(3)	C(5)-C(4)-C(3)	118.5(2)
C(9)-C(10)	1.389(3)	C(4)-C(5)-C(6)	121.9(2)
C(9)-C(14)	1.401(3)	C(5)-C(6)-C(1)	119.25(19)
C(10)-C(11)	1.379(3)	C(5)-C(6)-C(7)	115.68(19)
C(11)-C(12)	1.381(3)	C(1)-C(6)-C(7)	125.05(18)
C(12)-C(13)	1.399(4)	N(1)-C(7)-C(6)	123.75(18)
C(13)-C(14)	1.372(3)	O(3)-C(8)-N(2)	119.87(18)
O(4)-C(16)	1.401(4)	O(3)-C(8)-C(9)	120.84(19)
C(16)-C(17)	1.471(5)	N(2)-C(8)-C(9)	119.29(18)
**Bond**	**Angle (°)**	C(10)-C(9)-C(14)	118.5(2)
N(1)-Cu-O(1)	91.51(6)	C(10)-C(9)-C(8)	118.14(19)
N(1)-Cu-O(1)#1	171.75(7)	C(14)-C(9)-C(8)	123.4(2)
O(1)-Cu-O(1)#1	80.26(6)	C(9)-C(10)-C(11)	121.3(2)
N(1)-Cu-O(3)	80.54(7)	C(10)-C(11)-C(12)	119.9(2)
O(1)-Cu-O(3)	171.08(6)	O(2)-C(12)-C(13)	115.7(2)
O(1)#1-Cu-O(3)	107.71(6)	O(2)-C(12)-C(11)	124.7(2)
N(1)-Cu-O(4)	86.66(7)	C(13)-C(12)-C(11)	119.6(2)
O(1)-Cu-O(4)	96.31(7)	C(14)-C(13)-C(12)	120.2(2)
C(13)-C(14)-C(9)	120.5(2)	O(4)-C(16)-C(17)	114.4(3)

Supplementary Table 8. Hydrogen bond distances (Å) and angles (º) in Cu_2L2_2.

D-H···A	d(D-H)	d(H···A)	d(D···A)	<(DHA)
N(2)-H(2)...O(32)#2	0.86	1.93	2.769(2)	165
C(2)-H(2A)...O(3)#1	0.93	2.22	3.076(3)	153
C(7)-H(7)...O(32)#2	0.93	2.60	3.351(3)	138
O(4)-H(4O)...O(31)#3	0.86(1)	2.44(2)	3.253(3)	158(2)
O(4)-H(4O)...O(33)#3	0.86(1)	2.36(2)	3.128(4)	149(3)

Symmetry transformations used to generate equivalent atoms: (#1) -x+1, -y+2, -z; (#2) -x+1, y-1/2, -z+1/2; (#3) x, y-1, z.
